# Molecularly Imprinted Polymers for Biosensing: From Synthetic Recognition to Integrated Biointerfaces

**DOI:** 10.3390/mi17091037

**Published:** 2026-08-29

**Authors:** Giovanna Di Pasquale, Antonino Pollicino

**Affiliations:** 1 Department of Chemical Sciences, University of Catania, V.le A. Doria 6, 95125 Catania, Italy; 2 Department of Civil-Industrial Engineering and Architecture, University of Catania, V.le A. Doria 6, 95125 Catania, Italy

**Keywords:** molecularly imprinted polymers, biosensors, synthetic receptors, epitope imprinting, electrochemical transduction, wearable sensors, point-of-care diagnostics, machine-learning-assisted design

## Abstract

Molecularly imprinted polymers (MIPs) are synthetic receptors with cavities shaped around a template, combining antibody-like selectivity with chemical, thermal, and mechanical robustness; low cost; and reusability. This Review examines recent advances in MIP-based biosensing, from bulk materials to thin-film, nanostructured, surface-imprinted, and epitope-imprinted architectures designed to improve site accessibility and performance in complex biofluids. We connect polymer chemistry and interface design to molecular recognition and electrochemical, optical, and mass-sensitive transduction. Applications range from small molecules, proteins, nucleic acids, and viruses to whole cells, encompassing miniaturized, wearable, and point-of-care formats. Particular attention is devoted to design assisted by computational methods and machine learning, as well as to the challenges of reproducibility, standardization, metrology, and sustainability that still limit translation. Rather than universal substitutes for antibodies, MIPs are presented as programmable biointerfaces that integrate molecular recognition, signal transduction, device engineering, and the design of low-environmental-impact materials.

## 1. Introduction

The precise and selective recognition of target molecules lies at the core of biosensing and bioanalytical chemistry, as the analytical performance of any sensing platform is ultimately dictated by the affinity and specificity of its recognition element [1]. From antibody-based immunoassays to aptamers and other synthetic receptors, the field has continuously sought materials that combine high selectivity with robustness, reproducibility, and cost-effective production [2]. Natural recognition elements such as antibodies, however, remain limited by intrinsic fragility, batch-to-batch variability, and reduced stability under variations in temperature, pH, and ionic strength [3]. These limitations have driven increasing interest in fully synthetic recognition systems—most notably MIPs—engineered via template-directed polymerization to generate stable, antibody-like binding sites with enhanced chemical robustness and suitability for operation under harsh or variable conditions [4].

**Figure 1:**
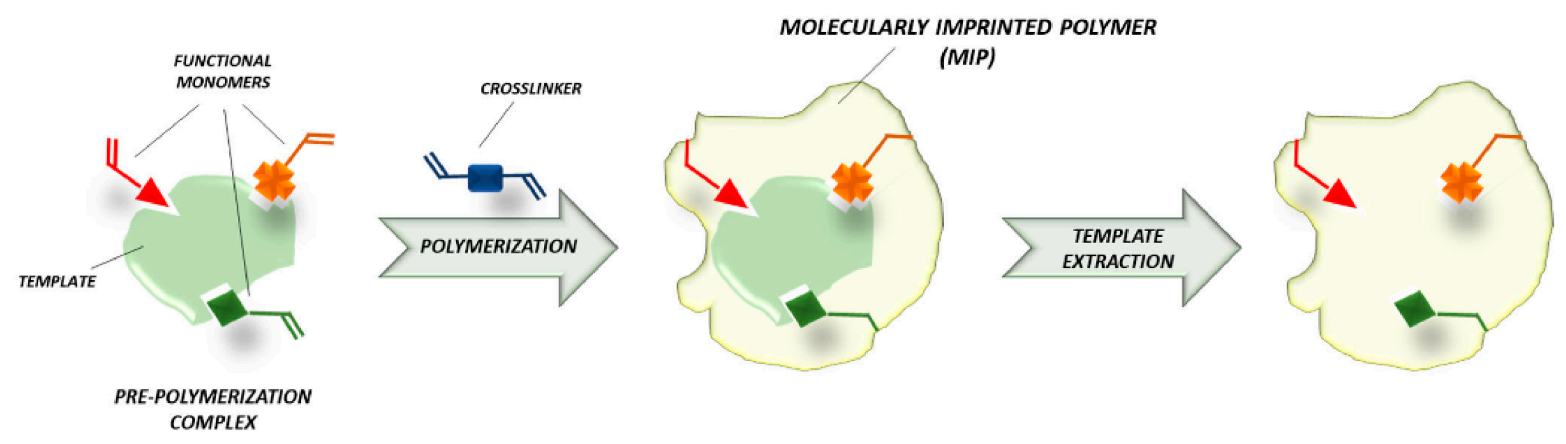
Schematic representation of the molecular imprinting mechanism: the functional monomers self-assemble around the template molecule forming the pre-polymerization complex; polymerization in the presence of the cross-linking agent fixes the arrangement of the functional groups and the subsequent removal of the template generates recognition cavities complementary in shape, size and chemical functionality, equipped with “molecular memory” and capable of selectively re-binding the target. Reproduced from Parisi et al. [7], J. Funct. Biomater. 2022, 13, 12, under the CC BY 4.0 license.

Within this context, MIPs have emerged as one of the most versatile classes of synthetic receptors [5]. A MIP is a polymeric network containing molecularly engineered cavities that are complementary in shape, size, and chemical functionality to a selected target (template) molecule. During polymerization, functional monomers self-assemble around the template through non-covalent, covalent, or semi-covalent interactions; subsequent template removal leaves behind recognition sites that retain a molecular “memory” of the target and are capable of selective rebinding [6] (Figure 1).

Introduced in the 1970s for chromatographic separation and subsequently extended to solid-phase extraction (SPE), synthetic-polymer molecular imprinting has since evolved toward biosensing and point-of-care applications. This transition was enabled by the recognition that the selective cavities responsible for chromatographic retention could also act as active interfaces when coupled to suitable transducers, including electrochemical, optical, and mass-sensitive platforms. Over the past two decades, this conceptual shift has transformed MIPs from passive sorbents into functional recognition layers capable of generating or modulating measurable analytical signals [8]. A MIP-based biosensor can thus be defined as an analytical device in which a molecularly imprinted polymer acts as the recognition element—integrated with, or deposited directly onto, a transducer that converts the selective rebinding of the target into a measurable electrical, optical, or mass-related signal. The term “biosensor” is retained here in its applied sense, denoting the detection of biologically or clinically relevant analytes, even though the recognition element is fully synthetic rather than biological.

Today, MIP-based sensors have been applied to a wide range of targets, spanning small molecules (drugs, pesticides, hormones), biomarkers, proteins, viruses, and even whole cells. These applications cover diverse domains, from clinical diagnostics and food safety to environmental monitoring. In particular, the replacement of fragile biological receptors with stable and inexpensive synthetic analogues is highly attractive for portable, wearable, and in-field sensing devices, where long-term stability, mechanical robustness, and resistance to denaturation are critical [9].

The versatility of MIPs stems from their fully synthetic backbone, which—depending on the formulation—tolerates a broad range of pH, temperature, and solvent conditions and withstands repeated regeneration cycles with limited degradation, an affinity–stability trade-off with respect to biological receptors that is examined in Section 2.6. The broad availability of functional monomers, crosslinkers, and polymerization routes allows affinity and selectivity to be tailored to a wide variety of targets [10,11]. Moreover, MIPs can be processed into thin films, nanoparticles, hydrogels, or conformal coatings, ensuring compatibility with diverse substrates and device architectures [6,10].

Early-generation MIPs were limited by heterogeneous binding-site distributions, slow mass transport, and reduced performance in aqueous media, largely owing to solvent competition and limited site accessibility [10]. Recent advances in polymer chemistry and nanofabrication have substantially mitigated these limitations. Modern strategies include surface and epitope imprinting to generate thin and accessible recognition layers, nano- and micro-structured MIPs designed to enhance mass transfer, electropolymerized MIP films grown directly on electrode surfaces, and hybrid MIP–nanomaterial composites that improve signal transduction and amplification. In parallel, the development of water-compatible and stimuli-responsive polymers has enabled reliable MIP operation under physiological conditions [12]; these architectural strategies are examined individually in Section 3.

**Figure 2:**
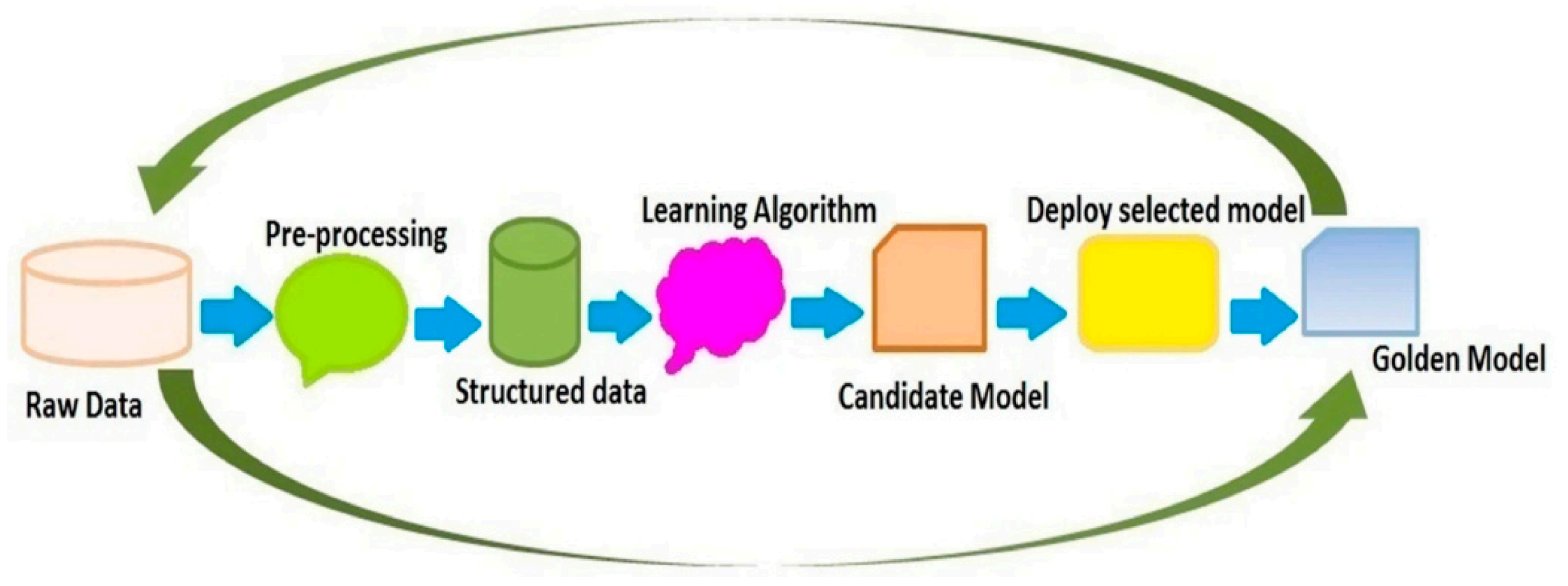
Workflow of a data-driven/ML approach applied to molecular imprinting: collection of experimental data (monomer type, template, solvent, and synthesis conditions), pre-processing, application and comparison of regression and ensemble algorithms, and iterative refinement of the model for imprinting quality prediction (imprinting factor). Reproduced from Yarahmadi et al. [13], Sci. Rep. 2023, 13, 12111, under the CC BY 4.0 license.

Beyond these materials-level advances, artificial intelligence (AI) and machine learning (ML) are beginning to reshape how molecular imprinting is designed and interpreted, although their impact remains at an early and still largely exploratory stage. Rather than relying on heuristic optimization and predefined interaction models, data-driven approaches can learn complex relationships between monomer chemistry, polymer architecture, and binding performance directly from experimental datasets [13] (Figure 2).

This can support predictive identification of candidate functional monomers, crosslinker ratios, and imprinting conditions, reducing empirical trial-and-error and reframing molecular imprinting as a more rational, data-guided materials design problem [13]. Early demonstrations of this approach include AI-assisted design strategies applied to optimize polymers targeting complex biomarkers such as C-reactive protein [14], although such studies remain proof-of-concept and rest on limited, non-standardized datasets. In parallel, AI- and ML-assisted signal processing is increasingly integrated into electrochemical and optical MIP platforms, where it can correct baseline drift, improve signal-to-noise ratio, and extract analyte-specific features [15]. As a result, MIP-based sensors are evolving into hybrid chemical–digital systems in which molecular recognition and data analytics are increasingly interconnected; these design and signal-processing roles, together with the data infrastructure they require, are developed critically in Section 2.8, Section 9.7 and Section 11.1.

In the broader landscape of biosensor technologies, MIPs occupy a strategic position between biological recognition elements, which offer high specificity but limited robustness, and purely physicochemical sensing approaches, which are stable but often lack selectivity. Their adaptability has enabled integration into transduction platforms ranging from electrochemical and plasmonic devices to acoustic-wave resonators, field-effect transistors, and wearable sensing patches, which are treated systematically in Section 5, Section 6, Section 7 and Section 8 [16].

Despite the growing number of successful proof-of-concept demonstrations, large-scale commercial adoption of MIP-based biosensors—particularly in clinical diagnostics—remains limited [16,17,18]. This gap between academic maturity and market penetration reflects challenges related to reproducibility, standardization of imprinting protocols, and long-term validation under application-relevant conditions, as well as regulatory frameworks historically optimized for biological recognition elements [16,18,19].

Accordingly, MIPs are increasingly positioned not as universal replacements for immunoassays, but rather as enabling technologies for sensing scenarios in which robustness, cost efficiency, and long-term operational stability outweigh the need for ultimate binding affinity [17,18]. Recent point-of-care and biomarker-oriented MIP platforms illustrate this dual character—clear technological promise alongside the translational barriers just outlined [16,17].

Numerous recent reviews have surveyed MIP-based sensors, most of them organised by transducer type or by target class and largely devoted to cataloguing analytical performance. The present Review is instead structured as a single causal chain—from the physicochemical basis of recognition, through polymer chemistry, architecture, and transduction, to target class, device integration, metrology, and sustainability—so that each design choice is traced to its consequence for the final device. Throughout, the maturity of each approach is graded explicitly—from established, through emerging, to proof-of-concept—so that individual claims are calibrated accordingly. Two threads run throughout and are treated critically rather than promotionally: the reproducibility, standardisation, and reporting problem that still separates academic prototypes from validated devices—for which we argue that minimum-information reporting protocols, analogous to those adopted in other analytical fields, are now a prerequisite for translation (Section 9.2)—and the emerging role of computational and machine-learning-assisted design and signal processing. Sustainability and life-cycle considerations are integrated as a first-class dimension rather than an afterthought.

The literature surveyed in this Review was identified through structured searches of Scopus, combining the keywords “molecularly imprinted polymer”, “MIP”, “biosensor”, “biosensing”, and “molecular recognition” with transducer- and target-specific terms (e.g., “electrochemical”, “epitope imprinting”, “green synthesis”). Priority was given to peer-reviewed original research and reviews published between January 2023 and mid-2026, to capture the most recent developments, while a limited number of earlier, seminal contributions were retained to establish conceptual foundations. Sources were selected based on relevance to MIP-based biosensing, methodological soundness, and the availability of quantitative binding or analytical data; conference abstracts, non-peer-reviewed preprints, and reports lacking experimental validation were generally excluded. As a critical rather than systematic review, this work does not claim exhaustive coverage but aims to represent the principal materials, transduction, and integration strategies that define the current state of the field.

The Review is organised as follows. Section 2 recalls the physicochemical basis of imprinting and recognition; Section 3 surveys the architectures and formats through which the resulting cavities are made accessible to a transducer; and Section 4 examines the polymer chemistry—monomers, crosslinkers, porogens, and nanocomposite matrices—that underpins both. Section 5 and Section 6 address electrochemical and optical/mass-sensitive transduction, respectively, while Section 7 reorganises the field by target class, from small molecules to whole cells. Section 8 considers integration, miniaturisation, and wearable formats; Section 9 addresses reliability, metrology, and standardisation; Section 10 discusses sustainability and green design; and Section 11 outlines the outlook.

**Figure 3:**
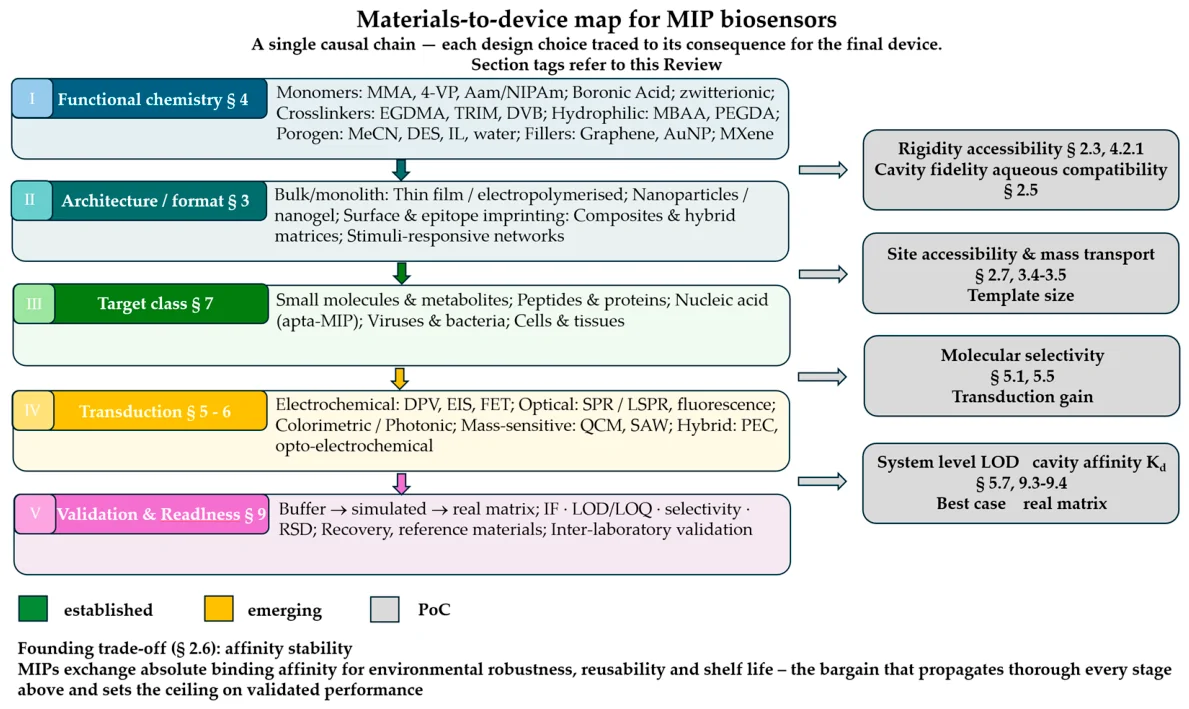
Materials-to-device map for MIP biosensors. The Review is structured as a single causal chain in which each design choice—functional chemistry (Section 4), architecture (Section 3), target class (Section 7), transduction (Section 5 and Section 6), and validation/readiness (Section 9)—is traced to its consequence for the final device. The trade-off governing each transition is annotated below the corresponding arrow, with the founding affinity–stability trade-off (Section 2.6) underlying the whole chain. Readiness is graded as established, emerging, or proof-of-concept.

Figure 3 summarises this causal chain as a materials-to-device map, annotating the trade-off that governs each transition.

## 2. Fundamentals of Molecular Imprinting and Recognition Mechanisms

Having outlined in the Introduction what molecular imprinting achieves, this section examines why it works: the interactions that pre-organise the binding site, the network parameters that preserve it after template removal, and the thermodynamic and kinetic descriptors used to quantify the result [20,21]. This approach provides a synthetic route to artificial receptors that mimic biological recognition phenomena—such as enzyme–substrate or antigen–antibody binding—yet are built entirely from abiotic components [21]. The interplay between chemical functionality, spatial arrangement, and polymer network rigidity governs the strength, specificity, and reversibility of the recognition process [22].

### 2.1. The Imprinting Process: From Pre-Organization to Recognition

Molecular imprinting is implemented through a templating process in which specific molecular interactions established during polymer formation are translated into permanent recognition features within the polymer network [20].

In a suitable porogenic medium, the target molecule associates with functional monomers through covalent or, more frequently, non-covalent interactions, leading to a transient pre-organization that defines the chemical functionality and spatial arrangement of the nascent binding site [21].

Subsequent polymerization in the presence of a crosslinker immobilizes this monomer–template assembly within a rigid three-dimensional architecture, thereby encoding the molecular information of the template into the polymer matrix.

Template removal unveils binding cavities that are complementary to the target in terms of size, shape, and interaction chemistry. Upon re-exposure to the analyte, these sites promote selective rebinding via the same interaction motifs that governed the initial complex formation, enabling specific molecular recognition.

This reversible templating–de-templating cycle is the physical basis of the molecular memory that lets imprinted polymers act as fully synthetic receptors.

### 2.2. Intermolecular Interactions Governing Imprinting

The robustness and fidelity of the final polymer are largely determined by the nature and geometry of the monomer–template interactions established in the pre-polymerisation mixture [23]. These interactions govern both the fidelity of the resulting cavity and the range of media in which recognition can be sustained and therefore dictate solvent compatibility and selectivity under realistic detection conditions.

#### 2.2.1. Non-Covalent Imprinting

Non-covalent imprinting represents the most widely adopted strategy and relies on hydrogen bonding, electrostatic interactions, π–π stacking, dipole–dipole forces, and hydrophobic effects to stabilize the template–monomer complex prior to polymerization [23]. Its main advantages include simplicity of complex formation and broad applicability to structurally diverse targets. Because these interactions are individually weak, they are readily disrupted by increases in ionic strength or solvent polarity; water in particular competes for hydrogen-bond donors and acceptors and screens electrostatic pairing, which limits performance in aqueous and biological media (Section 2.5) [23]. Typical systems employ functional monomers such as methacrylic acid (MAA), acrylamide (AAm), 4-vinylpyridine (4-VP), or urea-based derivatives, capable of forming hydrogen-bonded or electrostatic complexes with carboxylic acid, amide, or phenolic groups in the template molecule [23].

#### 2.2.2. Covalent and Semi-Covalent Imprinting

Covalent imprinting involves the formation of reversible covalent bonds—such as boronate esters, Schiff bases, or acetal linkages—between the template and functional monomers during polymerization, yielding highly uniform and well-defined binding sites [24]. While this approach improves site homogeneity and reproducibility, it is often associated with slower rebinding kinetics due to the need for bond reformation. Semi-covalent strategies overcome this limitation by combining covalent interactions during pre-assembly with non-covalent interactions during rebinding, thereby achieving a balance between structural precision and practical recognition efficiency [24].

#### 2.2.3. Supramolecular and Hybrid Interactions

More advanced MIP designs increasingly exploit supramolecular recognition motifs, including crown ethers, cyclodextrins, calixarenes, and metal–ligand coordination, to introduce directionality and tunable binding affinity [24]. Integrating such pre-organised hosts into the network therefore provides one route to selective recognition in aqueous and complex matrices, complementing the epitope-, monomer-, and interface-based strategies discussed in Section 2.5 [24].

### 2.3. Role of Crosslinking and Porogen

The crosslinker plays a dual role: it defines the mechanical rigidity of the polymer matrix and stabilizes the spatial configuration of recognition sites. Typical crosslinkers include ethylene glycol dimethacrylate (EGDMA), trimethylolpropane trimethacrylate (TRIM), and divinylbenzene (DVB). Their content must be carefully balanced. High crosslink densities (typically 60–80 mol%) preserve cavity geometry over time but yield networks so dense that analyte diffusion is hindered, whereas lower densities improve accessibility at the expense of geometric stability, with cavities liable to relax or collapse [21]. The consequences of this trade-off for specific crosslinker chemistries are examined in Section 4.2.

The porogenic solvent determines the morphology and accessibility of binding sites. Aprotic porogens of low hydrogen-bonding capacity—ranging from apolar media such as toluene and chloroform to the polar-aprotic acetonitrile—favour well-defined non-covalent complexes, because they do not compete for the template–monomer hydrogen bonds, whereas protic or strongly hydrogen-bonding solvents disrupt pre-organization while yielding more hydrophilic polymers. Controlling this delicate balance between site fidelity and diffusion dynamics is essential for biosensing, where rapid and reversible recognition is required [25].

### 2.4. Structural and Thermodynamic Parameters

The recognition behavior of MIPs can be quantitatively described using binding isotherms analogous to those employed in biochemistry and molecular recognition studies. Experimental binding data are commonly expressed as the equilibrium binding capacity, Q, and fitted using Langmuir, Freundlich, or Langmuir–Freundlich (Sips) models to extract key parameters such as the apparent dissociation constant, $K_{d}$, and the maximum binding capacity, $Q_{max}$ [26,27]. The choice of isotherm should, however, be justified by its underlying assumptions rather than by the goodness of fit alone: the Langmuir model presumes energetically equivalent, non-interacting sites, whereas the Freundlich and Sips models explicitly accommodate site heterogeneity, so that a satisfactory fit describes the uptake data empirically but does not, in itself, demonstrate a homogeneous or heterogeneous cavity population [26,27].

The imprinting factor (IF), defined as

(1) $$IF=\frac{Q_{MIP}}{Q_{NIP}},$$

where $Q_{MIP}$ and $Q_{NIP}$ represent the equilibrium binding capacities of the imprinted and non-imprinted polymers (NIPs) measured under identical conditions, is widely used to quantify the selectivity enhancement achieved through imprinting [26]. In experimentally optimized non-covalent MIP systems, IF values exceeding approximately 3 are generally associated with the effective formation of specific recognition cavities, whereas lower values often indicate a significant contribution from non-specific adsorption [26]. This threshold is only indicative: because the IF is a ratio of capacities, its numerical value depends on the conditions under which Q_MIP_ and Q_NIP_ are measured—template concentration, incubation time, temperature, and the assay format itself—so IF values are not directly comparable across studies unless these conditions are matched, and well-performing surface- and protein-imprinted sensors are occasionally reported with IF < 3. The matched NIP is a necessary control for separating imprinting from non-specific uptake, but it is not sufficient to establish molecular selectivity, which must be demonstrated separately against structurally related analogues, competing interferents, and, ultimately, real matrices (Section 9.4).

It is essential to keep these material-level descriptors distinct from the analytical figures reported at the device level. Binding affinity (K_d_ or K_a_) and binding capacity (Q_max_) characterise the recognition pair; the IF quantifies the imprinting effect relative to the NIP; while analytical sensitivity (the calibration slope), the limits of detection and quantification (LOD/LOQ) and selectivity/cross-reactivity are properties of the complete sensor. These quantities are not interchangeable—in particular, a low LOD does not by itself imply a correspondingly low K_d_, since it also reflects transduction gain, noise definition and the statistical treatment of the calibration data (Section 5.7, Section 9.3 and Section 9.4)—and they are used with this distinction throughout the Review.

Thermodynamic analyses further elucidate the driving forces governing MIP–analyte interactions. Temperature-dependent binding studies evaluated through van’t Hoff analysis have shown that recognition in non-covalent MIPs is frequently enthalpy-driven, reflecting the dominant contribution of hydrogen bonding and electrostatic interactions [28], while systems in which hydrophobic effects prevail tend to exhibit a stronger entropic contribution [29]. Understanding these thermodynamic signatures is crucial for the rational design of MIPs, and becomes decisive in aqueous media, where solvation and competitive interactions dominate binding performance—the problem addressed in Section 2.5 [27].

### 2.5. Molecular Imprinting in Water and Biological Media

A major challenge in translating MIP technology to biosensing lies in performing imprinting and recognition in aqueous environments, where water competition erodes binding-site fidelity (Section 2.2.1). To address this limitation, several strategies have been developed. The first is to template the cavity not on the intact analyte but on a short, solvent-exposed peptide signature (an epitope), whose smaller size and higher solubility make aqueous pre-organisation tractable; the design principles and macromolecular scope of this strategy are developed in Section 3.5. A concrete illustration is provided by water-soluble MIP nanogels templated on peptide epitopes, which achieve selective hCG recognition with nanomolar affinity under aqueous conditions, demonstrating that imprinting short peptide fragments rather than full proteins can enhance selectivity and binding performance in physiological media [30].

**Figure 4:**
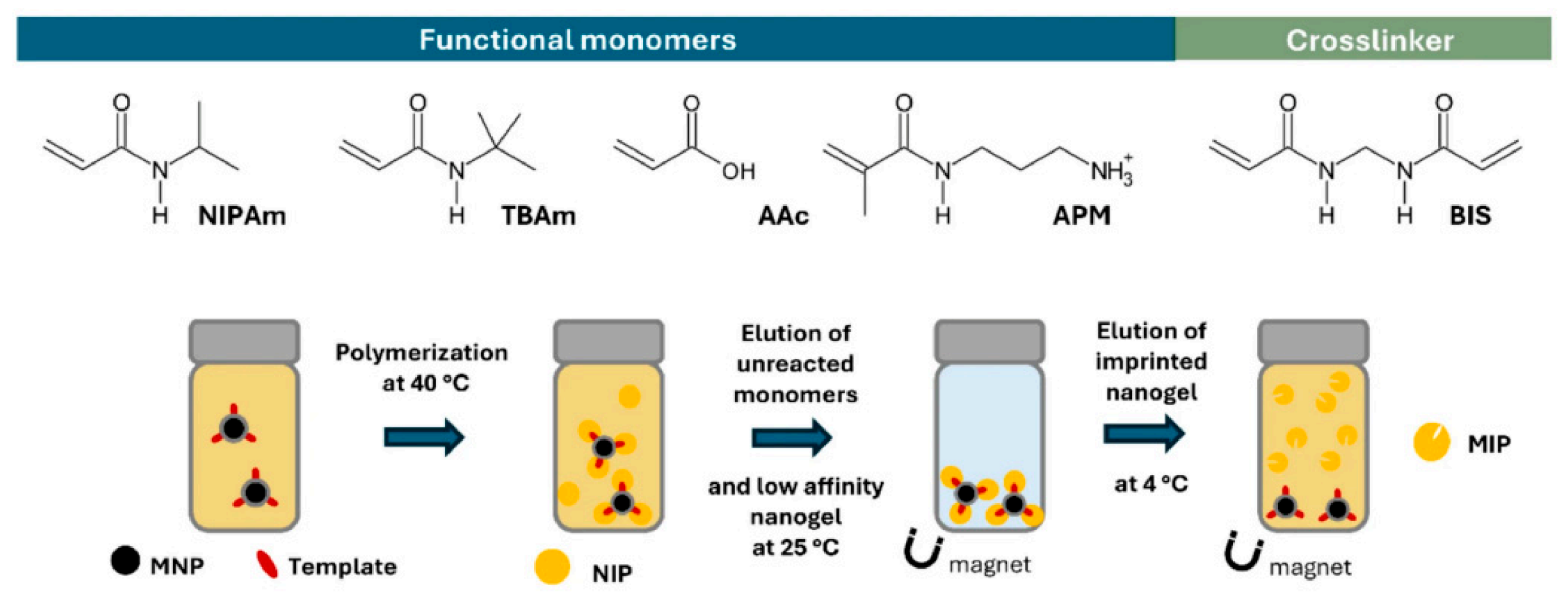
Principle of aqueous epitope imprinting for human chorionic gonadotropin (hCG): water-soluble functional monomers assemble around surface-immobilized peptide epitopes (magnetic dispersed-phase templating) and, after template removal, yield imprinted nanogels whose cavities rebind the target with nanomolar affinity in physiological media. Reproduced from Zubrytė et al. [30], Sci. Rep. 2025, 15, 10436, under CC BY 4.0.

A complementary, materials-level route—amphiphilic or zwitterionic compositions together with surface confinement that preserve cavity integrity in serum, saliva, or cell-culture media—addresses the same solvent-competition problem and is developed in Section 3.7 [30,31]. The aqueous epitope-imprinting strategy is illustrated in Figure 4.

### 2.6. Comparing MIPs to Natural Receptors

While MIPs mimic biological recognition, their mechanism differs fundamentally from that of enzymes or antibodies. In biological systems, molecular recognition often involves induced-fit and dynamic conformational adaptation, whereas conventional, highly crosslinked MIPs operate predominantly through preorganized, comparatively rigid cavities. This picture should not be generalised to the whole MIP family: nanogels, hydrogels, and stimuli-responsive networks (Section 3.3 and Section 3.7) are deliberately soft, swollen, or adaptive and can display intermediate conformational flexibility between classical bulk MIPs and biological receptors. This structural difference generates a general trade-off between absolute affinity, which is typically lower in MIPs than in natural systems, and environmental robustness, which is usually superior in MIPs because the crosslinked network does not undergo protein-like denaturation and, depending on formulation, tolerates a broader range of temperature, pH, and solvent conditions. MIPs are also more readily regenerated, since emptying the cavities by washing is considerably simpler than restoring a spent biological receptor (Table 1); it is this pairing of lower absolute affinity with superior durability, regeneration, and shelf life that defines the synthetic advantage exploited throughout the Review.

Table 1 lists how these differences materialize in the main biological, biomimetic, and synthetic recognition elements.

From an application standpoint, none of these recognition elements is universally preferable, and their choice should be guided by the dominant constraints of the intended detection scenario. Antibodies remain the most appropriate choice when recognition of complex conformational protein epitopes is required, combined with high affinity that is consistently well characterized, particularly in established clinical assays for which validated reagents and workflows are already available and controlled storage conditions are acceptable. Aptamers are particularly attractive when a sequence-defined receptor is needed that can be chemically synthesized and readily modified, for example in miniaturized electrochemical or optical platforms; their reversible folding and high batch-to-batch reproducibility are advantages, although performance may depend on ionic strength and folding conditions, and unmodified nucleic acid aptamers may remain susceptible to nuclease degradation. MIPs become especially advantageous when the primary requirements are chemical and thermal robustness, long shelf life, repeated regeneration, low-cost scalable fabrication, or operation outside the conditions compatible with biological receptors. They are also particularly appealing for targets consisting of small molecules or for analytes for which generating suitable antibodies or aptamers is difficult or expensive. Receptor selection should therefore be based not on affinity alone, but on a multiparametric trade-off involving target class, required analytical performance, matrix complexity, operating environment, device lifetime, manufacturability, and validation requirements. Hybrid recognition architectures may offer a further option when the complementary advantages of biological and synthetic receptors justify the added fabrication complexity.

### 2.7. Recognition Kinetics and Diffusion Limitations

Unlike molecular recognition in free solution, MIP–analyte interactions are often diffusion-limited: because binding sites are heterogeneously distributed throughout a rigid crosslinked network, the analyte must diffuse through the polymer porosity before reaching an accessible cavity so that the observed response time reflects intraparticle mass transport rather than the intrinsic association rate. Uptake curves are frequently fitted with pseudo-first-order, pseudo-second-order, or diffusion-controlled expressions, with response times depending on polymer morphology and template size; these models are descriptive rather than mechanistic, however, since a good fit summarises the observed uptake without resolving the overlapping contributions of diffusion, mass transfer, and site heterogeneity, and it should not, on its own, be read as evidence for a specific molecular association mechanism. Reducing particle size to the nanoscale has been shown to enhance analyte access to imprinted sites and improve binding kinetics by decreasing diffusion barriers [44,45]. Nanostructuring and surface confinement of the recognition sites are the two established remedies, since both shorten the diffusion path to a few nanometres; the corresponding material formats are treated in Section 3.3 and Section 3.4 [44,45].

**Figure 5:**
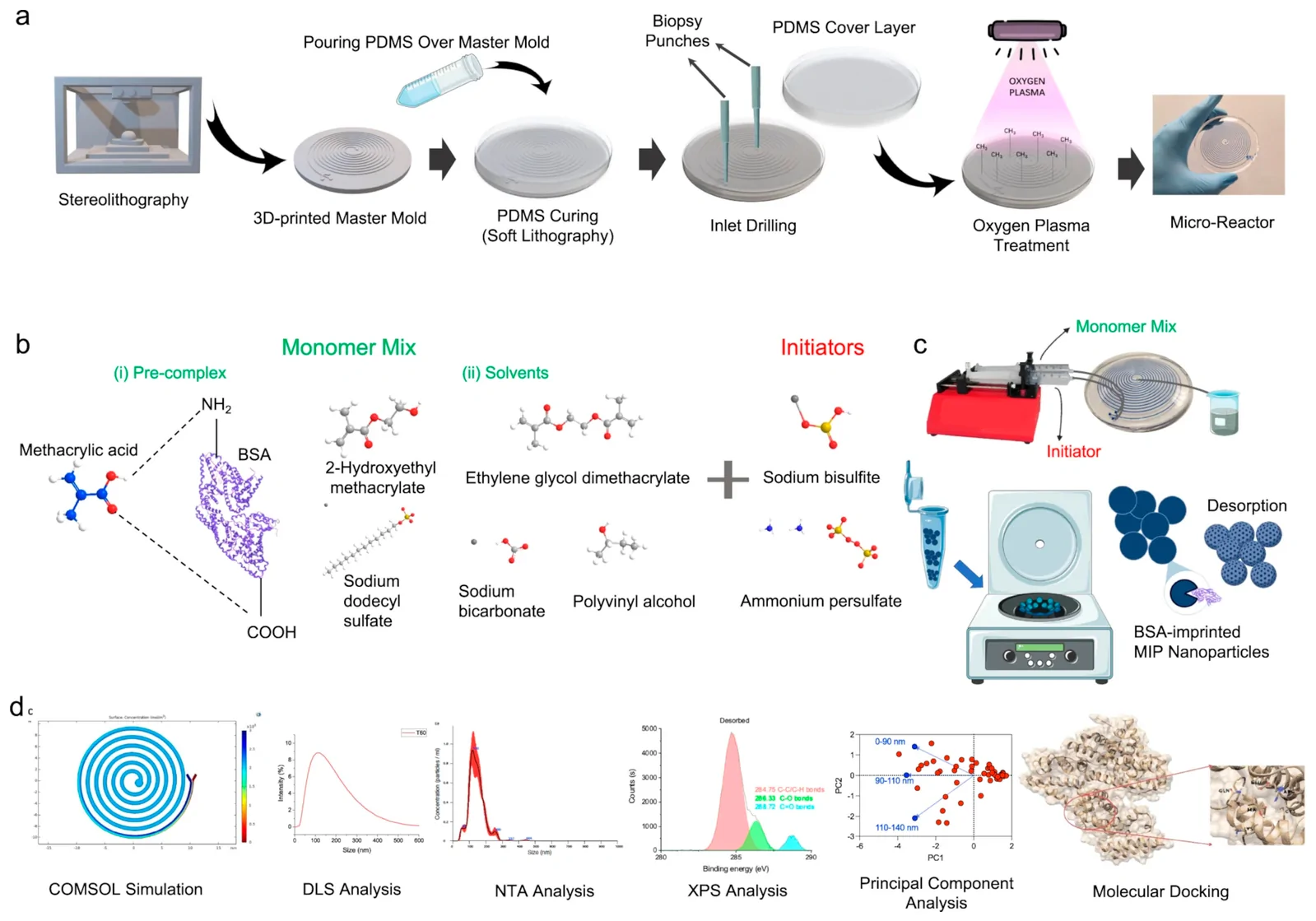
Micro-reactor platform for the in situ, continuous synthesis of molecularly imprinted polymer nanoparticles: template and functional monomers are combined under controlled flow to generate, within minutes, large numbers of size-controlled imprinted nanoparticles whose surface-confined cavities allow rapid analyte rebinding; monomer–template interactions were further examined by molecular docking and dynamics simulations. (**a**) The fabrication of the system, (**b**) chemical composition of MIP nanoparticles, and (**c**) preparation and (**d**) characterization of BSA-imprinted nanoparticles are demonstrated, respectively. Reproduced from Erdem et al. [44], Nat. Commun. 2023, 14, 4840, under CC BY 4.0.

A representative micro-reactor approach for the in situ, continuous production of such imprinted nanoparticles—together with the molecular docking/dynamics analysis of the monomer–template interactions—is shown in Figure 5.

For biosensors, where rapid signal generation is essential, these design refinements have proven decisive in moving from static equilibrium assays to real-time, label-free detection.

### 2.8. Towards Rational and Computational MIP Design

Recent advances in computational chemistry—molecular dynamics (MD), density functional theory (DFT), and molecular docking—are increasingly informing rational MIP design. Simulations enable quantitative prediction of monomer–template binding energies, optimal stoichiometries, and interaction geometries, drastically reducing empirical trial and error [46]. Alongside these in silico methods, statistically structured design of experiments (DoE) and chemometric approaches offer an experimental route to the same goal. By simultaneously varying multiple synthesis factors—according to factorial or response-surface designs rather than one variable at a time—they quantify both the individual contribution and the interactions among polymerization method, crosslinker, functional monomer, and porogen with respect to measurable recognition responses such as the imprinting factor, the apparent affinity constant, and the number of binding sites, thereby identifying optimal formulations from a limited number of synthetic trials [47,48]. ML is emerging as a complementary tool, correlating monomer descriptors (dipole moment, pKa, hydrophobicity) with experimental binding performance and imprinting quality [13]. These digital approaches pave the way for data-driven imprinting, in which algorithms guide monomer and porogen selection; their present limitations—chiefly the scarcity of large, standardised, and comparably reported datasets—are addressed in Section 9.7 and Section 11.1.

Whether such rationally designed cavities can actually be exploited by a transducer, however, depends on where they are located within the material and on how the polymer is shaped—the subject of the following section.

## 3. MIP Architectures and Formats for Biosensing

The performance of MIPs in biosensing is intimately linked to their architectural design—that is, the way recognition sites are distributed within the polymer matrix and how the polymer interfaces with the transducer [49,50]. While the underlying chemistry of imprinting defines binding selectivity, polymer morphology, thickness, and topology determine how fast and how efficiently those recognition sites can interact with analytes in complex environments, particularly in sensor configurations requiring rapid mass transport and efficient signal transduction [50].

The evolution of MIP formats can be read as a progressive reduction in the distance between the analyte and its binding site: from ground bulk monoliths, through thin films and nanoparticles, to surface- and epitope-confined layers [49]. The most recent formats advance along two complementary lines: imprinted nanogels, soft, water-swollen networks that operate well in physiological media [30], and hybrid architectures that couple the imprinted layer to highly conductive or optically active nanomaterials (e.g., graphene, gold nanoparticles), so that the analyte not only binds rapidly but its recognition is transduced into an amplified signal [50].

### 3.1. Bulk and Monolithic MIPs: The Origin of the Field

Early MIPs were synthesized as bulk monoliths—large, highly crosslinked polymers produced by free-radical polymerization in the presence of the template molecule. After polymerization, the monolith was ground and sieved to obtain microparticles with accessible binding sites. Grinding compromised analytical performance on three counts: the cavities were heterogeneously distributed and randomly oriented, with a substantial fraction destroyed or deformed during milling; only the small proportion of sites exposed at the particle surface was kinetically accessible, with the remainder being either unusable or reachable only over long periods; and the irregular, friable particles obtained could not be produced with a reproducible site density, which is incompatible with the batch-to-batch consistency required for device manufacture.

These limitations restricted bulk MIPs mainly to SPE and chromatography, where equilibrium binding is acceptable. Nevertheless, they provided the first demonstration of synthetic molecular recognition and remain valuable for preparative separations and pre-concentration steps preceding detection [51,52]. Collectively, these shortcomings motivated the move toward the interfacial and nanostructured formats discussed in the following subsections.

### 3.2. Thin-Film and Electropolymerized MIPs

The transition from powders to thin films deposited directly on the transducer marked a decisive advance for MIP-based sensing. Beyond the kinetic gains already noted, the thin-film format is what first brings the recognition layer directly onto the transducer, enabling intimate electrical or optical coupling and in situ control of film thickness and morphology [49].

#### 3.2.1. Spin-Coated and Grafted MIP Films

Thin MIP films can be prepared by spin-coating, drop-casting, or surface grafting of pre-polymer mixtures followed by in situ polymerization. The result is a conformal coating with thickness typically ranging from tens to hundreds of nanometers, compatible with optical and piezoelectric transducers (SPR, QCM, SAW). Surface grafting approaches, such as “grafting-from” via surface-initiated radical polymerization (SIP), allow controlled growth of imprinted layers directly from functionalized substrates, improving adhesion and reproducibility [49].

#### 3.2.2. Electropolymerized MIPs

In electrochemical sensing, electropolymerization offers unique control over film thickness, morphology, and composition. Functional monomers such as aniline, pyrrole, o-phenylenediamine, and thiophene derivatives can be polymerized electrochemically in the presence of the target molecule, which acts as a molecular template. Upon potential cycling, thin, conductive MIP films form on electrode surfaces, allowing precise tuning of film thickness (10–200 nm) and immediate electrical coupling to the transducer. Electropolymerized MIPs are particularly suitable for amperometric, voltammetric, and impedimetric biosensors, combining high reproducibility with scalability on screen-printed and pencil-graphite electrodes, and have reached picomolar detection of clinical analytes in plasma [50,53].

### 3.3. MIP Nanoparticles and Nanogels

Nanostructured MIPs—nanoparticles, nanogels, and nanocapsules—represent a breakthrough toward faster kinetics, higher surface area, and easier processability, with particle diameters typically in the 20–500 nm range, which makes them readily dispersible in solution and easy to immobilize, with a very high surface-to-volume ratio [54]. Core–shell configurations, in which a thin imprinted polymer layer is confined to the surface of a nanoparticle core (e.g., magnetic Fe_3_O_4_), combine MIP recognition specificity with functional properties such as magnetic separation and fluorescence modulation, enabling highly sensitive biosensing in complex media [54]. MIP nanogels, exhibiting soft, swollen networks with high water content, provide rapid diffusion and biocompatibility for protein and biomarker sensing and can be engineered to respond reversibly to environmental stimuli (pH, temperature, light) [55].

### 3.4. Surface Imprinting and Interfacial Design

Recognition of large biological targets, however, depends not only on particle size but also on where the cavities sit relative to the polymer–solution interface.

For biosensing applications—particularly those involving large biomolecules such as proteins, viruses, or cells—surface imprinting is the most effective strategy [49,56,57]: it resolves the mass-transport limitation identified in Section 2.7 by confining the recognition sites to a thin interfacial layer, so that cavity accessibility is no longer governed by intraparticle diffusion.

Surface-imprinted MIPs are typically fabricated using approaches that enable precise control over the interfacial architecture. These include soft lithography or microcontact imprinting, in which a template-bearing layer is brought into contact with a monomer film prior to polymerization; sol–gel imprinting, leading to inorganic–organic hybrid films with molecularly defined surface cavities; and plasma-assisted or photochemical imprinting strategies that allow micro- and nano-patterning of recognition surfaces [49,56].

This matters most for protein and cell imprinting, where the template size (from tens of nanometers up to micrometers for whole cells) would simply exceed the diffusion limits of a bulk network [49,56]. By mimicking key features of biological interfaces, such as cell membranes, surface-imprinted polymers can thus function as fully synthetic analogues of natural recognition layers, enabling label-free detection of entire organisms and complex biological targets in biosensing platforms [49].

Beyond architectural confinement, further advances in macromolecular recognition require control over the molecular information encoded within the binding sites, particularly when targeting structurally complex proteins.

### 3.5. Epitope Imprinting for Macromolecular Recognition

Full-protein imprinting is often hindered by protein denaturation, steric constraints, and inefficient template removal, particularly when imprinting is performed in aqueous or biologically relevant environments [30,31]. To overcome these limitations, epitope imprinting has emerged as an effective alternative strategy, in which a short and structurally accessible peptide fragment—corresponding to the recognition epitope of the target protein—is employed as the template rather than the full macromolecule [30,31].

By restricting imprinting to a well-defined peptide sequence, epitope imprinting enables controlled orientation and improved accessibility of binding sites, while significantly facilitating template removal after polymerization [30,31]. As a result, the imprinted cavities retain high fidelity toward the parent protein, despite being generated from a reduced molecular fragment. This approach has been successfully applied to clinically relevant biomarkers and other large biological targets—including the aqueous human chorionic gonadotropin example detailed in Section 2.5 (Figure 4)—with binding affinities and selectivities approaching those of antibody–antigen systems [30,31,57].

Beyond binding performance, epitope imprinting bridges molecular imprinting and proteomics by enabling rational selection of exposed, functionally relevant peptide sequences as templates—thereby decoupling recognition from the structural complexity of full-length proteins and offering a scalable route to imprinting large biomacromolecules for biosensing in complex media [30,31].

### 3.6. Composite and Hybrid MIP Architectures

The combination of MIPs with nanomaterials and functional substrates has dramatically expanded the design landscape of MIP-based biosensors [50,58]. By integrating complementary material functionalities into a single recognition layer, hybrid MIP architectures overcome intrinsic limitations of pristine polymers in terms of signal transduction, sensitivity, and response time.

Hybrid MIPs combine the imprinted network with three functional classes of inclusion: conductive nanomaterials that enhance electrical transduction and charge transport toward the electrode; metal or semiconductor nanoparticles that add optical, plasmonic, or catalytic functionality; and porous scaffolds or metal–organic frameworks that increase the surface area and improve analyte diffusion toward the imprinted sites [50,58,59]. The specific filler chemistries are surveyed in Section 4.5, and their quantitative effect on electrochemical signal amplification in Section 5.5; the emphasis here is on the architectural consequence, namely that recognition and transduction can be co-located within a single interfacial layer.

**Figure 6:**
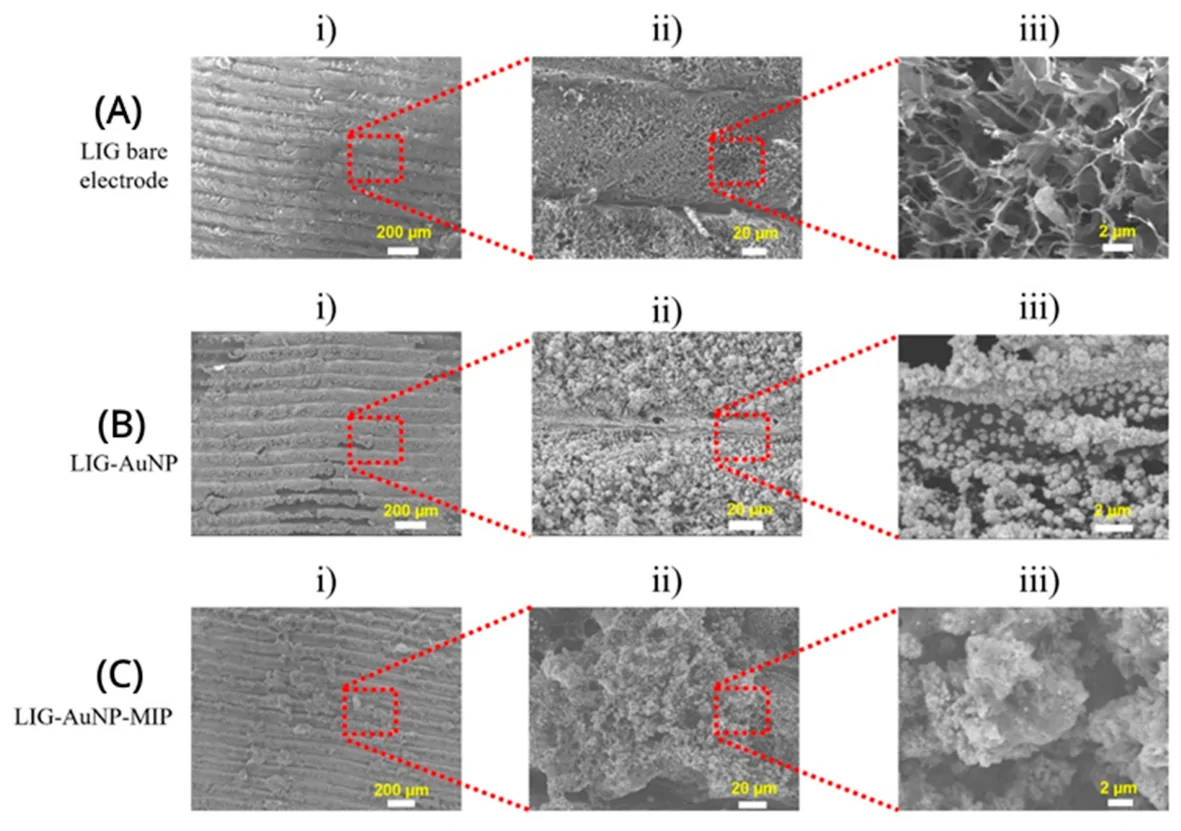
Build-up of a hybrid, carbon-supported molecularly imprinted electrode: SEM micrographs of (**i**) the bare laser-induced graphene (LIG) scaffold, (**ii**) the LIG decorated with electrodeposited gold nanoparticles (LIG/AuNPs), and (**iii**) the final electrode after electropolymerization of the PEDOT-based imprinted layer (LIG/AuNPs/MIP). The conductive LIG/AuNP support enhances electron transfer while the imprinted polymer supplies molecular selectivity. Reproduced from Animashaun et al. [50], Biosensors 2025, 15, 384, under CC BY 4.0.

These composite systems exploit synergistic effects: nanomaterials provide high conductivity and/or optical field enhancement, while the MIP matrix supplies molecular selectivity and chemical robustness. For example, carbon-supported MIP electrodes enhanced with nanostructured components have been shown to yield faster response times and improved sensitivity in electrochemical sensing platforms due to improved electron transfer and reduced diffusion resistance [50]. The same composite approach extends across target classes, from small molecules to protein biomarkers and, further, to viral and cellular targets (Section 7.4). Similarly, plasmonic nanoparticle–MIP composites amplify the optical response in SPR detection, a mechanism whose chemistry and transduction are developed in Section 4.5 and Section 6.1 [58]. A representative example is shown in Figure 6, where a laser-induced-graphene scaffold decorated with gold nanoparticles supports an electropolymerized imprinted layer.

Beyond single-mode operation, hybridization also facilitates multi-modal sensing strategies, in which electrochemical and optical signals can be generated from the same (or co-integrated) imprinted recognition layer, supporting an emerging trend toward multi-analyte and multi-signal biosensors that combine selective recognition with versatile transduction mechanisms [50,58].

### 3.7. Water-Compatible and Stimuli-Responsive Architectures

For operation in biological environments, MIPs must function reliably in aqueous and chemically complex media. To this end, water-compatible MIPs are commonly designed using hydrophilic monomers and crosslinkers, often combined with antifouling or zwitterionic components that suppress the non-specific adsorption which would otherwise mask the imprinted sites in physiological media [60]. These strategies are the materials-level counterpart of the aqueous-imprinting problem set out in Section 2.5; the specific monomer and crosslinker chemistries involved are surveyed in Section 4.1.3 and Section 4.2.2.

Beyond simple aqueous compatibility, an emerging class of MIPs incorporates stimuli-responsive elements that enable reversible modulation of molecular recognition [61]. In these systems, external triggers—such as temperature, pH, electrical potential, or light—induce controlled changes in polymer conformation, polarity, or swelling state, thereby switching binding interactions on or off. Thermoresponsive architectures, for instance, exploit temperature-dependent volume transitions to regulate analyte access to imprinted cavities, while electrically addressable MIPs allow dynamic control through the applied potential, which is particularly useful for active regeneration in electrochemical formats: a suitable potential pulse promotes release of the bound target, restoring the baseline and making the sensor available for a further measurement (Section 5.6).

Although photoresponsive and multi-stimuli-responsive MIPs have also been reported, their practical implementation in biosensing remains more limited and system-dependent, owing to challenges in stability, penetration depth, and compatibility with aqueous media.

Overall, water-compatible and stimuli-responsive MIPs progressively erase the distinction between passive recognition and active functional materials, moving toward adaptive sensing interfaces that emulate the responsiveness of biological systems and provide controllable, reversible, context-dependent recognition for next-generation biosensors in complex environments.

### 3.8. From Materials to Device Integration

**Figure 7:**
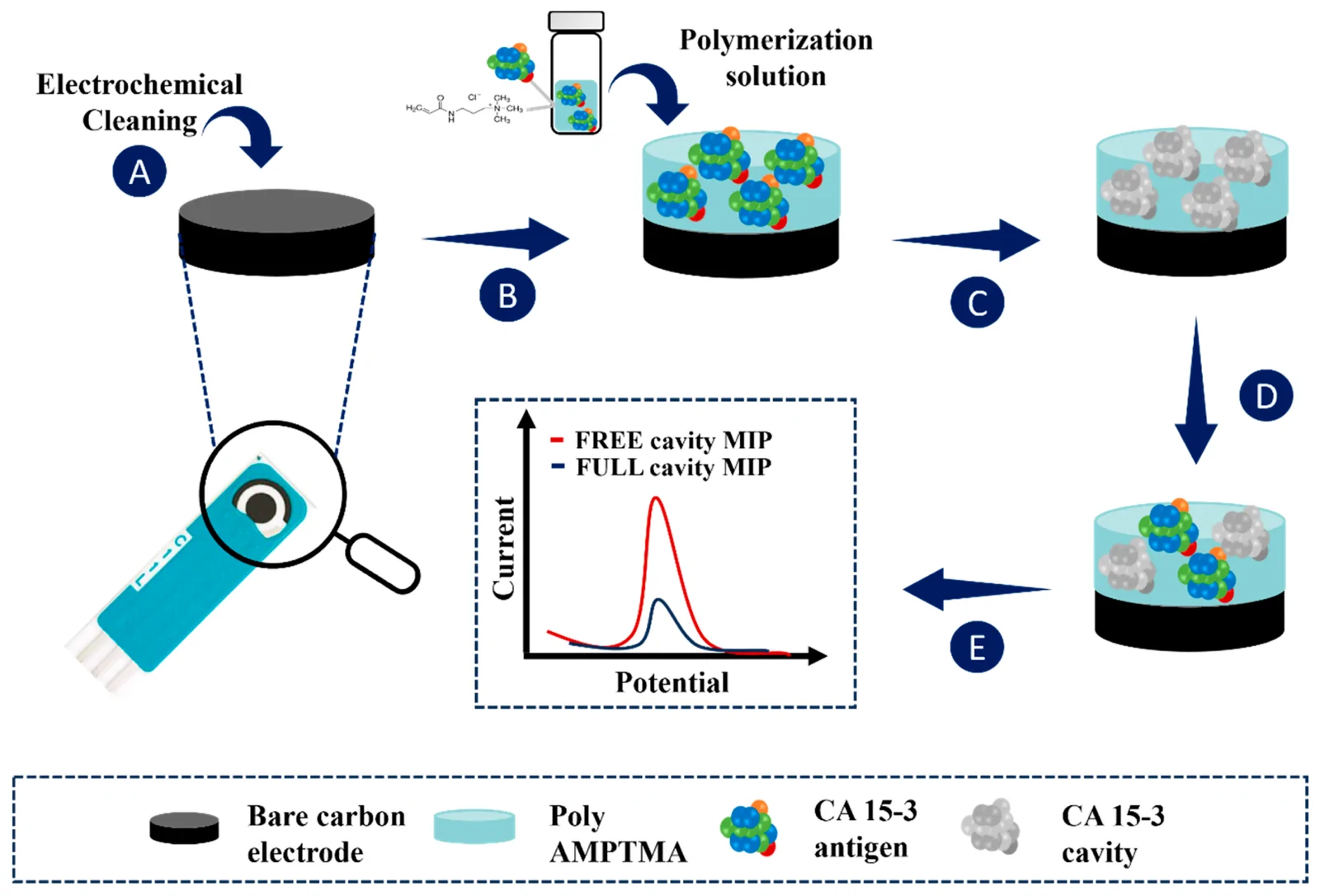
Assembly of an electropolymerized molecularly imprinted polymer biosensor on a disposable carbon screen-printed electrode for point-of-care detection of the breast-cancer biomarker CA 15-3: electrochemical pre-treatment, electropolymerization of the functional monomer in the presence of the CA 15-3 template, template extraction to unveil the recognition cavities, and selective rebinding for electrochemical read-out. (**A**) work electrode pre-treatment; (**B**) ELP of a solution containing CA 15-3 protein and monomer (AMPTMA); (**C**) CA 15-3 protein removal from polymer matrix; (**D**) template binding on the MIP surface; (**E**) analytical performance of the sensor. Reproduced from Oliveira et al. [62], RSC Adv. 2024, 14, 15347–15357, under CC BY 4.0.

The architectural flexibility of MIPs enables seamless integration with a wide range of transduction platforms, allowing material design to be directly aligned with device-level performance requirements [30,62]. Thin films and electropolymerized layers are particularly well suited for electrochemical and mass-sensitive sensors, as they provide intimate coupling between the imprinted recognition layer and the electrode surface, enabling efficient charge transfer, rapid response, and straightforward regeneration [62]. In this context, electropolymerized MIP films grown in situ on screen-printed or carbon-based electrodes have demonstrated high selectivity and operational robustness for clinically relevant biomarkers, supporting their translation toward point-of-care sensing devices [62]. Such a screen-printed, electropolymerized MIP biosensor for the breast-cancer marker CA 15-3 is illustrated in Figure 7.

In parallel, the nanoparticle and nanogel formats described in Section 3.3 are particularly well suited to optical and mass-sensitive detection: their aqueous dispersibility and compatibility with surface immobilisation make them effective in label-free SPR and QCM platforms, and, when combined with surface or epitope imprinting, they extend selective recognition to proteins and cells in complex media [30].

Overall, by matching MIP morphology and architecture to the chosen detection principle—electrical, optical, acoustic, or mechanical—researchers can rationally tailor biosensor response dynamics, detection limits, and long-term stability. This convergence of materials engineering and device integration positions MIPs as engineerable recognition elements at the intersection of materials science, biointerfaces, and device design [30,62], provided that the underlying polymer chemistry is selected accordingly, which is the subject of the next section.

## 4. Polymer Chemistry and Functional Monomers for Biosensing MIP

The core of any molecularly imprinted polymer (MIP) lies in its chemical formulation—the judicious combination of functional monomers, crosslinkers, and porogenic media that define not only the strength and selectivity of template binding but also the physicochemical properties essential for biosensing. Whereas Section 2 and Section 3 addressed, respectively, the physical basis of recognition and the geometry in which it is deployed, this section examines the chemical inventory itself: how the interplay between monomer functionality, crosslink density, and solvation environment determines the fidelity and accessibility of imprinted cavities.

In biosensing, this chemical inventory must ultimately be balanced against three device-level constraints—binding affinity, diffusion kinetics, and transduction compatibility, often under aqueous or physiological conditions—a balance made quantitative in Section 4.7.

### 4.1. Functional Monomers: The Foundation of Selective Recognition

The functional monomer defines the chemical identity of the MIP: it governs how the template is pre-organized, which interactions stabilize the complex, and how recognition proceeds upon rebinding.

#### 4.1.1. Classical Non-Covalent Monomers

The earliest and most widely used functional monomers for MIPs are small acrylic or vinyl compounds capable of hydrogen bonding or electrostatic interactions. Among them, MAA remains the most widely used, forming strong H-bonding networks with amine, hydroxyl, or carbonyl groups and often emerging as the optimal choice during monomer/porogen screening for non-covalent imprinting systems [63]. Other commonly employed monomers include AAm and N-isopropylacrylamide (NIPAm), which are frequently adopted to enhance hydrophilicity and/or introduce thermo-responsiveness, enabling imprinting and rebinding under conditions closer to biological media [64]. 4-VP is widely used to introduce basic nitrogen donor sites and strengthen interactions with acidic or electron-poor templates, including electrochemical MIP configurations where the binding layer is integrated directly on the transducer surface [65]. In parallel, aromatic vinyl monomers (e.g., styrene) are exploited to promote π–π and hydrophobic interactions with aromatic targets; comparative studies explicitly show how the balance between hydrogen bonding (e.g., MAA) and π–π/hydrophobic contributions (e.g., styrene) can dictate imprinting fidelity and rebinding performance [66]. Finally, urea-/thiourea-based monomers provide multiple, directional hydrogen-bond donors and have been implemented as functional motifs within MIP architectures to strengthen recognition of polar functionalities (e.g., carboxylates) in particle- and probe-type formats [67]. Overall, the diversity of these monomers enables fine-tuning of recognition sites toward analytes of varying polarity, size, and functionality; however, their efficacy remains strongly coupled to solvent choice and crosslinking degree, which jointly control pre-complex stability and the ultimate accessibility of binding sites. The same non-covalent chemistry also underpins the fluorescent surface-imprinted formats discussed in Section 6.2 [68].

#### 4.1.2. Monomers for Covalent and Semi-Covalent Imprinting

For imprinting strategies involving reversible covalent bonding, specialized monomers provide defined binding stoichiometries. Typical examples include boronic acid derivatives for targets bearing vicinal diols (e.g., carbohydrates and aminoglycosides), where reversible boronate-ester formation can be exploited to enhance selectivity and binding efficiency in complex matrices, as demonstrated both for aminoglycosides and for cis-diol-bearing biomacromolecular targets [69,70]. In addition, carbonyl-bearing vinyl monomers (aldehyde- or ketone-functional) and related dynamic covalent motifs enable Schiff-base/imine chemistry with amine-containing templates, providing more uniform site formation and facilitating controlled template release under appropriate conditions [71]. Although less general than non-covalent imprinting, these covalent/semi-covalent systems can yield more homogeneous binding sites, with improved selectivity and more predictable binding/rebinding behavior, which is advantageous for small-molecule biosensing and for imprinting workflows where site fidelity and robustness are prioritized [69,70,71].

#### 4.1.3. Charged and Zwitterionic Monomers

To extend MIP functionality to aqueous and biological environments, ionic and zwitterionic monomers have become increasingly important. Quaternary-ammonium monomers (e.g., trimethylammonium-bearing acrylamides/methacrylates) enable the introduction of cationic sites, strengthening electrostatic pre-organization toward anionic or highly polar templates and supporting robust recognition in complex matrices such as serum when used in surface-confined or electropolymerized formats [62]. Conversely, anionic monomers such as acrylic acid (AA) provide negatively charged functionalities and strong hydrogen-bonding capability, which can stabilize pre-complexes with protonatable/cationic analytes and guide rational monomer selection in polar media [72].

Zwitterionic materials such as sulfobetaines and carboxybetaines suppress non-specific protein adsorption through strong surface hydration; this is the chemical basis of the antifouling interfaces invoked in Section 3.7 and Section 8.6, and Section 9.5, and an essential prerequisite for reliable measurement in complex biofluids. In particular, sulfobetaine-based networks have been successfully combined with conductive components to realize water-compatible, anti-fouling MIP sensing interfaces operating in real biofluids (e.g., sweat) [73]. It should be stressed that this antifouling action mitigates matrix effects by suppressing non-specific adsorption at the interface, and is therefore distinct from—and does not by itself improve—the intrinsic fidelity of the imprinted cavities: zwitterionic chemistry preserves the apparent selectivity in complex media by lowering background binding, rather than by enhancing template–cavity complementarity.

### 4.2. Crosslinkers: Stabilizing and Structuring the Recognition Matrix

Building on the rigidity–accessibility trade-off established in Section 2.3, this subsection shifts the focus from crosslinker content to crosslinker identity, comparing the specific chemistries used to fix the monomer–template pre-assembly and to tune network permeability and elasticity.

#### 4.2.1. Conventional Crosslinkers

EGDMA is among the most widely used crosslinkers in MIP synthesis, offering a practical balance between reactivity and network rigidity; comparative studies explicitly show that changing the crosslinker (e.g., EGDMA vs. TRIM) can markedly affect binding capacity and imprinting performance in particle-based MIPs [48,74]. TRIM, due to its higher functionality, is frequently selected when increased crosslink density and improved structural stability are desired; crosslinker screening experiments report TRIM-based formulations providing enhanced adsorption efficiency and stability relative to EGDMA, consistent with a denser network [75]. DVB introduces an aromatic, hydrophobic crosslinked scaffold and is often preferred in systems targeting hydrophobic/aromatic environments; experimental comparisons of DVB-based networks against alternative crosslinkers confirm that crosslinker identity can substantially tune sorption performance and the rigidity/accessibility of binding domains [76]. These comparisons make the rigidity–accessibility trade-off introduced in Section 2.3 chemically explicit: crosslinker identity, and not only crosslinker content, determines where the optimum between site fidelity and transport kinetics lies in sensor-oriented formulations [74,75,76].

#### 4.2.2. Flexible and Hydrophilic Crosslinkers

For aqueous imprinting and mechanically compliant sensing layers, hydrophilic crosslinkers such as N,N′-methylenebisacrylamide (MBAA) and poly(ethylene glycol) diacrylate (PEGDA) are widely adopted to introduce water compatibility while preserving sufficient network integrity for stable rebinding. In hydrogel-formulated MIPs, these crosslinkers enable hydrated matrices that support diffusion-driven recognition under mild conditions and reduce cracking or brittleness compared to highly rigid, hydrophobic networks, which is particularly relevant when MIPs are coupled to soft or deformable substrates. Recent examples include MBAA-crosslinked MIP hydrogels prepared in water and processed as porous, functional networks for selective uptake/removal of target molecules, confirming the suitability of MBAA for robust hydrophilic imprinting [77], as well as PEGDA-based molecularly imprinted macroporous hydrogels designed for operation in wet biological environments, highlighting PEGDA as an effective hydrophilic crosslinker in imprinting architectures intended for biointerfaces [78].

**Figure 8:**
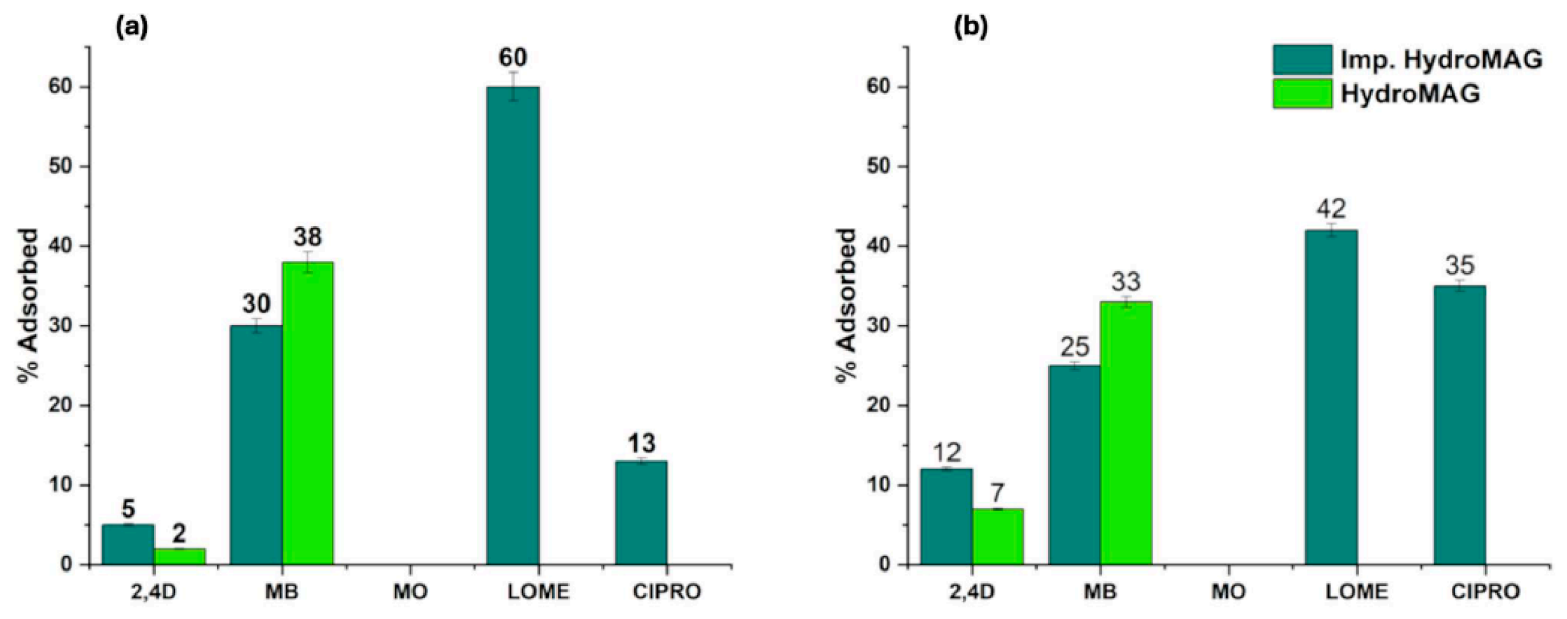
(**a**,**b**) Adsorption performances of HydroMAG and Imp.HydroMAG imprinted with (**a**) Lomefloxacin and (**b**) Ciprofloxacin towards targets (Lome and Cipro, respectively) and different non-target contaminants (2,4-D, MB, and MO), showing preferential uptake of the template antibiotics (lomefloxacin and ciprofloxacin) over non-target contaminants (2,4-D, methylene blue, methyl orange). The marked imprinting effect confirms the suitability of the MBAA-crosslinked hydrophilic network for robust aqueous molecular recognition. Adapted from Curcuruto et al. [77], Polymers 2024, published under CC BY 4.0.

A representative example of this hydrophilic imprinting strategy is shown in Figure 8, in which an MBAA-crosslinked HEMA hydrogel retains a clear-cut molecular selectivity for its template antibiotic over structurally unrelated contaminants, illustrating how a water-compatible crosslinked network can preserve robust recognition in fully aqueous media [77].

### 4.3. Solvents and Porogens: Shaping the Microstructure

The porogenic solvent defines the microenvironment in which imprinting occurs, influencing pore size, surface area, and site accessibility [25]. Aprotic organic porogens such as acetonitrile—polar yet non-hydrogen-bond-donating—favour strong template–monomer interactions and well-defined cavities by minimising competition during pre-assembly, although they typically yield hydrophobic networks [25]. Recent innovations include ionic liquid-based imprinting, where ILs act simultaneously as solvent and functional component, stabilizing polar interactions and enhancing electrochemical transduction [79], as well as deep eutectic solvents (DES) employed as green porogens with tunable polarity [25]. In parallel, emulsion and solvent-free imprinting strategies have emerged to minimize environmental impact while enabling nanostructured MIPs with improved mass transport [45].

### 4.4. Copolymer Systems and Monomer Combinations

In many high-performance MIPs, binary or ternary monomer systems are deliberately adopted to generate synergistic recognition environments, where different functional comonomers contribute complementary interaction motifs (e.g., H-bonding, electrostatic pairing, and π–π contributions) within the same cavity microenvironment. For instance, pairing an acidic monomer (e.g., methacrylic acid, MAA) with a basic monomer (e.g., 4-vinylpyridine, 4-VP) provides a broader interaction palette and can enhance binding robustness toward ionizable analytes by stabilizing multiple competing binding geometries during pre-assembly and rebinding [80,81]. This benefit is not automatic: the same acid–base complementarity can drive direct MAA–4-VP self-association, which competes with template complexation and may lower the effective availability of both monomers toward the target, so that the net outcome depends on the relative monomer–monomer versus monomer–template affinities and on the chosen stoichiometry. Similarly, AAm/HEMA co-monomer formulations are frequently used to balance hydrophilicity and mechanical integrity, enabling strong hydrogen-bond networks while maintaining a polymer microenvironment compatible with polar targets and sensor fabrication constraints [82]. Overall, this co-monomer tuning provides a practical handle to modulate binding-site polarity, rigidity, and the local dielectric environment, with direct consequences on sensitivity and selectivity in device-relevant matrices [81,82].

### 4.5. Nanocomposite and Conductive MIP Matrices

Building on the composite architectures introduced in Section 3.6, this subsection considers the chemical identity of the filler phases and the specific functionality—electronic, optical, or magnetic—that each contributes to the imprinted matrix. Carbon-based nanomaterials, including graphene-derived electrodes, provide high conductivity and large effective surface area, enabling efficient electropolymerized MIP layers for rapid, non-invasive electrochemical sensing (e.g., cortisol on laser-engraved graphene) [83]. Metal and semiconductor nanoparticles (e.g., AuNPs and ZnO) can amplify electrochemical signals and improve catalytic/charge-transfer pathways when embedded beneath or within imprinted layers, enabling sensitive determination of pharmaceuticals and related analytes [84], while AuNP-decorated plasmonic MIP interfaces have been demonstrated to enhance SPR readout in imprinting-based recognition of endocrine disruptors such as bisphenol A [58]. Magnetic nanoparticles (e.g., Fe_3_O_4_) enable straightforward target enrichment and pre-concentration and can be combined with MIP coatings to yield electrochemical platforms for analyte determination in real water matrices [85]. Beyond classical fillers, MXene-based conductive scaffolds have been used to host electropolymerized MIP recognition layers, supporting sensitive and selective detection in serum-relevant conditions [86]. Finally, MOF–MXene hybrid interfaces provide structural tunability and high interfacial activity; ratiometric MIP electrochemical architectures integrating MOFs and MXenes have been reported to improve signal robustness and anti-interference performance in complex matrices [87]. A more recent frontier pushes the filling phase toward atomically dispersed active sites. Single-atom catalysts (SACs), i.e., isolated metal atoms (e.g., Pt, Ru) anchored on nitrogen-doped carbon, MXene, or hollow carbon supports, combine near-unity atom utilization with tunable electronic structure and have begun to be incorporated below or within printed layers to intensify interfacial charge transfer and electrocatalytic turnover: reported examples include single-atom Pt/MXene interfaces for protein recognition [88], Fe_3_O_4_–Ru_1_@N-doped carbon platforms for small-molecule sensing [89], and single-atom Pt architectures on paper for phenolic pollutants [90]. Sub-nanometric metal nanoclusters occupy an intermediate regime between single atoms and plasmonic nanoparticles; gold nanoclusters have been coupled to printed films both as conductivity-enhancing scaffolds in electrochemical formats [91] and as intrinsic photoluminescent reporters in fluorescent ones [92]. Because the benefit of these ultradispersed phases is proportional to site accessibility and is compromised by atom aggregation or leaching during repeated cycles, their operational durability in real matrices should be reported explicitly rather than taken for granted [93]. In electroactive MIPs, conductive fillers thus transform the otherwise insulating polymer layer into an electrically conductive composite, enabling efficient charge transduction and real-time sensing while preserving molecular selectivity; the term semiconducting should be reserved for systems in which a defined band gap and semiconductor-like transport have actually been demonstrated (e.g., photoactive metal-oxide phases) [83,84,86,87].

### 4.6. Biocompatible and “Green” Monomers for Sustainable MIP Design

Monomer and porogen selection is increasingly constrained by sustainability considerations as well; the following paragraphs are confined to the chemical options available, while the wider green-design and life-cycle framework is developed in Section 10. Monomers derived from natural or renewable sources—such as itaconic acid and polysaccharide-derived matrices—provide eco-friendly alternatives to petrochemical acrylates and can be selected to improve water-compatibility and reduce the environmental footprint of MIP preparation [94,95]. In parallel, water-based hydrogel formulations and additive-free processing routes (e.g., emulsion-free fabrication) contribute to minimizing the use of toxic organic porogens while maintaining accessible, porous recognition architectures [78,96]. In this context, room-temperature ionic liquid (RTIL) imprinting has also emerged as a solvent-sparing strategy, enabling electrosynthesis of MIP films in microliter-scale volumes while replacing conventional volatile organic solvents [97]. Beyond their environmental profile, such materials extend MIP use to biointerfaces and implantable formats, where cytocompatibility and degradation behaviour become design parameters in their own right [78] (Section 10.2 and Section 10.5).

### 4.7. Correlating Polymer Composition with Biosensing Performance

The relationship between polymer composition and biosensor metrics can be summarized across three interdependent parameters: affinity and selectivity—dictated by the strength and geometry of monomer–template interactions; signal transduction efficiency—governed by the polymer’s electrical/optical properties and interfacial contact with the electrode or waveguide; and environmental compatibility—defined by hydrophilicity, fouling resistance, and mechanical stability. For example, the use of tailored functional monomers with optimized binding energies has been shown to improve both selectivity and sensitivity in MIP-based potentiometric sensors relative to conventional monomers, evidencing how monomer choice influences affinity and selectivity at the molecular level [98]. The integration of conductive nanostructured interfaces (e.g., graphene oxide) with electropolymerized MIP films significantly enhances signal transduction efficiency and enables well-defined polymerization around the template, directly improving electrochemical performance metrics in protein sensing applications [99]. Moreover, controlling the polymer thickness and morphology by adjusting electropolymerization parameters (e.g., number of cycles, solvent composition) directly affects polymer porosity and rebinding kinetics, illustrating how environmental compatibility (e.g., diffusion rates and accessible binding sites) modulates overall sensor response [99].

**Figure 9:**
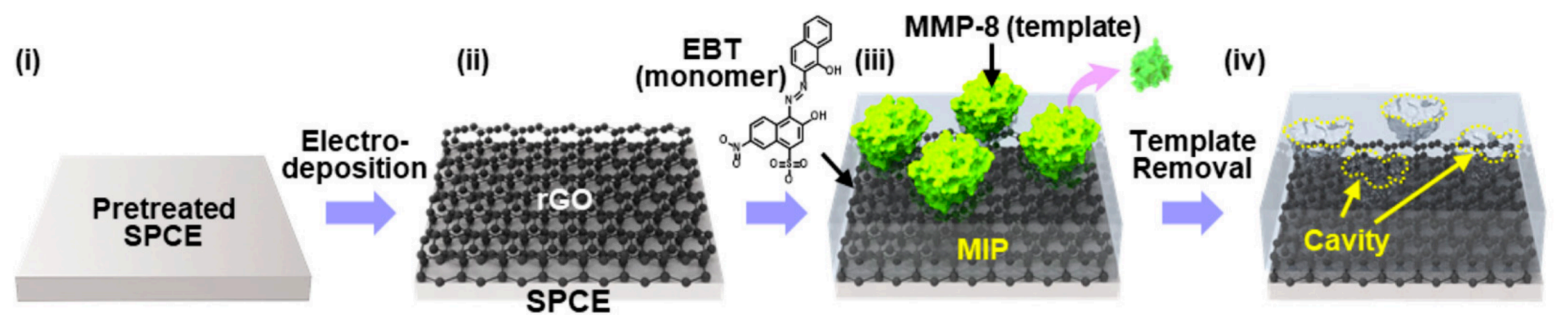
Schematic of the fabrication of a conductive, electropolymerized MIP electrode: (**i**) pretreatment of the screen-printed carbon electrode (SPCE); (**ii**) electrodeposition of the graphene oxide (GO) interlayer; (**iii**) electropolymerization of the poly(eriochrome black T) MIP film in the presence of the protein template (MMP-8); and (**iv**) template extraction to generate the recognition cavities. Adapted from Lee et al. [99], Biosensors 2025, published under CC BY 4.0.

This rational, multilayer design is exemplified in Figure 9, which depicts how a graphene-oxide interlayer and an electropolymerized MIP film are sequentially assembled on the electrode and subsequently subjected to template extraction, directly connecting the electropolymerization conditions to the formation of accessible, transduction-ready recognition cavities [99].

These examples underscore that multi-objective polymer design—balancing molecular recognition, electrical interfacing, and material compliance—is essential for high-performance MIP biosensors. How the resulting recognition event is converted into an analytical signal is examined in the following two sections, beginning with electrochemical transduction.

## 5. Transduction Strategies I: Electrochemical MIP-Based Biosensors

Electrochemical transduction is the most widely adopted platform for molecularly imprinted polymer (MIP)-based biosensors, for a reason already apparent from Section 3.2.2: the recognition layer can be grown directly on the transducer, so that the imprinted cavities and the electron-transfer interface coincide. In these systems, molecular recognition events at the polymer–electrode interface are translated into measurable electrical signals—changes in current, potential, impedance, or capacitance—enabling real-time, label-free detection of analytes from small molecules to macromolecular biomarkers. For example, electropolymerized MIP films on disposable carbon electrodes have demonstrated very low detection limits and high selectivity via ferrocyanide/ferricyanide mediated voltammetry, illustrating how the analytical signal arises from the coupling between binding events and interfacial electron transfer rather than from binding affinity alone [100].

### 5.1. Fundamentals of Electrochemical Transduction in MIP Sensors

At the heart of electrochemical MIP biosensing lies the modulation of charge transfer resistance and interfacial electron kinetics upon template rebinding, which can be probed by cyclic voltammetry (CV), differential pulse voltammetry (DPV), and electrochemical impedance spectroscopy (EIS) [100,101]. Distinct transduction pathways are exploited: faradaic processes occur through redox mediators at the electrode/MIP interface; non-faradaic (capacitive) processes reflect changes in double-layer capacitance upon analyte binding; and conductivity modulation is achieved when the polymer matrix incorporates conductive fillers or intrinsically conductive monomers, allowing direct electron flow across the recognition layer [102]. Each mechanism confers different advantages in terms of analytical performance, signal gain, and environmental compatibility.

### 5.2. Amperometric and Voltammetric MIP Sensors

Amperometric and voltammetric molecularly imprinted polymer (MIP) sensors detect variations in current or potential arising from redox processes that are modulated by analyte binding at the polymer–electrode interface [100,103].

#### 5.2.1. Mechanism

In amperometric and voltammetric MIP sensors, rebinding of the target molecule within the imprinted cavities alters interfacial electron transfer in several ways. The bound analyte can sterically hinder the diffusion of redox probes toward the electrode surface, modify the local electric field and surface potential, or change the permeability of the polymer film, ultimately affecting the magnitude and position of voltammetric peaks [100]. As a result, the analytical response is typically quantified as the difference in peak current (ΔI_p_) or peak potential (ΔE_p_) between the MIP-modified electrode and its NIP counterpart, providing a direct measure of selective recognition [100,103].

#### 5.2.2. Common Electrode Systems

Electropolymerized MIP films deposited on glassy carbon electrodes (GCE), gold electrodes, or screen-printed carbon electrodes (SPCE) represent the most widely used configurations for amperometric and voltammetric sensing. Conductive monomers such as pyrrole or aniline yield intrinsically electroactive MIP layers, enabling direct signal transduction without external mediators, whereas insulating acrylate-based MIPs rely on redox probes in solution to report binding events, a conducting-versus-non-conducting design trade-off examined in detail in a dedicated review [100,103,104].

Sensitivity and detection limits can be further improved by incorporating conductive nanomaterials into the electrode—a signal-amplification strategy examined in detail in Section 5.5 [103].

#### 5.2.3. Analytical Signatures

**Figure 10:**
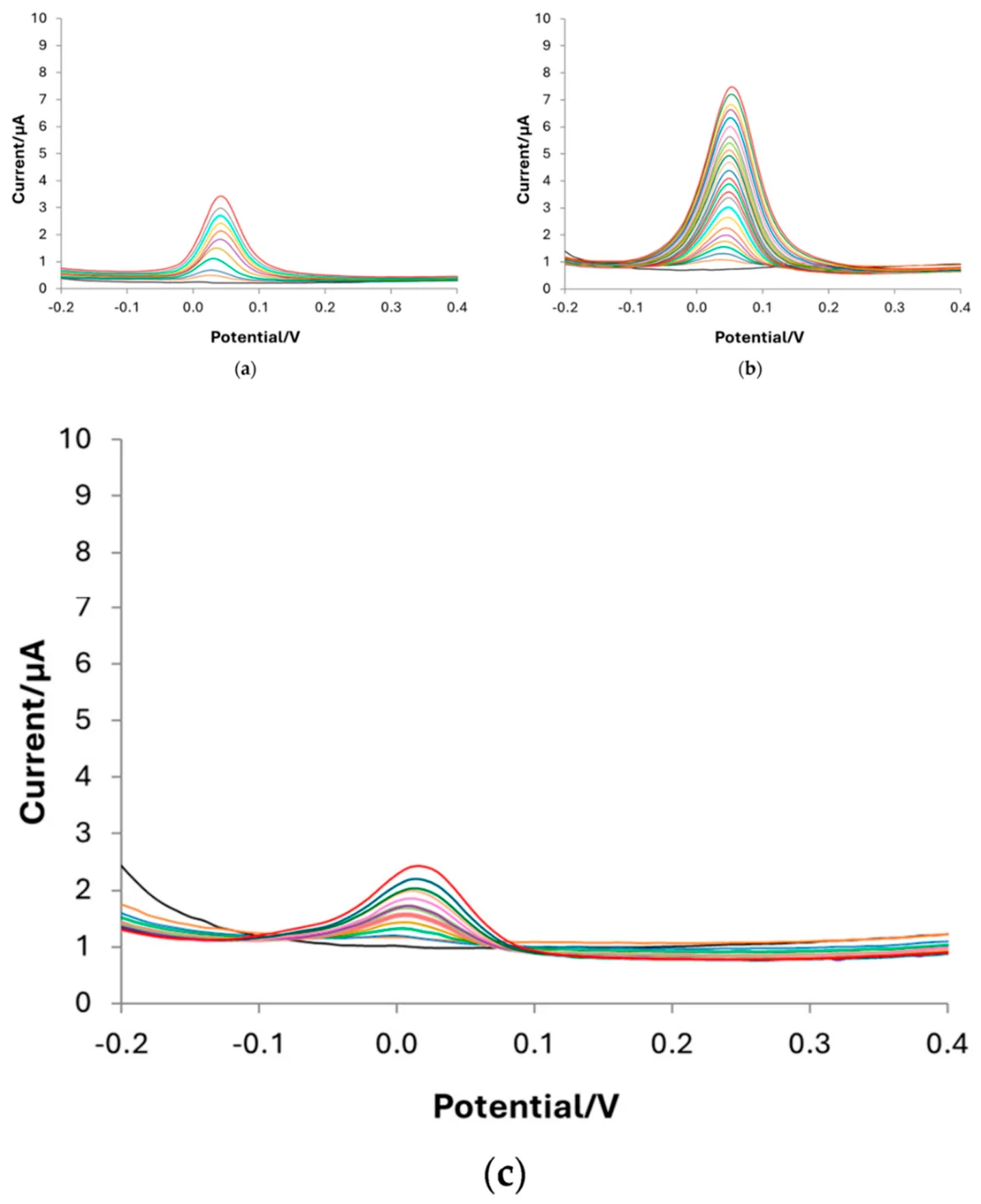
Differential pulse voltammograms recorded in 0.1 M PBS (pH 7) for increasing dopamine concentrations (0–180 µM) at (**a**) a bare screen-printed electrode, (**b**) the electropolymerised molecularly imprinted polypyrrole electrode (e-MIP), and (**c**) the non-imprinted control (e-NIP). The e-MIP electrode shows the highest sensitivity, confirming that the imprinted cavities dominate the voltammetric response. Reproduced from Merli et al. [103], Polymers 2024, under CC BY 4.0.

DPV and square-wave voltammetry (SWV) are the preferred techniques for quantitative analysis due to their high signal-to-noise ratio and sensitivity, while CV is commonly used for qualitative assessment of MIP film formation, redox probe accessibility, and binding-induced changes at the interface [100,103]. Key analytical parameters—including sensitivity, linear dynamic range, and limit of detection—can be systematically tuned by controlling film thickness, porosity, and polymerization conditions, highlighting the close interplay between MIP architecture and voltammetric performance. A representative voltammetric read-out is shown in Figure 10, where DPV on an electropolymerised polypyrrole MIP electrode yields a concentration-dependent peak-current response markedly larger than that of the corresponding non-imprinted (NIP) and bare electrodes, illustrating how the imprinted cavities govern the analytical signal [103].

### 5.3. Impedimetric and Capacitive MIP Sensors

EIS has emerged as a leading technique for label-free MIP-based biosensing, owing to its high sensitivity to interfacial phenomena and its compatibility with a wide range of polymer chemistries and electrode substrates [105,106].

#### 5.3.1. Principle

**Figure 11:**
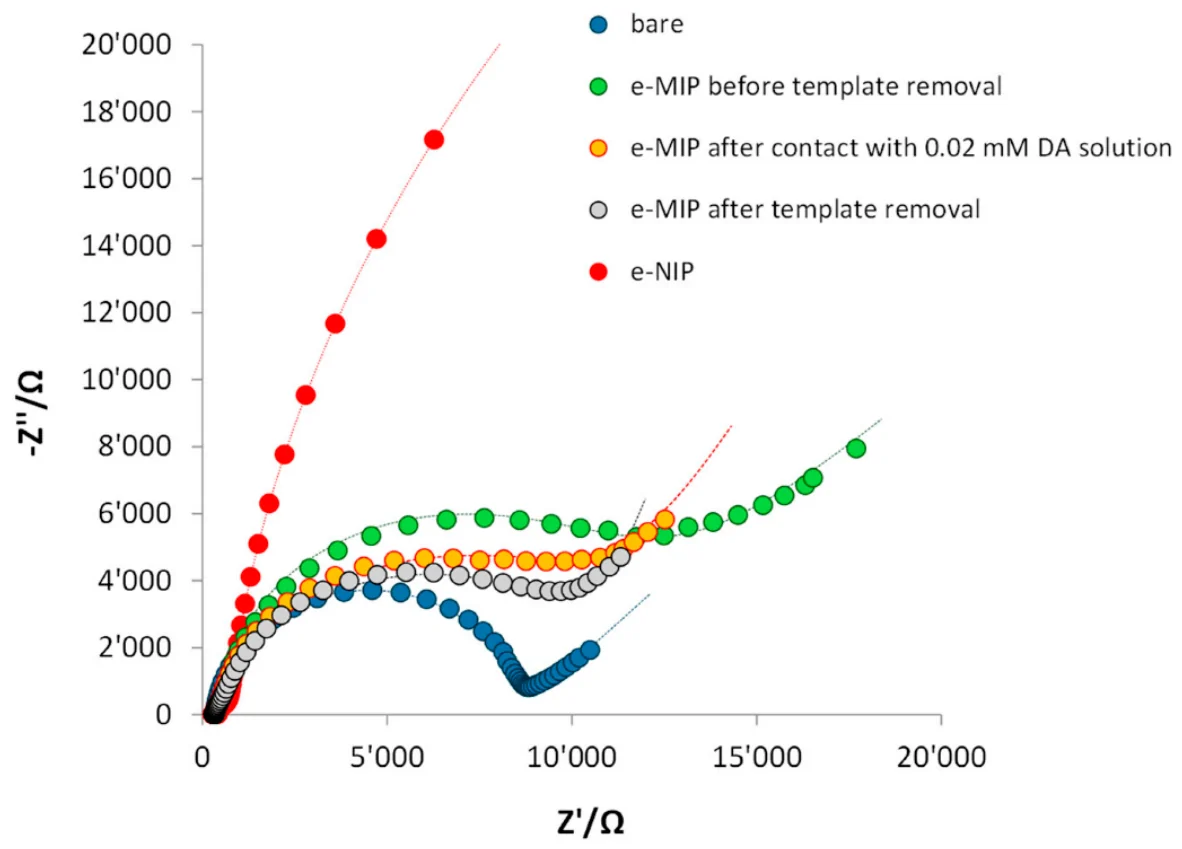
Nyquist plots (5 mM [Fe(CN)_6_]^3−^/^4−^, 0.1 M KCl) for the bare electrode, the e-MIP electrode before and after template removal, the e-MIP after rebinding with 0.02 mM dopamine, and the non-imprinted control (e-NIP). The charge-transfer resistance rises on MIP formation, drops after cavity opening, and increases again upon dopamine rebinding, while the e-NIP stays essentially unchanged. Reproduced from Merli et al. [103], Polymers 2024, under CC BY 4.0.

In EIS, a small alternating potential perturbation is applied over a range of frequencies to obtain the impedance spectrum. Two modes are distinguished: in faradaic EIS, the charge-transfer resistance (R_ct_) is detected via an external redox couple—most commonly [Fe(CN)_6_]^3−^/^4−^—whereas in non-faradaic (capacitive) EIS, no probe is added and the signal arises from binding-induced changes in the double-layer capacitance. [105]. Rebinding of the target within the imprinted cavities most frequently increases R_ct_ by hindering the redox probe, but the direction of the change is not universal: the interfacial factors indicated for voltammetry in Section 5.2.1 (film charge, swelling, permeability) can equally lower R_ct_ or modify C_dl_, so the response must be interpreted in the context of the specific film chemistry rather than assumed a priori. [105]. Spectra are represented as Nyquist or Bode plots (Figure 11), in which the R_ct_ of an electropolymerized MIP increases upon film formation, decreases after template removal, and increases again upon rebinding, whereas the non-imprinted control remains essentially unchanged [103].

In practice, the impedance change—usually expressed as ΔR_ct_ between the MIP and its NIP—correlates linearly with analyte concentration over wide dynamic ranges. Faradaic ΔR_ct_ readouts remain label-free with respect to the target, but not mediator-free, since they rely on the [Fe(CN)_6_]^3−^/^4−^ couple (Figure 11); fully reagent-free operation is achieved only in the non-faradaic mode [105].

#### 5.3.2. Advantages

MIP sensors based on EIS offer label-free operation, low detection limits frequently reaching the sub-nanomolar or picomolar regime, and compatibility with insulating MIP films on rigid or flexible substrates. These features make impedimetric transduction particularly well suited to proteins and pathogens, whose binding induces pronounced interfacial changes readily detectable as variations in R_ct_ [105,106]. Moreover, integration with microfabricated or screen-printed gold electrodes enables compact, low-power, multiplexed impedance platforms, favoring translation toward point-of-care and field diagnostic applications [106].

Impedance spectra are usually interpreted using Randles-type equivalent circuits, in which a constant-phase element replaces the ideal capacitor to account for film non-ideality and heterogeneity [106]. It should be emphasized that a good circuit fit reproduces the spectrum but does not by itself allow specific rebinding to be distinguished from non-specific adsorption or fouling, which increase R_ct_ to a comparable extent; discrimination relies on the NIP control, template removal/rebinding cycles, and selectivity tests, rather than on curve fitting alone [105,106].

### 5.4. Potentiometric and Field-Effect MIP Sensors

Beyond current and impedance, MIP recognition can also modulate the surface potential of transistors or ion-selective electrodes, enabling compact transduction schemes that directly convert binding-induced interfacial charge/dipole changes into electrical readouts.

#### 5.4.1. MIP-Gated Field-Effect Transistors (MIP-FETs)

In MIP-FETs, the imprinted layer acts as a molecularly selective gate dielectric (or extended-gate functional coating): analyte rebinding perturbs the local surface charge density and/or dipole distribution at the gate interface, producing a measurable shift in the effective threshold voltage (Vth) and corresponding transfer characteristics. A representative 2024 example couples extended-gate FET (EG-FET) signal transduction with epitope molecular imprinting to selectively detect pulmonary-fibrosis biomarkers, demonstrating the feasibility of translating epitope-level recognition into gate-potential modulation within a transistor architecture [107]. These devices are intrinsically compatible with miniaturisation and integration into lab-on-chip layouts, but their practical performance is constrained by several factors that must be addressed at the design stage. Debye screening confines the detectable charge to within approximately one Debye length of the gate, so that operation in high-ionic-strength media—such as undiluted physiological fluids—may require sample dilution, low-ionic-strength buffers, or confinement of the recognition sites close to the surface [107]. Threshold-voltage read-outs are further sensitive to the stability of the reference electrode and to baseline drift and hysteresis in the transfer characteristics, and the signal depends on ionic strength and pH; explicit reporting of these parameters, alongside matched NIP controls, is essential for reproducible and defensible MIP-FET data.

#### 5.4.2. Potentiometric and Ion-Selective Electrodes

In potentiometric formats, the MIP layer can replace (or complement) conventional ionophores within the sensing membrane, enabling selective detection of neutral or weakly ionic targets via binding-induced changes in membrane phase boundary potential. A 2024 study explicitly demonstrates that the choice of functional monomer in a MIP potentiometric membrane governs selectivity, showing improved discrimination when using a higher-affinity monomer (supported by DFT interaction analysis) and, consequently, superior potentiometric performance compared to a conventional MIP formulation [98]. These systems require simple instrumentation and are therefore well suited for portable and point-of-care implementations.

### 5.5. Signal Amplification and Hybrid Architectures

Given that molecular binding itself often produces small interfacial changes, signal amplification is crucial for high-performance MIP biosensors.

#### 5.5.1. Nanomaterial-Based Amplification

A foundational strategy leverages nanomaterial-based amplification: the filler phases listed in Section 4.5, when integrated within or beneath the MIP layer, significantly increase the effective surface area, enhance electron transfer pathways, and improve catalytic activity [108]. For example, graphene combined with flower-like Au nanostructures covalently assembled beneath an imprinted polymer film markedly increased sensitivity toward 2,4,6-trichlorophenol through enhanced interfacial conductivity and adsorption capacity [109]. Composites incorporating MXene and multi-walled carbon nanotubes (MWCNTs) have also been shown to facilitate charge mobility and amplify electrochemical responses in histamine detection, evidencing the broad utility of two-dimensional and fibrous nanomaterials for signal enhancement [110]. The same amplification logic now extends to atomically dispersed phases: it has been reported that single-atom catalysts and sub-nanometric metal nanoclusters integrated with printed layers enhance the electron transfer and electrocatalytic pathways for both small-molecule and protein targets (Section 4.5), with the caveat that the stability of the single sites under repeated potential cycling remains the limiting factor [89,90,91].

#### 5.5.2. Redox and Enzymatic Labels

Embedding redox-active mediators directly into the MIP matrix provides an alternative amplification mechanism. In a ratiometric imprinted electrochemical sensor for bisphenol A, an alkenyl ferrocene functional monomer served both as a recognition element and intrinsic redox label, enabling dual-signal readouts that improved reproducibility and reduced susceptibility to external interferences [111].

#### 5.5.3. Multiplexed and Ratiometric Detection

Hybrid nanocomposite amplification can further be extended to complex multicomponent sensing, such as MIP sensors incorporating MWCNTs and Au@Fe_3_O_4_ nanoparticles, where the synergistic effects of catalytic and conductive components yielded sensitive performance in the detection of anticancer drugs in biological matrices, illustrating the integration of amplification strategies with real sample analytics [112].

### 5.6. Regeneration, Reusability, and Operational Stability

Electrochemical MIP sensors typically exhibit excellent reusability, which is a practical advantage over antibody-based recognition layers in repeated-measurement workflows. Regeneration is commonly achieved by disrupting template–cavity interactions through solvent washing, potential cycling/electrochemical desorption, or controlled pH modulation, thereby enabling multiple sensing–desorption cycles with limited loss of performance. In representative electropolymerized platforms, signal retention can remain above ~90% even after ~30 consecutive regeneration/rebinding cycles, highlighting the mechanical robustness of highly crosslinked or surface-anchored imprinted films and the stability of the resulting binding-site architecture [113]. The constraints that skin-contacting and wearable formats impose on these protocols are considered separately in Section 8.6, and the reporting requirements for regeneration data in Section 9.5.

Operational stability in complex matrices further benefits from anti-fouling and protective-yet-permeable interfacial designs, which mitigate non-specific adsorption and mechanical wear while preserving analyte access to the imprinted layer. Recent “ultra-low fouling” MIP electrochemical designs demonstrate that engineered interfacial architectures can effectively resist foulants in real samples without compromising selective recognition, supporting the general strategy of adding protective transport-permissive barriers or functional interlayers to maintain baseline stability over time [114].

### 5.7. Analytical Performance Benchmarks

Modern electrochemical MIP-based biosensors have reached analytical performances that rival, and in selected cases surpass, those of conventional immunosensors, while retaining superior operational robustness and cost efficiency. As best-case reported values, LOD for small-molecule targets have been placed in the low femtomolar to sub-picomolar range (≈10^−14^ M) in optimized electrochemical platforms, accompanied by high selectivity against structurally related interferents present at orders-of-magnitude higher concentrations [100]. For proteins and cytokines, reported detection limits span the femtomolar to low picomolar range (≈10^−11^–10^−14^ M), with individual point-of-care platforms reaching the femtomolar or even attomolar level for biomarkers such as GFAP [115,116]. These figures are best-case values obtained under favourable conditions rather than typical or guaranteed performance, and their origin should be made explicit: such limits are system-level properties governed by electrocatalytic or nanomaterial-based signal amplification and enlarged electroactive area and, as established in Section 2.4, do not imply a correspondingly low affinity (K_d_) of the imprinted cavities themselves (Table 1; Section 9.3 and Section 9.4). Whether a value was obtained in buffer, in diluted matrix or in an undiluted real sample is decisive for its interpretation and should always be stated.

Dynamic linear ranges spanning up to five–six orders of magnitude are commonly achieved, particularly in nanostructured or transistor-amplified architectures, enabling reliable quantification across physiologically relevant concentration windows [116,117]. Owing to thin-film configurations and interfacial amplification strategies, response times are typically on the order of seconds and often below 10 s, supporting real-time and near-real-time monitoring [117].

Selectivity factors exceeding one order of magnitude over closely related analogues are frequently observed, reflecting the effectiveness of molecular imprinting in discriminating target-specific interactions even under competitive binding conditions [100,115]. Finally, the widespread adoption of screen-printed and flexible electrode platforms facilitates scalable manufacturing and integration into portable and point-of-care devices—the manufacturing maturity of these routes is assessed in Section 8.4—underscoring the translational potential of electrochemical MIP biosensors beyond laboratory demonstrations, including sustainable and bioderived point-of-care formats [11,116,118].

The comparability of these figures across studies, and the reporting practices required to secure it, are addressed in Section 9; the same caveats carry over to the optical and mass-sensitive platforms considered next, to which the discussion now turns.

## 6. Transduction Strategies II: Optical, Mass-Sensitive and Other MIP-Based Biosensors

While electrochemical methods dominate molecularly imprinted polymer (MIP) biosensing, optical and mass-sensitive transduction platforms offer complementary advantages, including label-free operation, high sensitivity, multiplexing capability, and straightforward miniaturization for point-of-care diagnostics. In these systems, analyte binding at the imprinted surface either alters optical properties—such as refractive index, fluorescence, or scattering—or modifies the mass and viscoelastic behaviour of an oscillating substrate. Representative platforms include plasmonic MIP sensors based on surface plasmon resonance (SPR/LSPR), fluorescent MIP platforms employing luminescent nanomaterials, and acoustic devices exploiting frequency shifts induced by analyte adsorption, reflecting the growing convergence between molecular imprinting, nanophotonics, and advanced sensing technologies [119,120].

Optical transduction relies on monitoring changes in light–matter interactions upon analyte recognition. These interactions can be intrinsic, when the analyte itself is optically active, or extrinsic, when binding modifies the optical properties of the MIP or its surrounding environment. Accordingly, the optical modalities are examined first—plasmonic (Section 6.1), fluorescent/luminescent (Section 6.2), and colorimetric (Section 6.3)—followed by mass-sensitive (Section 6.4) and hybrid optical–electrochemical platforms (Section 6.5) [119].

### 6.1. SPR and Localized SPR (LSPR) MIP Sensors

In SPR sensing, the MIP layer is deposited on a gold or silver film supporting surface plasmons—collective electron oscillations highly sensitive to refractive-index changes near the metal–dielectric interface. Upon analyte binding, the refractive index of the MIP layer changes, producing a measurable resonance-angle or wavelength shift; SPR-based MIPs therefore combine the chemical selectivity of imprinting with real-time, label-free optical quantification [120].

**Figure 12:**
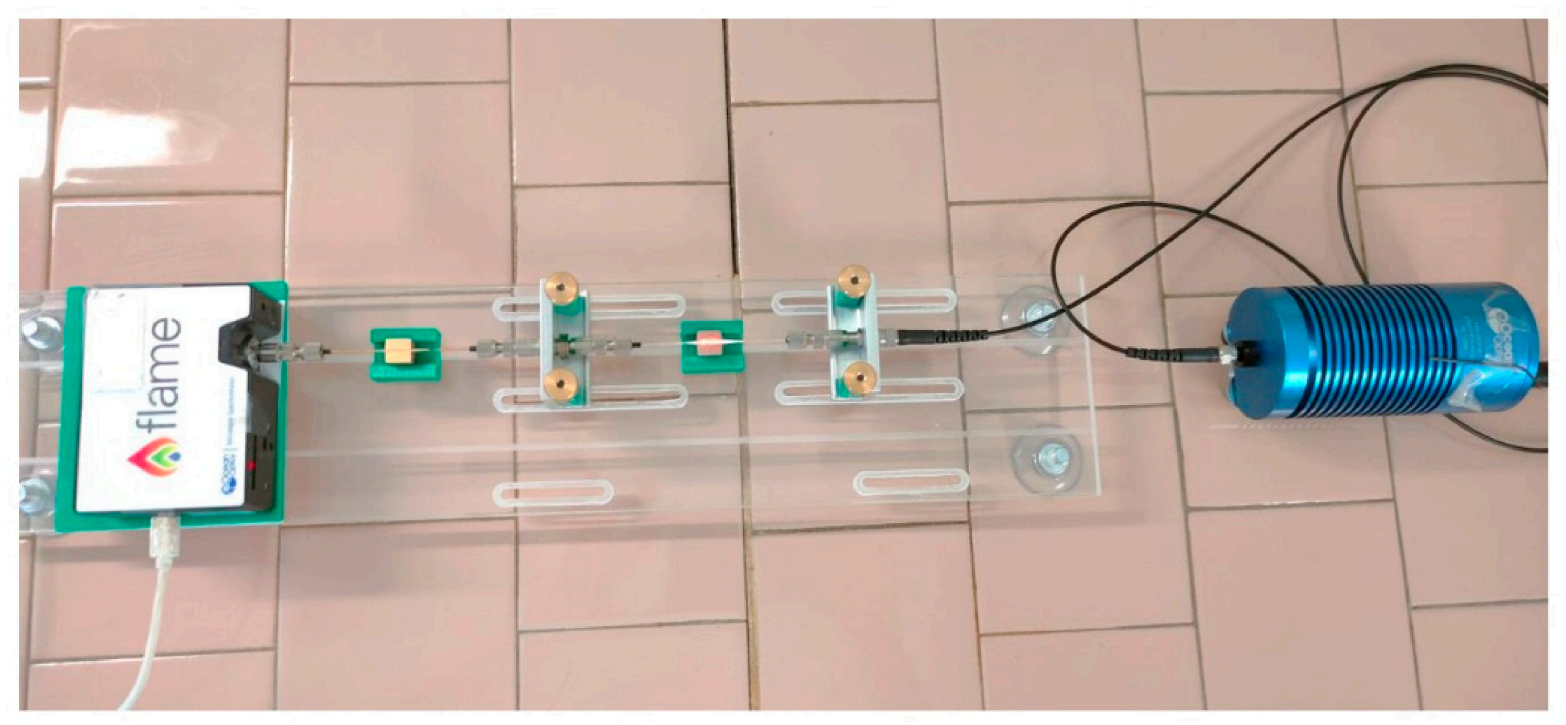
Experimental setup for glyphosate sensing based on a molecularly imprinted three-hole plastic optical-fibre (POF) chip coupled in series to a gold-coated SPR-POF platform: a halogen source illuminates the MIP-functionalized chip, whose analyte-induced refractive-index change modulates the SPR read out by the spectrometer. Reproduced from Alberti et al. [121], Chemosensors 2023, 11, 414, under CC BY 4.0.

Typical platforms include planar SPR chips, SPR imaging arrays, nanoplasmonic configurations, and fibre-optic SPR sensors. For example, a plastic optical-fibre SPR platform coated with a thin MIP layer enabled glyphosate detection from nanomolar to micromolar concentrations with a limit of detection of about 1 nM, supporting miniaturized, portable MIP–SPR configurations for on-site analysis [121]. The corresponding experimental configuration—a MIP-filled three-hole POF chip optically coupled in series to a gold-coated SPR-POF platform—is shown in Figure 12.

At the nanoscale, localized SPR (LSPR) exploits metallic nanoparticles whose resonance depends on local dielectric changes; chip-LSPR platforms integrating magnetic molecularly imprinted nanoparticles have been reported for the sub-ng mL^−1^ detection of sulfonamide antibiotics such as sulfadiazine, in which the imprinted nanoparticles act simultaneously as selective adsorbents and as LSPR signal-amplifying elements [120].

### 6.2. Fluorescent and Luminescent MIP Sensors

**Figure 13:**
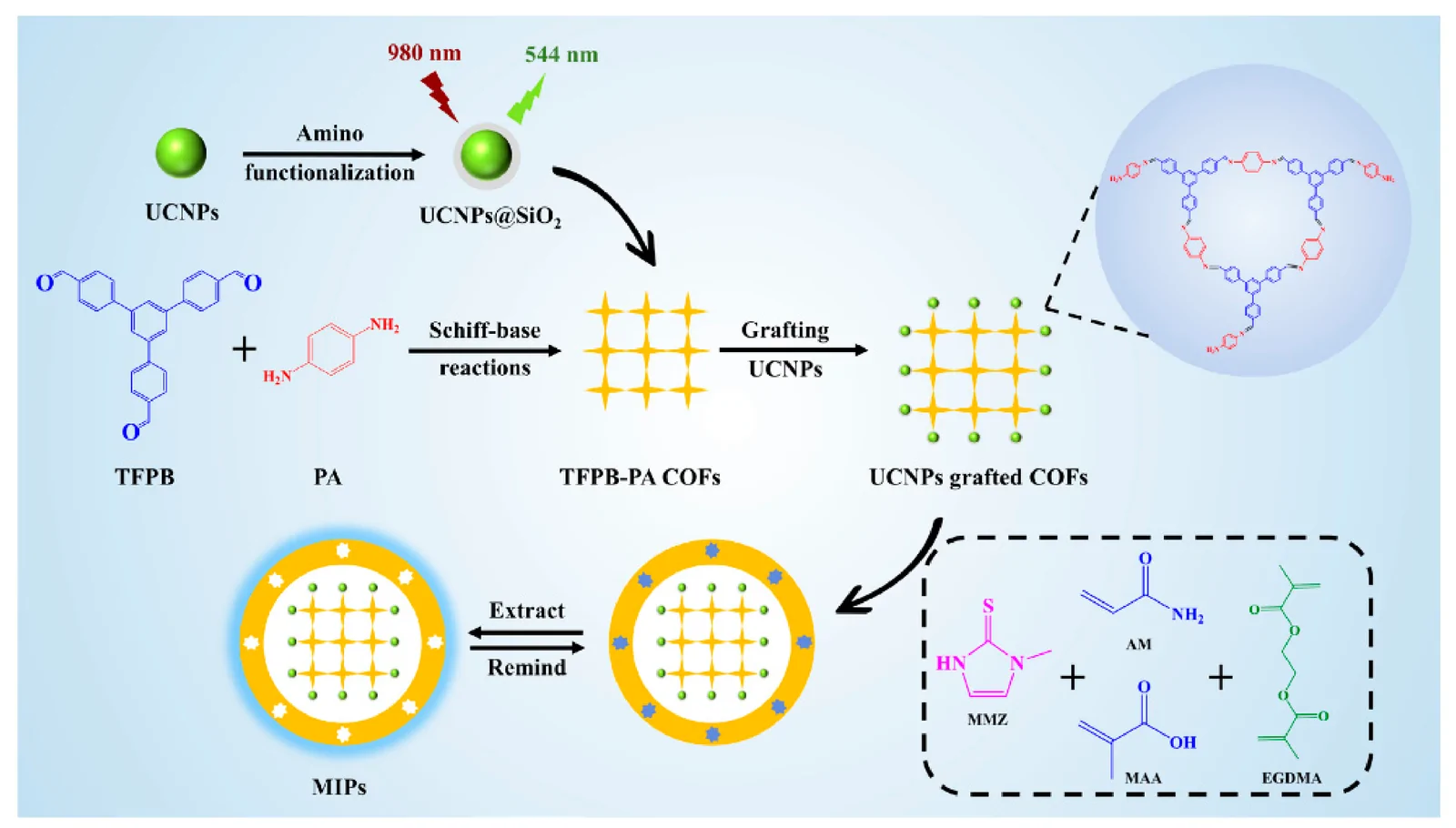
Preparation route of the upconversion-nanoparticle-grafted covalent-organic-framework molecularly imprinted sensor (UCNP-grafted COFs@MIP): UCNPs are anchored onto the COF support and coated with a molecularly imprinted layer whose cavities selectively rebind methimazole, quenching the upconversion fluorescence. Reproduced from Liu et al. [122], Processes 2024, 12, 626, under CC BY 4.0.

Fluorescence-based detection remains a highly sensitive strategy for real-time, ratiometric, and multiplexed biosensing. MIPs can act as fluorescence modulators when analyte binding affects energy-transfer pathways, local polarity, or the microenvironment around the emissive component; in luminescent MIPs, lanthanide-doped or upconversion nanoparticles extend sensing into low-background optical regimes. For example, a fluorescent MIP based on upconversion nanoparticles grafted onto a covalent organic framework was reported for methimazole detection: the UCNP–COF@MIP system showed optical stability, selective recognition, anti-interference capability, and a linear response from 0.05 to 3 mg L^−1^ with a detection limit of 3 µg L^−1^ in real samples [122]. The preparation route and sensing principle of this UCNP-grafted COFs@MIP probe are summarized in Figure 13.

### 6.3. Colorimetric and Visual MIP Sensors

Colorimetric MIP sensors offer simple, naked-eye or smartphone-assisted detection without sophisticated instrumentation. Binding-induced optical changes can arise from chromogenic reactions modulated by selective rebinding, nanozyme activity, or swelling-induced changes in photonic structures embedded within the imprinted network [123].

#### 6.3.1. Paper-Based and Hydrogel MIPs

MIPs integrated into cellulose paper, hydrogels, or portable analytical devices enable low-cost, disposable assays suitable for on-site analysis. For instance, paper-based devices incorporating MIP-modified g-C_3_N_4_ nanozymes have been used for the colorimetric detection of amoxicillin, where analyte rebinding inhibits the peroxidase-like oxidation of TMB and produces a concentration-dependent decrease in the blue signal, allowing both visual and smartphone-assisted readout in milk and tap-water samples [123]. Hydrogel-based MIPs can likewise act as visual sensors when analyte rebinding induces swelling or deswelling of the polymer matrix, a mechanism exploited in the photonic-crystal formats discussed in Section 6.3.2.

#### 6.3.2. Photonic Crystal and Structurally Coloured MIPs

Two- or three-dimensional colloidal photonic crystals embedded within MIP networks act as optical transducers because analyte rebinding changes particle spacing or lattice periodicity, thereby shifting the reflected or diffracted wavelength. For example, a molecularly imprinted 2D photonic-crystal hydrogel was reported for sodium dichloroisocyanurate: increasing analyte concentration produced measurable changes in the Debye diffraction-ring diameter and in the particle spacing, enabling label-free visual and spectral detection in a real disinfectant-powder sample [124]. Such structurally coloured MIPs provide reusable, label-free, and visually interpretable platforms for portable sensing.

### 6.4. Mass-Sensitive MIP Biosensors: QCM and Acoustic-Wave Platforms

Mass-sensitive transduction provides a complementary route for monitoring molecular recognition events at imprinted interfaces. In these devices, analyte rebinding to a MIP layer produces a change in interfacial mass loading and, in some cases, in the viscoelastic properties of the sensing film. These changes are converted into measurable variations in the resonance behaviour of an oscillating piezoelectric substrate, enabling label-free, real-time monitoring of binding processes.

#### 6.4.1. Quartz Crystal Microbalance (QCM)

QCM sensors are among the most widely used mass-sensitive platforms for studying adsorption and recognition at functionalized surfaces. In a typical configuration, a thin MIP film is deposited onto a gold-coated quartz resonator; rebinding of the target within the imprinted cavities increases the effective mass coupled to the oscillating surface, producing a decrease in resonance frequency. Under ideal rigid-film, low-dissipation conditions, this frequency change is related to the adsorbed mass through the Sauerbrey equation:

(2) $$\Delta f=-\frac{2f_{0}^{2}\Delta m}{A\sqrt{\rho_{q}\mu_{q}}},$$

where Δf is the measured frequency shift, Δm is the mass adsorbed on the resonator surface, f_0_ is the fundamental resonance frequency, A is the piezoelectrically active electrode area, and ρq and μq are the density and shear modulus of quartz, respectively. This principle has been applied, for example, to sol–gel MIP films coated on QCM crystals for the selective, real-time detection of melamine in milk [125].

The main advantage of QCM-MIP sensing is that it allows direct, label-free assessment of analyte rebinding at the polymer interface, making it particularly useful for investigating MIP–template interactions, comparing imprinted and non-imprinted films, and characterizing the kinetics and reversibility of recognition. In aqueous media, however, the interpretation of QCM signals requires caution, because frequency shifts may reflect not only dry-mass uptake but also coupled solvent, film swelling, and viscoelastic dissipation; for this reason, QCM-D or complementary optical/electrochemical measurements are often advantageous when soft, hydrated, or hydrogel-like MIP layers are used.

Compared with electrochemical and optical MIP biosensors, recent QCM-MIP demonstrations are less numerous. Nevertheless, QCM remains valuable for real-time monitoring of selective adsorption, especially when MIP films are engineered to be thin, hydrophilic, and mechanically stable under flow.

#### 6.4.2. Surface Acoustic Wave (SAW) and Related Acoustic-Wave Devices

SAW sensors and related acoustic-wave devices extend mass-sensitive transduction to wave propagation along piezoelectric substrates. When a MIP coating captures the target analyte, the added mass and possible viscoelastic damping modify the acoustic-wave velocity, phase, amplitude, or resonance frequency. In principle, these features make SAW-MIP devices attractive for rapid, miniaturized sensing, particularly in gas-phase or environmental monitoring, where low sample volumes and fast interfacial equilibration are advantageous.

However, the recent literature on SAW-MIP biosensors remains comparatively limited relative to electrochemical, SPR, fluorescence, and QCM-based formats; SAW-MIP systems remain, for now, a promising but comparatively immature transduction route. Their future development will depend on improved control of MIP film thickness, acoustic compatibility of the polymer layer, suppression of non-specific adsorption, and validation in complex aqueous or biological matrices.

Overall, mass-sensitive MIP sensors provide a useful bridge between analytical sensing and interfacial materials characterization. QCM and SAW platforms offer label-free, real-time readout of binding-induced mass and mechanical changes; their role in MIP biosensing today is thus complementary rather than dominant.

### 6.5. Emerging Optical–Electrochemical Hybrid Systems

The convergence of optical and electrochemical transduction represents an emerging direction in advanced MIP-based sensing. Rather than relying on a single readout mechanism, hybrid platforms combine the molecular selectivity of imprinted polymers with complementary signal-generation pathways—such as light-induced charge separation, optical modulation, and interfacial electron-transfer processes—improving analytical reliability by providing orthogonal or semi-orthogonal information on the same binding event, and thereby helping to distinguish specific recognition from non-specific adsorption.

Among these strategies, photoelectrochemical (PEC) MIP sensors are currently the most clearly established. In PEC-MIP devices, a semiconductor photoactive layer—such as TiO_2_, ZnO, BiVO_4_, CdS, or related heterostructures—generates photocurrent under illumination, while the MIP layer introduces molecular selectivity by controlling access of the target to the photoactive interface or by modulating interfacial charge transfer after rebinding. Target recognition thus produces a measurable change in photocurrent, enabling light-driven detection with low background signals and good sensitivity, as demonstrated for antibiotics such as chlortetracycline using rGO/semiconductor heterojunctions [126].

Other hybrid concepts remain at an earlier stage. Electrochromic or optically readable redox-active MIP films could, in principle, convert analyte binding into absorbance or colour changes through redox-induced modulation of the polymer or an embedded chromophore, while dual-readout architectures combining optical monitoring with electrochemical impedance or voltammetry may provide additional discrimination between target-specific binding and non-specific fouling. These configurations are, however, less mature than purely electrochemical, plasmonic, fluorescence, or PEC-MIP sensors and should currently be regarded as emerging rather than established.

Overall, optical–electrochemical hybrid MIP systems align with the broader trend toward smart, self-calibrating, and multiplexed biosensors. Their development will likely depend on the rational integration of imprinted recognition layers with stable photoactive materials, redox-responsive polymers, antifouling interfaces, and miniaturized readout electronics; if successfully implemented, such multimodal designs could improve selectivity, reduce false-positive responses, and support more robust point-of-care and environmental monitoring devices.

### 6.6. Performance Overview and Applications

Optical and acoustic MIP sensors thus complement their electrochemical counterparts by offering visual, label-free, and multi-parameter outputs essential for portable and field-deployable biosensing. Plasmonic and photonic-crystal formats provide rapid, label-free optical readout; fluorescent and upconversion systems deliver high sensitivity and low optical background; colorimetric and paper-based devices enable naked-eye, on-site screening; and mass-sensitive and photoelectrochemical platforms add complementary, orthogonal information for improved reliability. Taken together with the electrochemical methods of Section 5, these strategies define the readout options available to the designer; which of them is appropriate, however, depends primarily on the nature of the target, and it is by target class that the following section is organised.

## 7. MIP for Different Bio-Targets: From Small Molecules to Cells

The versatility of MIPs lies in their ability to selectively recognize a wide spectrum of analytes, from ions and metabolites to proteins, viruses, and whole cells. Unlike biological receptors, whose specificity is evolutionarily constrained to particular molecular families, MIPs can in principle be templated on any target able to engage the reversible interactions catalogued in Section 2.2. The choice of template type and imprinting strategy determines the structural and physicochemical features of the resulting polymer, dictating binding efficiency, selectivity, and kinetic response. This section provides an overview of MIP design across molecular scales, highlighting key successes and persistent challenges.

### 7.1. Small Molecules and Metabolites

Small molecules—such as drugs, hormones, pesticides, endocrine disruptors, and metabolites—represent the most mature application domain of MIP-based sensors. Their low molecular weight, defined geometry, and relatively stable conformations make them suitable templates for imprinting, allowing the formation of binding cavities with shape and functional complementarity. Representative recent examples include MIP sensors for pharmaceuticals, dopamine, tryptophan, hydrocortisone, and other low-molecular-weight bioactive compounds [82,127].

#### 7.1.1. Typical Targets

Within this domain, reported systems span antiviral drugs and antibiotics, catecholamine and amino-acid metabolites, and steroids; the last of these illustrate the suitability of molecular imprinting for structurally related hydrophobic targets in complex matrices [82,97,128,129,130,131,132]. Where these same metabolites (e.g., cortisol, tryptophan, lactate) are pursued in on-body formats, their device-level implementation is treated in Section 8.3 and is not reproduced here; this section is confined to the target-driven design rationale.

#### 7.1.2. Design Strategies

**Figure 14:**
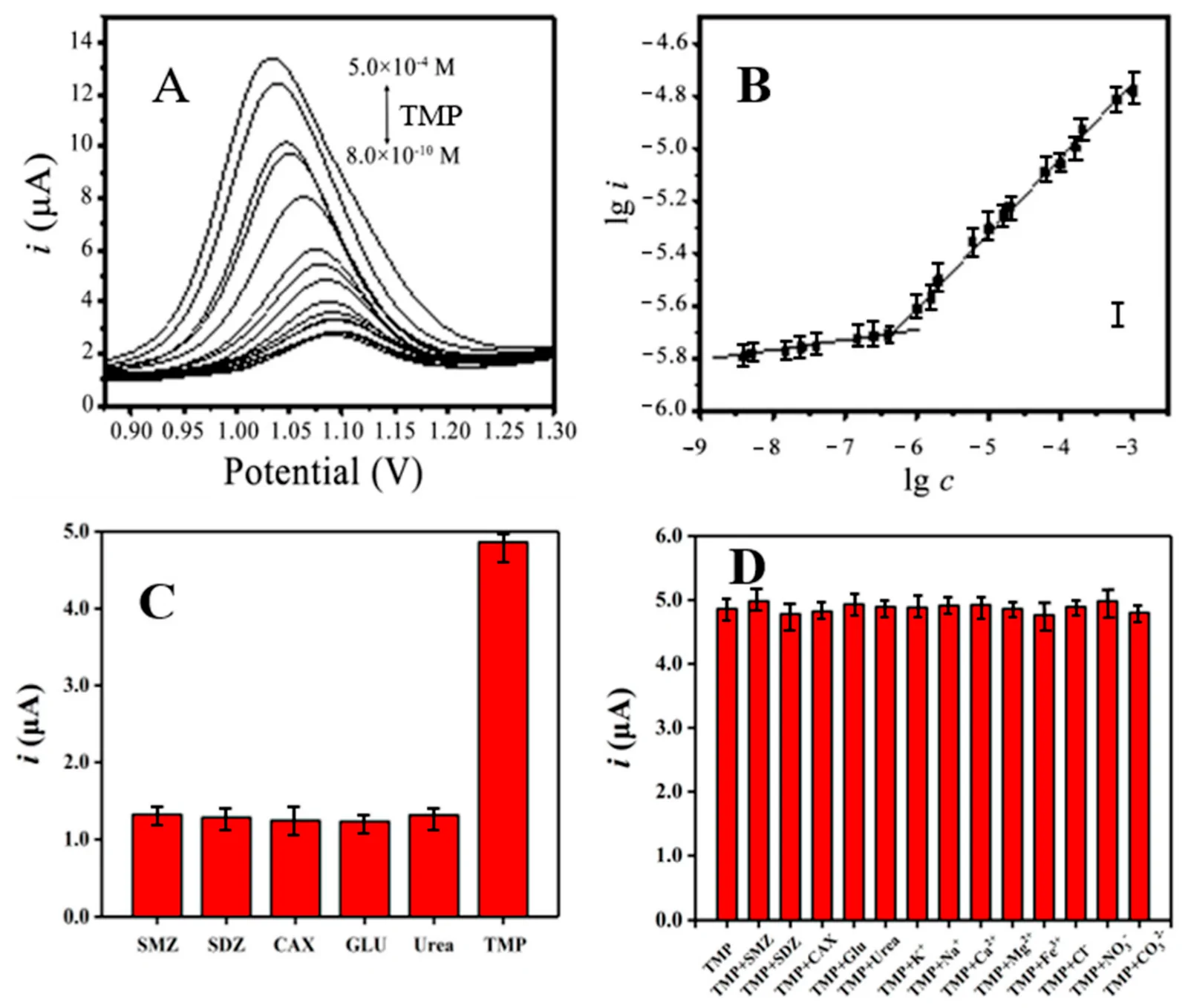
Electrochemical response of a small-molecule molecularly imprinted sensor based on a magnetic MIP combined with multi-walled carbon nanotubes and reduced graphene oxide as signal-amplifying supports: (**A**) differential-pulse voltammograms at increasing analyte concentrations, (**B**) the corresponding calibration relationship, and (**C**,**D**) selectivity and interference tests against structurally related species. Reproduced from Liu et al., Chemosensors 2023, 11(6):339 [128], under the terms of the Creative Commons CC BY 4.0 license.

For small molecules, non-covalent imprinting remains the dominant approach, exploiting the weak, reversible monomer–template interactions described in Section 2.2.1. MAA, 4-VP, AAm derivatives, and electropolymerizable monomers are frequently selected depending on the target structure and transducer. For aqueous biosensing, the hydrophilic layers, antifouling interfaces, and conductive nanocomposite supports catalogued in Section 3.6 and Section 4.5 are combined to improve mass transport, suppress non-specific adsorption, and amplify the signal [128,129,131,132]. In optimized devices, reported detection limits can reach the nanomolar–femtomolar range; consistent with the distinction drawn in Section 5.7 and Section 9.4, these are best-case, system-level values—analyte-, matrix- and transducer-dependent—rather than a universal feature of MIP sensors or a measure of cavity affinity [127,129]. A representative small-molecule example is shown in Figure 14, where a molecularly imprinted layer combined with carbon-nanomaterial supports yields a concentration-dependent voltammetric response with good selectivity against structurally related interferents.

### 7.2. Peptides and Proteins

The constraints that macromolecular templates impose—size, conformational lability, hydration, denaturation risk, and slow diffusion in crosslinked networks—were introduced in Section 3.4 and Section 3.5. This subsection considers how they have been addressed in practice for peptide and protein targets: through surface imprinting, epitope imprinting, and soft hydrated interfaces rather than conventional bulk imprinting.

#### 7.2.1. Surface Imprinting of Proteins

In surface imprinting, the protein template is immobilized or confined close to the sensor interface before polymerization, producing recognition sites that are exposed at, or near, the MIP surface. Recent examples include MIP films for protein recognition using optical or electrochemical transduction, where the signal arises from analyte rebinding at surface-accessible imprinted sites rather than diffusion into a bulk polymer phase [133,134].

#### 7.2.2. Epitope Imprinting

Epitope-imprinted MIPs have been applied to clinically relevant proteins and viral targets, including cancer biomarkers and viral capsid/protein fragments, with successful operation in serum, saliva, or aqueous biological media [135,136].

#### 7.2.3. Protein–Polymer Interface Considerations

Protein imprinting requires polymer matrices that balance shape retention with hydration and conformational compatibility. The soft hydrogel, zwitterionic, and water-compatible nanogel chemistries of Section 3.7 and Section 4.1.3 are therefore particularly well matched to this target class, since they reduce non-specific adsorption while preserving site accessibility. Because protein-MIP performance depends strongly on the imprinting format, template immobilization strategy, film thickness, and matrix composition, reported figures of merit are case-specific: antibody-like sensitivity demonstrated for one construct cannot be assumed to transfer to protein imprinting as a whole.

### 7.3. Nucleic Acids and Aptamer–MIP Hybrids

Nucleic acids represent challenging targets for molecular imprinting because DNA and RNA strands combine high charge density, sequence-dependent conformational flexibility, hydration effects, and strong non-specific electrostatic interactions. Compared with small molecules and proteins, direct imprinting of oligonucleotides is therefore less mature and requires careful control of ionic interactions, template orientation, and polymer hydration.

#### 7.3.1. Direct Imprinting of Oligonucleotides

Direct imprinting of short oligonucleotides or nucleic-acid fragments can, in principle, be promoted by cationic or hydrogen-bonding monomers able to interact with the phosphate backbone and nucleobases. In such systems, recognition may arise from a combination of electrostatic complementarity, base-specific hydrogen bonding, and steric matching. However, because non-specific binding of polyanionic templates can be substantial, direct oligonucleotide-imprinted MIPs should still be regarded as less established than small-molecule, peptide, or protein-imprinted systems. Claims of single-nucleotide discrimination or sub-nanomolar detection should therefore be restricted to specific experimentally validated examples. Indeed, the great majority of MIP-based nucleic-acid sensors reported to date do not rely on direct imprinting of the free oligonucleotide backbone; instead, they exploit alternative recognition routes—such as imprinting of nucleobases, nucleoside derivatives, or hybridization-mediated readout—so that true direct oligonucleotide imprinting remains a niche and still largely proof-of-concept approach.

#### 7.3.2. Aptamer–MIP Hybrid Systems

Aptamer–MIP hybrids offer an alternative strategy by combining the sequence-defined recognition of aptamers with the mechanical and chemical robustness of an imprinted polymer scaffold. In these systems, polymerizable aptamers or aptamer-functionalized monomers can participate in the formation of the imprinted network, while the polymer matrix stabilizes the recognition environment and can improve resistance to degradation or environmental variation. This dual-recognition concept is attractive because the aptamer provides target-specific molecular recognition, whereas the MIP contributes structural memory and material stability. Nevertheless, recent aptamer–MIP systems remain relatively specialized, and their analytical performance depends strongly on aptamer design, immobilization chemistry, polymer composition, and target accessibility [137].

A clinically relevant special case is the detection of short, low-abundance nucleic acids such as microRNAs miR-21 and miR-141, whose dysregulation is diagnostic and informative in several types of cancer. Direct imprinting has been used to generate selective sites for miR-21 on silica-supported polymers for enrichment [138] and, more recently, imprinted nanoparticles have been coupled to surface plasmon resonance readout for one-step detection of miR-21 directly in serum [139], indicating that the hurdle is not so much the imprinting step itself, but rather reaching the ultratrace and high-background regime in which these targets are found. Two directions are being explored to bridge this gap. First, constraining and pre-organizing the imprinted recognition motif, for example by orienting an oligonucleotide- or aptamer-derived probe within the network (Section 7.3.2), can improve discrimination of single-base differences among members of closely related miRNA families. Second, transduction gain can be added downstream of recognition, either through aggregation-induced emission (AIE) fluorogens, which have already been incorporated into imprinted hydrogels to convert selective binding into an activatable optical signal [140,141], or through enzyme-free nucleic acid circuits (e.g., hybridization chain reaction or catalytic hairpin assembly) that amplify a single binding event. It should be emphasized that MIP systems for miRNA based on AIE and coupled circuits remain largely still in the development stage and that, in line with the distinction between affinity and LOD developed in Section 2.4, any resulting sensitivity enhancement originates in transduction and amplification rather than in the affinity of the imprinted cavity itself.

### 7.4. Viruses, Bacteria, and Whole-Cell Imprinting

Extending molecular imprinting to viruses, bacteria, and eukaryotic cells shifts the design problem from molecular recognition to interfacial and topographical complementarity. In these cases, the imprinted polymer does not reproduce an entire molecular structure at atomic resolution; rather, it forms a complementary surface pattern that reflects the size, shape, charge distribution, and biochemical features of the biological template. This approach is promising for selective capture and label-free detection, but it also introduces major challenges related to template removal, deformation of soft biological objects, non-specific adhesion, and variability among strains or cell phenotypes.

#### 7.4.1. Virus Imprinting

Virus imprinting generally relies on surface imprinting, epitope imprinting, or imprinting against viral proteins rather than true three-dimensional replication of whole virions. For virions, this surface pattern is dominated by accessible protein and glycoprotein motifs alongside overall particle morphology. Relative to small-molecule and protein imprinting, virus imprinting is at an earlier stage of technological development, with fewer independently reproduced device demonstrations. They are therefore best positioned as low-cost, robust screening interfaces that complement, rather than replace, nucleic-acid amplification or immunodiagnostic methods [142].

#### 7.4.2. Bacterial Imprinting

**Figure 15:**
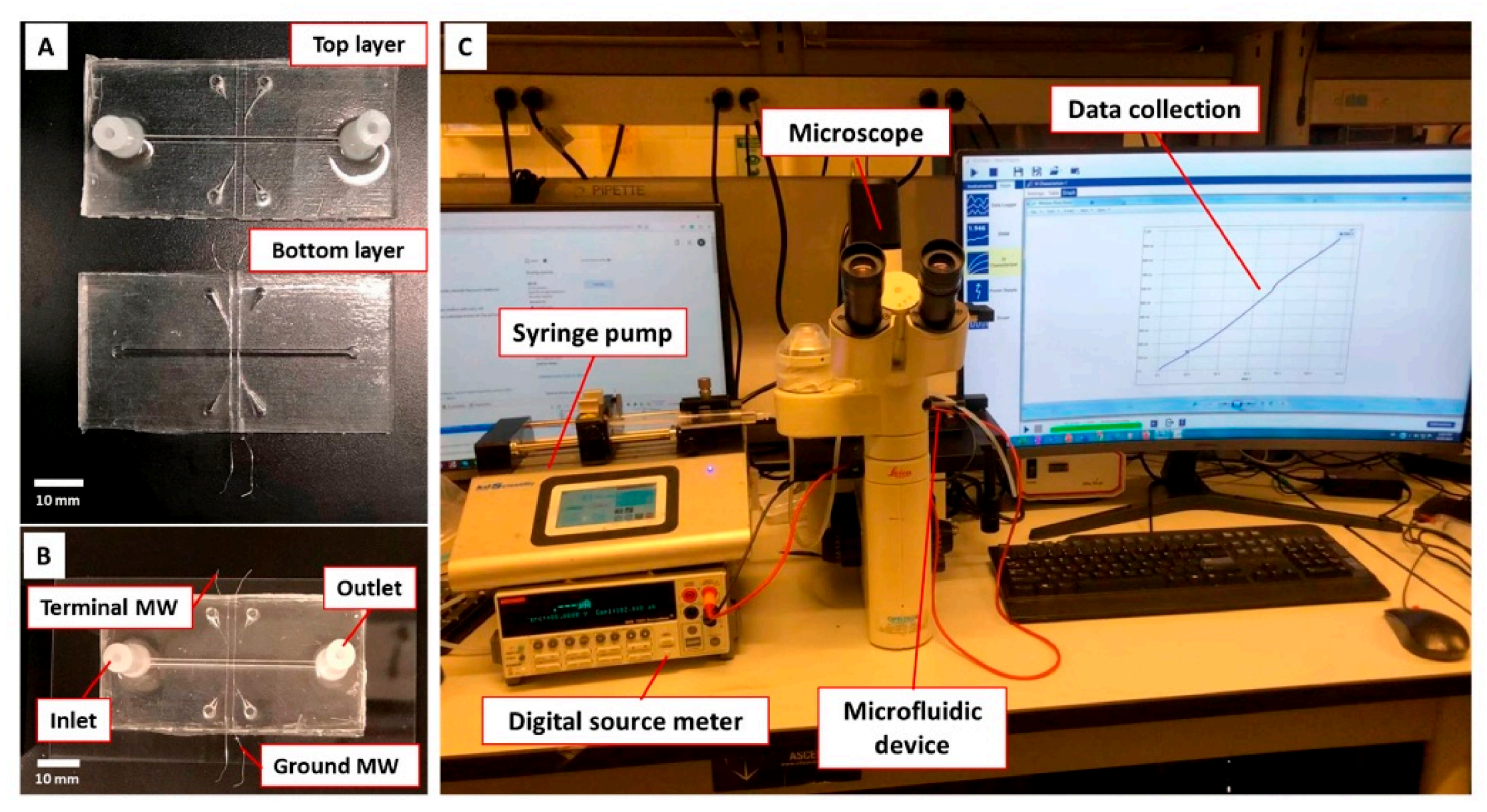
Cell-imprinted polymer sensor for whole-bacteria recognition: (**A**) the PDMS layers with the installed cell-imprinted microwires, (**B**) the assembled microfluidic device after plasma bonding, and (**C**) the experimental setup for conductometric detection. Recognition arises from complementarity of cell size, shape, and surface features rather than a single molecular epitope. Reproduced from Akhtarian et al., Biosensors 2023, 13(10):943 [144], under the terms of the Creative Commons CC BY 4.0 license.

Bacterial imprinting typically uses microcontact imprinting, soft lithography, or surface-confined polymerization to capture the morphology and surface chemistry of intact cells. After removal of the template bacteria, the polymer surface contains recognition sites that can selectively rebind cells with matching size, shape, and envelope characteristics [143,144]. This strategy is particularly relevant for food safety, water monitoring, and environmental microbiology, where rapid capture and enrichment may be as important as analytical quantification. However, selectivity is influenced by bacterial growth phase, strain variability, surface appendages, and biofilm formation; therefore, numerical claims such as fixed cross-reactivity thresholds should only be reported when supported by a specific experimental study. An example of this whole-cell recognition implemented in a device is shown in Figure 15, where cell-imprinted microwires are integrated into a microfluidic channel to capture bacteria by size and surface complementarity.

#### 7.4.3. Eukaryotic Cell and Tissue Imprinting

At larger length scales, cell-imprinted polymers have been explored for recognition and capture of mammalian cells, including pathological or cancer-related phenotypes [145,146]. In these systems, recognition relies primarily on membrane topography, surface glycans, protein patterns, and mechanical matching rather than on a single molecular epitope. Cell imprinting may therefore provide useful interfaces for selective cell capture, enrichment, and downstream analysis. The main open challenge here is biological rather than technical: because eukaryotic cells are deformable, heterogeneous, and phenotype-dependent, recognition patterns vary across cell populations, so these platforms are best suited to selective capture and enrichment rather than to quantitative diagnostic readout.

### 7.5. Hierarchical and Multi-Analyte Imprinting

Because real biological samples rarely reflect a single diagnostically relevant molecule, physiological and pathological states usually manifest as correlated changes across panels of metabolites, hormones, proteins, and inflammatory mediators. This has stimulated growing interest in hierarchical and multi-analyte molecular imprinting strategies, where recognition sites with different sizes, chemical functionalities, or spatial locations are integrated within the same material or device.

In hierarchical imprinting, the polymer architecture is designed to contain binding domains operating at different length scales. Smaller cavities can recognize metabolites or drug molecules, whereas larger surface-accessible regions may interact with peptides, proteins, or cell-surface motifs. Such architectures are particularly attractive for complex matrices, because they can combine molecular selectivity with improved mass transport and reduced steric hindrance. However, the simultaneous imprinting of structurally and chemically different templates remains challenging, since each template may require different monomer–template interactions, crosslinking density, solvent polarity, and extraction conditions.

Multi-template MIPs provide a more direct route toward simultaneous recognition of related analytes. In these systems, two or more templates are introduced during polymerization to generate distinct populations of binding sites within a single polymer matrix. This approach is most effective when the target analytes share compatible imprinting conditions but retain sufficiently different structural or electrochemical signatures to be distinguished during readout. For this reason, multi-template MIPs are especially promising for families of structurally related small molecules, such as drugs, metabolites, pesticides, or endocrine disruptors [147,148].

An alternative strategy is spatially resolved imprinting, in which different MIP layers or microdomains are patterned on separate regions of the same transducer. This design avoids competition between templates during polymerization and reduces cross-talk between recognition sites. When coupled with electrode arrays, optical microarrays, or microfluidic sampling, spatially resolved MIP platforms may enable multiplexed detection while preserving the advantages of single-template optimization [149].

The decisive open question for hierarchical and multi-analyte imprinting is validation of simultaneous selectivity: reliable deconvolution of overlapping responses and control of cross-talk between binding-site populations still need to be demonstrated across realistic analyte panels. These systems anticipate future integrated biosensors capable of correlating multiple biochemical signals in real time, but further work is needed to validate their selectivity, calibration stability, and reproducibility in real biological samples.

### 7.6. Comparative Insights Across Target Classes

This diversity illustrates how molecular imprinting can address recognition targets spanning a remarkably broad range of template sizes, from sub-nanometre molecules to micrometre-scale cells, using a single, chemically robust materials platform. Selectivity, sensitivity and validation maturity nevertheless differ markedly across these size regimes, so this breadth reflects the versatility of the imprinting concept rather than uniform analytical performance across all target classes.

**Figure 16:**
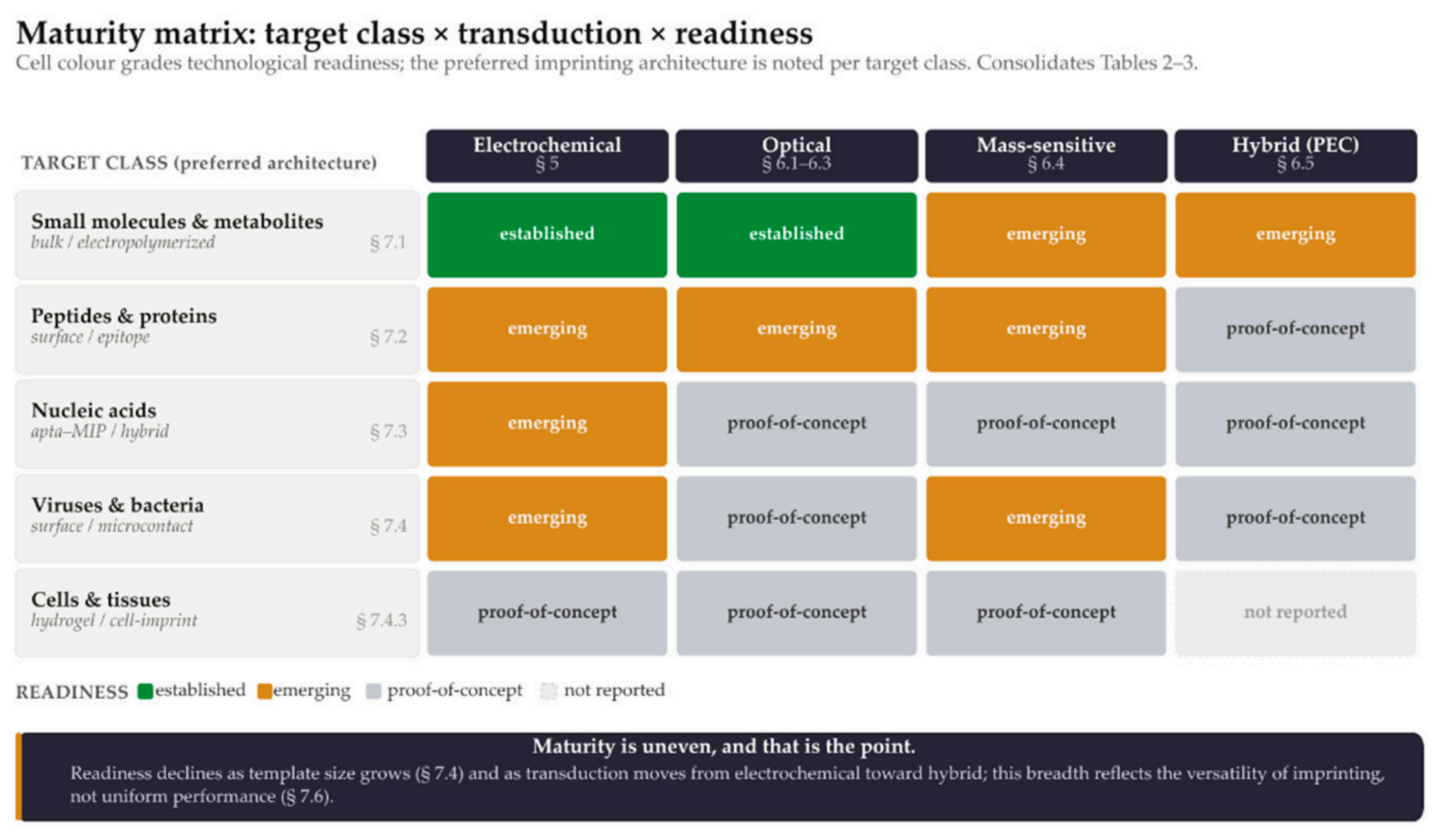
Maturity matrix for MIP biosensors. Cell colour grades technological readiness (established/emerging/proof-of-concept/not reported) for each target class and transduction family; the preferred imprinting architecture is noted per target class. The gradient shows that readiness declines with template size (Section 7.4) and toward hybrid transduction, reflecting the versatility of the imprinting concept rather than uniform performance (Section 7.6). Consolidates Table 2 and the one in Section 8.7.

Figure 16 maps this unevenness explicitly, grading the technological readiness of each target class across the main transduction families.

**Table 2:** Comparative overview of MIP design across target classes. Entries are representative examples from the cited studies, not exhaustive or best-in-class figures; the LOD column reports order-of-magnitude ranges taken from those studies (Section 9.4) and refers to the matrix used in each source (buffer, diluted or real sample), which is not uniform across rows and must be read accordingly. Units necessarily differ by target class—molar concentration for molecules, peptides/proteins and nucleic acids; CFU or PFU for bacteria and viruses; qualitative discrimination for cells—because these analytes are quantified on intrinsically different scales and are not directly comparable across rows.

Target Type	Typical Template Size	Preferred Imprinting Method	Transduction	LOD Range	Representative Applications	Representative Example(s)
Small molecules	<1 nm	Bulk/electropolymerized	Amperometric, EIS	10^−9^–10^−15^ M	Drugs, pesticides	Figure 13 [128]
Peptides/proteins	1–10 nm	Surface/epitope	EIS, SPR, DPV	10^−12^–10^−9^ M	Biomarkers, enzymes	Figure 8 [30,99]
Nucleic acids	2–10 nm	Ionic/hybrid apta-MIP	EIS, FET	10^−12^–10^−9^ M	microRNAs, DNA assays	[137]
Viruses/bacteria	50–1000 nm	Surface/microcontact	QCM, SAW, impedance	~10^3^–10^7^ CFU mL^−1^ (bacteria); ~10^2^–10^5^ PFU mL^−1^ (viruses)	Pathogen detection	Figure 14 [142,144]
Cells/tissues	>1 µm	Hydrogel imprinting	Optical, QCM	qualitative	Cancer cell sensing	[145,146]

### 7.7. Design Challenges and Future Trends

Despite significant progress, imprinting complex biological targets remains challenging. The main limitations concern template stability, template removal, and non-specific adsorption, and they become increasingly severe as the target size grows from small molecules to proteins, viruses, and whole cells; their consequences for reproducibility across laboratories are treated separately in Section 9.1.

Template stability is particularly critical for peptides, proteins, nucleic acids, and cells because polymerization conditions may perturb native conformation, hydration, or surface organization. For this reason, mild aqueous polymerization, surface imprinting, epitope imprinting, and soft hydrogel matrices are increasingly preferred over conventional bulk imprinting when fragile biological templates are involved. Template removal is another key bottleneck: harsh extraction conditions can damage either the template-derived cavity or the polymer biointerface, whereas incomplete removal leads to template bleeding and reduced analytical reliability.

Non-specific binding remains one of the most important barriers to translation into biological fluids. Serum, saliva, sweat, and urine contain proteins, salts, metabolites, and surfactant-like species that can adsorb non-specifically onto polymer surfaces.

Emerging solutions include microfluidic-assisted imprinting, surface-confined polymerisation, and cryopolymerisation for macroporous hydrated matrices, together with the computationally guided monomer selection introduced in Section 2.8 [150,151,152]. What distinguishes the macromolecular and cellular targets considered here is that the templates themselves are poorly captured by the molecular descriptors on which current models are trained; progress therefore depends as much on the standardised datasets discussed in Section 9.7 as on the algorithms.

Whichever target class is addressed, the recognition layer must ultimately be embodied in a device; the formats through which this is achieved are the subject of the next section.

## 8. Integration, Miniaturization, and Wearable MIP Sensors

The translation of MIPs from laboratory prototypes to real-world analytical devices increasingly depends on their integration into miniaturized, flexible, and wearable sensing platforms. The convergence of polymer science, microfabrication, nanostructured electrodes, and soft electronics has created new opportunities to exploit MIPs as selective recognition layers in portable and on-body biosensors for non-invasive monitoring of biochemical signals.

In this context, MIPs are evolving beyond static recognition materials toward engineered interfacial layers that can operate in complex physiological environments, including sweat, saliva, and other biofluids. Wearable MIP sensors must retain selectivity while tolerating mechanical deformation, variable hydration, ionic strength changes, and fluctuating temperature. These requirements place strong constraints on both the imprinted polymer chemistry and the underlying transducer architecture.

### 8.1. From Rigid Electrodes to Flexible Substrates

Early MIP-based biosensors were commonly fabricated on rigid substrates such as glassy carbon electrodes, gold electrodes, silicon chips, or quartz supports. Although these platforms provide excellent electrochemical or optical stability, they are poorly suited to conformal contact with skin, textiles, soft tissues, or curved surfaces. Recent progress in flexible electronics has therefore encouraged the deposition of MIP layers on deformable substrates and nanostructured conductive interfaces [50,153].

Conductive polymers such as PEDOT, polypyrrole (PPy), and polyaniline (PANI) are particularly attractive because they combine electrical conductivity, solution processability, and mechanical compliance. In MIP sensors, these materials can act either as the imprinted recognition matrix itself, especially in electropolymerized films, or as a conductive scaffold supporting a selective MIP layer. The carbon-based materials already discussed as signal-amplifying fillers in Section 4.5 and Section 5.5.1—graphene derivatives, carbon nanotubes, laser-induced graphene, and reduced graphene oxide—acquire an additional role here, since their high surface area and charge-transfer capability are combined with the mechanical compliance that disposable and wearable formats require [50,154,155].

Additional flexible substrates include elastomers, polymer membranes, textile fibers, paper-based supports, and thread- or fiber-based microfluidic structures. These platforms can be combined with MIPs through electropolymerization, drop casting, spin coating, inkjet printing, spray coating, or in situ polymerization. The resulting devices are designed to maintain intimate contact with irregular surfaces while preserving analyte transport toward the imprinted sites [156,157].

The transition from rigid electrodes to flexible substrates is therefore not merely a mechanical adaptation: reliable biochemical readout under realistic wearable conditions depends on jointly controlling adhesion, film thickness, swelling, and electrical stability at the imprinted interface.

### 8.2. Integration with Microfluidic and Lab-on-Chip Platforms

Miniaturization of analytical systems naturally aligns with MIP technology, because both rely on interfacial recognition events that benefit from controlled mass transport and reduced diffusion distances. In microfluidic and lab-on-chip formats, MIP recognition layers can be integrated with miniaturized electrodes, optical windows, or channel walls to enable selective analyte capture under well-defined flow conditions [9].

Microfluidic integration offers several advantages for MIP-based sensors. First, small channel dimensions reduce sample and reagent consumption and shorten the time required for analyte transport to the imprinted sites. Second, controlled flow can improve reproducibility by standardizing incubation, washing, and regeneration steps. Third, patterned microchannels or compartmentalized sensing regions can support multi-analyte detection, provided that different MIP layers are spatially separated to minimize cross-talk between templates and recognition sites.

Electropolymerized MIP films are particularly compatible with lab-on-chip architectures because they can be deposited directly onto microelectrodes or interdigitated electrode arrays. This allows the selective layer to be formed only in the active sensing region, improving device miniaturization and reducing non-specific background. Similar strategies can be applied to optical or hybrid platforms, where MIP microdomains are patterned onto plasmonic, fluorescent, or impedance-sensitive interfaces.

A specific consideration for continuous-flow operation concerns the mechanical and thermal environment to which the recognition layer is subjected. Under sustained flow conditions, wall shear stress and the consequent hydrodynamic drag can promote delamination or gradual erosion of thin printed films, so that adhesion and mechanical anchoring, rather than binding affinity, often determine the service life; conversely, the shear field is exploited constructively in droplet-based microfluidics, where channel geometry and flow rate govern the size of MIP microparticles and yield uniform, accessible binding sites with faster mass transfer than polished monoliths [158]. Channel architecture also influences analyte delivery: serpentine and other tortuous geometries enhance transverse mixing and thin the diffusion boundary layer over the imprinted region, improving mass-transfer efficiency, which often limits rebinding kinetics in laminar microchannels. In formats that impose thermal cycling, for example in chips that integrate nucleic acid amplification, temperature gradients and thermal crosstalk become relevant, and localized monitoring with thin-film resistance temperature detectors (RTDs) or microthermocouples helps to confine them. It should be noted, however, that imprinted cavities are considerably more thermotolerant than protein bioreceptors, so that within the typical operating windows of microfluidics, thermal fluctuation is better regarded as a design constraint to be documented rather than as a demonstrated failure mode of MIP recognition; quantitative studies that isolate its effect on cavity fidelity are still lacking.

Despite these advantages, MIP–microfluidic integration remains technically demanding. Reliable operation requires control over film thickness, adhesion under flow, template removal within confined geometries, antifouling behaviour, and regeneration efficiency. For this reason, current MIP lab-on-chip systems are best described as promising platforms for portable and semi-automated analysis rather than fully mature clinical diagnostic devices [9,159].

### 8.3. Wearable and Epidermal MIP Sensors

Wearable biosensors represent one of the most active directions for the integration of MIPs into real-world sensing platforms. In this context, the MIP layer must retain molecular selectivity under the demanding on-body conditions outlined above, with the added factors of direct skin contact and continuous sweat exposure.

MIP films and nanocomposites can be combined with flexible electrodes, polymer membranes, hydrogels, textiles, or porous substrates to create conformal sensing interfaces. Their main advantage in this setting is that the synthetic recognition layer withstands the mechanical and hydration stresses of on-body use far better than a biological receptor would [5]. However, wearable MIP sensors must also address key limitations, including non-specific adsorption from complex biofluids, mechanical delamination, drift during prolonged use, and calibration variability between individuals. A further, matrix-level constraint is that the concentration measured at the skin or mucosal interface is not a direct surrogate for the circulating (blood) level: sweat rate, evaporative concentration, local pH and ionic strength, surface contamination, and a variable temporal lag relative to plasma all modulate the biofluid-to-blood relationship, so per-subject calibration is often required [160,161]. These physiological factors—examined per biofluid below—currently constrain the clinical interpretability of wearable MIP data as much as transducer performance does.

#### 8.3.1. Sweat-Based MIP Sensors

Sweat is an attractive non-invasive matrix because it contains metabolites, hormones, amino acids, electrolytes, and other small molecules that may reflect physiological or stress-related states. As detailed in Section 8.3, sweat concentrations are not in a fixed proportion to plasma levels; therefore, the reported analytical ranges must be interpreted with respect to sweat-specific reference values rather than to blood-based decision thresholds. MIP-based sweat sensors have therefore been explored for selective recognition of targets such as cortisol, tryptophan, and other low-molecular-weight biomarkers [73,130,162]. In these devices, the MIP is usually integrated with a flexible electrochemical transducer, conducting polymer, hydrogel, or nanostructured electrode to improve charge transfer and maintain function under hydrated conditions.

**Figure 17:**
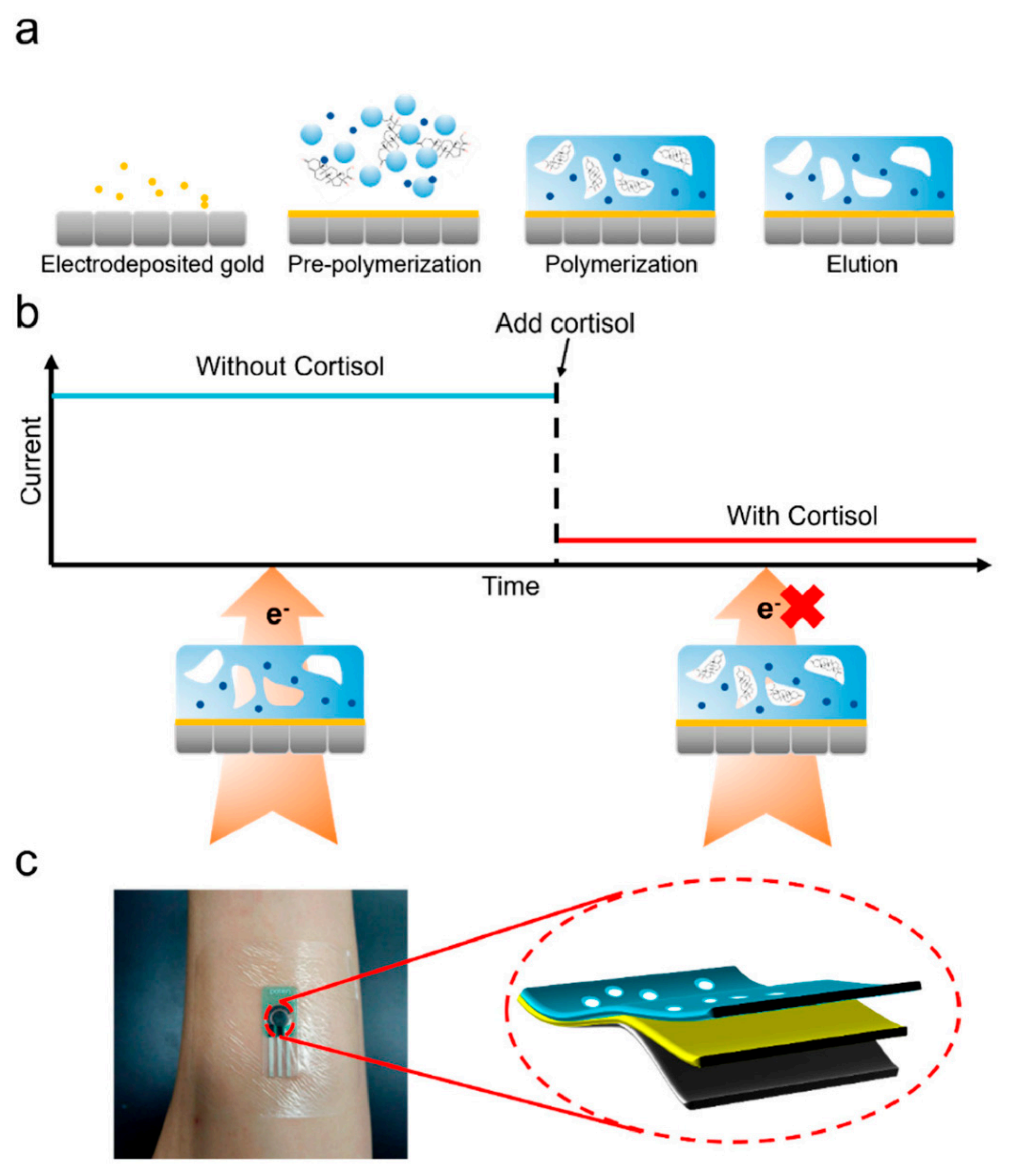
Wearable molecularly imprinted electrochemical sensor for sweat cortisol: (**a**) fabrication sequence combining gold electrodeposition, electropolymerization of the imprinted polypyrrole layer, and template elution; (**b**) the concentration-dependent current change underlying detection; (**c**) the assembled skin-applied device. Reproduced from Liu et al., Nanomaterials 2025, 15(21):1654 [162], under the terms of the Creative Commons CC BY 4.0 license.

For sweat sensing, the most critical design requirements are antifouling behaviour, fast analyte transport, mechanical stability, and stable baseline response during deformation. Hydrophilic or semi-interpenetrating polymer networks can reduce non-specific adsorption and improve compatibility with aqueous sweat, while the conductive scaffolds mentioned above enhance charge transfer and preserve signal stability in the hydrated environment. Nevertheless, very specific performance claims, such as sub-second response, thousands of deformation cycles, or continuous real-time operation, should only be reported when directly supported by the individual device study [73,162]. A representative wearable implementation is shown in Figure 17, where a molecularly imprinted layer electropolymerized on a gold-modified screen-printed electrode is configured as a compact, skin-applied device for sweat cortisol monitoring.

#### 8.3.2. Tear and Saliva Sensing

Tear and saliva are also attractive biofluids for non-invasive monitoring, but their integration with MIP-based wearable devices remains less mature than sweat sensing. Saliva-compatible MIP sensors have been investigated mainly for stress-related biomarkers such as cortisol, often using flexible or thread-like sampling structures coupled to electrochemical readout. These systems illustrate the potential of combining sample collection, capillary transport, and selective MIP recognition in compact formats [163].

By contrast, MIP-coated contact lenses or mouthguard-type devices for continuous biochemical monitoring should currently be presented as future opportunities rather than established MIP platforms, unless supported by specific experimental evidence [164]. Transparent conductive electrodes, soft substrates, biocompatible coatings, and wireless electronics are important enabling technologies, but their successful integration with MIP recognition layers still requires further validation.

Overall, wearable and microfluidic MIP sensors offer a promising route toward portable, non-invasive, and personalized biochemical monitoring. Their translation will depend on robust polymer adhesion, antifouling interfaces, reproducible fabrication, stable calibration, and validation in real biofluids under realistic mechanical and environmental conditions. Clinical validation in the strict sense requires a further level of evidence that most reported devices do not yet provide: adequately powered sample sizes, comparison against an accepted reference method, reporting of diagnostic sensitivity and specificity with ROC/AUC analysis, and, ideally, blinded or external validation (Section 9.3). Until such evidence is available, on-body MIP demonstrations are most accurately described as clinically oriented proof-of-concept studies rather than as validated diagnostic devices.

### 8.4. Printing and Additive Manufacturing of MIP Devices

Printing and additive manufacturing are increasingly relevant to the scalable fabrication of MIP-based sensing platforms, although their maturity varies substantially depending on the specific process and device architecture. Among printing technologies, screen printing is currently the most established route for producing disposable electrochemical MIP sensors [159,165,166]. In these devices, the conductive electrode pattern is typically fabricated by screen printing, whereas the MIP layer is subsequently deposited by electropolymerization, drop casting, spin coating, or in situ polymerization on the active working electrode.

The advantages of screen-printed electrodes for MIP sensing—low cost, small sample volume, compatibility with portable potentiostats, and straightforward modification of the working electrode—have already been noted in Section 3.2.2 and Section 5.2.2; what is relevant here is that the same features make screen printing the only patterning route currently compatible with volume manufacture. These features make screen-printed MIP electrodes particularly suitable for decentralized analysis, environmental monitoring, food control, and point-of-care-oriented testing [156,166].

Inkjet printing, aerosol jet printing, and other digital patterning methods are attractive because they offer high spatial resolution, mask-free deposition, and compatibility with flexible substrates. However, their use for directly printing fully functional MIP recognition layers remains less mature than screen printing. Key challenges include ink formulation, control of polymerization within printed droplets, template removal, film adhesion, and preservation of binding-site fidelity after drying or curing. Until these challenges are resolved, digital printing of the MIP layer remains a developing route, whereas volume manufacture still rests on screen printing [155].

Three-dimensional printing adds a further level of integration by enabling customized housings, flow cells, microneedles, microfluidic structures, and sensor supports. In the MIP field, 3D printing is especially useful for fabricating device architectures that can host or interface with imprinted recognition zones, rather than necessarily printing the MIP chemistry itself. More advanced approaches, including direct printing of porous imprinted polymers or MIP-containing resins, are emerging but still require systematic validation in terms of selectivity, template extraction, reproducibility, and compatibility with electronic readout.

**Figure 18:**
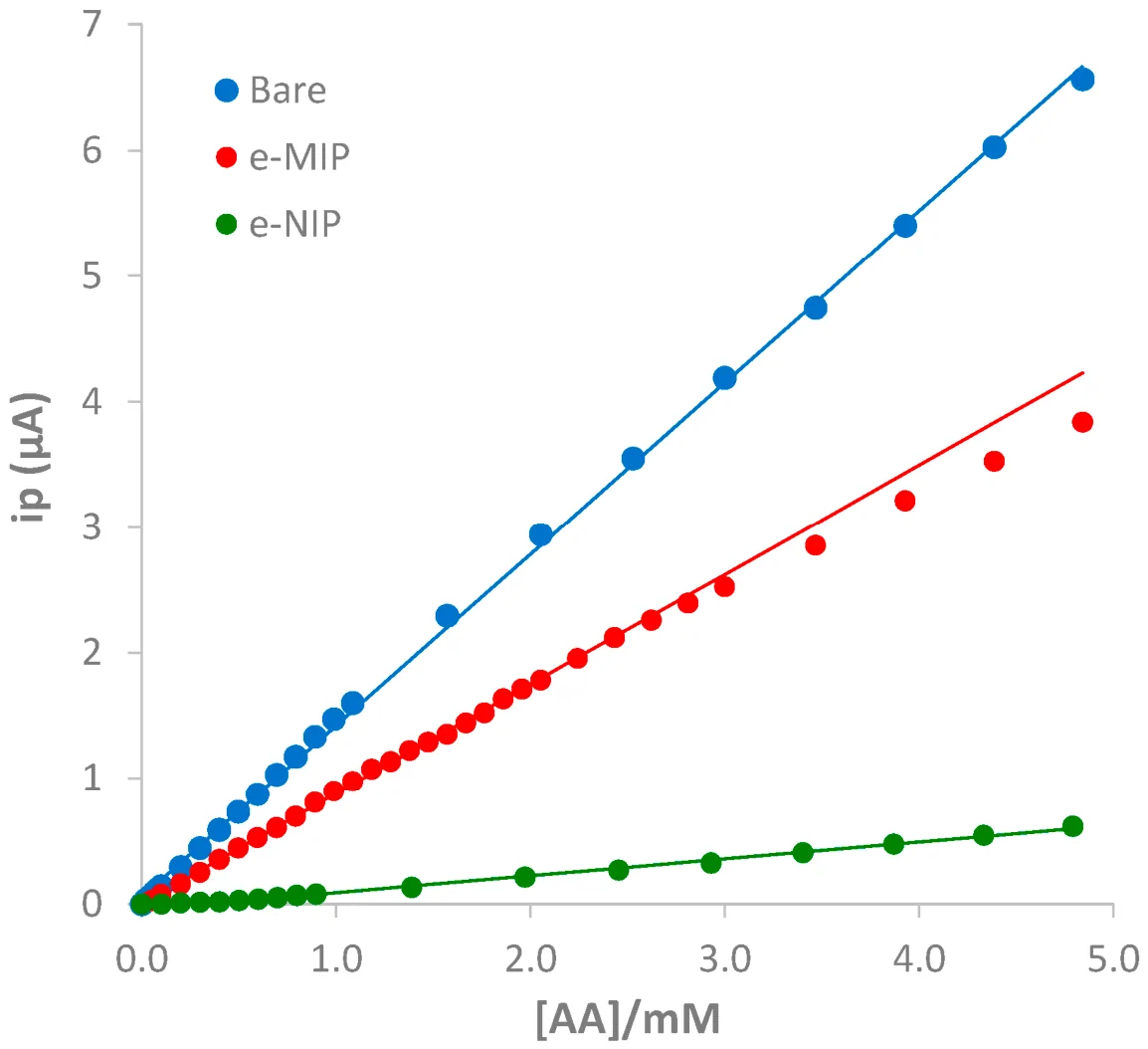
Disposable screen-printed MIP sensor: an electrosynthesized molecularly imprinted polypyrrole film (e-MIP) is deposited on the graphite working electrode of a screen-printed cell and used for differential-pulse-voltammetric detection, with selectivity assessed against bare and non-imprinted electrodes. This exemplifies screen printing as the most established route to low-cost, disposable MIP sensors. Reproduced from Alberti et al., Chemosensors 2023, 11(6):348 [165], under the terms of the Creative Commons CC BY 4.0 license.

Across these methods a clear maturity gradient emerges: screen printing already supports practical disposable devices, whereas inkjet, aerosol-jet, and direct 3D printing of the MIP remain developing strategies for future high-throughput and customizable platforms. Figure 18 illustrates this mature manufacturing route, in which an imprinted polypyrrole film is electrosynthesized directly on the graphite working electrode of a disposable screen-printed cell and read out by differential-pulse voltammetry.

### 8.5. Wireless and Self-Powered MIP Systems

The integration of MIP sensors with wireless electronics and autonomous power sources is an important future direction for continuous and decentralized monitoring. In principle, MIP-based recognition layers can be paired with compact potentiostats and wireless modules (BLE, NFC, or RFID) or smartphone-assisted readout to transmit signals without benchtop instrumentation.

#### 8.5.1. Wireless Communication

Wireless communication is already well established in the broader field of wearable electrochemical biosensors, but its specific combination with MIP recognition layers remains less mature. Most current MIP wearable or portable devices still rely on external potentiostats, wired connections, or laboratory readout systems during validation. Wireless MIP sensors thus sit at the threshold of translation: the enabling wireless electronics are mature, but their combination with imprinted recognition layers has yet to be broadly demonstrated [161,167].

For MIP systems, the main integration challenges are not limited to signal transmission. The recognition layer must maintain selectivity under mechanical deformation and changing hydration, while the electronics must compensate for drift, temperature variations, motion artifacts, and matrix effects. Wireless operation therefore requires co-design of the MIP interface, the transducer, the sampling architecture, and the signal-processing module.

#### 8.5.2. Self-Powered MIP Sensors

Self-powered sensing based on triboelectric nanogenerators, piezoelectric harvesters, biofuel cells, or thermoelectric devices is an active area in wearable electronics. However, direct examples in which a molecularly imprinted recognition layer is fully integrated with a triboelectric or piezoelectric energy-harvesting unit to form an autonomous MIP biosensor remain relatively few [168,169]. For this reason, self-powered MIP systems are best read as a proof-of-concept direction, not a consolidated device class.

A realistic near-term strategy is to integrate MIP-based selective recognition with low-power electronics and externally validated energy-harvesting modules. In such architectures, the MIP provides chemical selectivity, while the harvester supplies power for signal acquisition, conditioning, or wireless transmission. Future progress will depend on reducing sensor power consumption, improving baseline stability, minimizing fouling, and demonstrating reliable operation in real biofluids under realistic mechanical conditions.

The two cases should therefore be kept distinct: pairing MIPs with portable, miniaturised, and wireless read-out is already realistic, whereas fully self-powered MIP biosensors still await convincing experimental demonstration.

### 8.6. Stability, Regeneration, and Biocompatibility in Wearable Environments

Operation in wearable environments imposes stringent requirements on MIP-based sensors. Unlike measurements performed under controlled laboratory conditions, wearable devices must retain selectivity, signal stability, and mechanical integrity under variable hydration, sweat composition, temperature, motion, and prolonged contact with biological interfaces. These conditions can affect both the imprinted recognition layer and the underlying transducer, leading to baseline drift, non-specific adsorption, swelling, delamination, or gradual loss of binding-site fidelity.

The rigidity–accessibility trade-off of Section 2.3 and Section 4.2.1 acquires a mechanical dimension in wearable formats: highly crosslinked MIPs preserve cavity geometry and chemical robustness, but the same rigidity compromises adhesion to deformable substrates and tolerance to bending or stretching. Conversely, softer hydrogel-like MIPs offer improved compatibility with aqueous and deformable environments but may suffer from swelling-induced changes in binding-site geometry. Crosslinking density, film thickness, polymer–substrate adhesion, and hydration behaviour therefore need to be optimized together rather than independently.

Biofouling is another major limitation in sweat, saliva, interstitial fluid, and other complex biofluids: the non-specific adsorption already noted in Section 7.7 here masks the imprinted sites and reduces selectivity, with the added contribution of skin-derived contaminants under epidermal contact. Here the antifouling monomer chemistries introduced earlier become essential to limit non-specific adsorption while preserving analyte access [73,160]. Protective permeable layers, such as hydrogel coatings or ion-selective polymer membranes, can further improve operational stability by regulating analyte transport and limiting direct exposure of the sensing interface to interfering species.

Regeneration is essential for reusable MIP sensors, but its implementation in wearable formats remains challenging. The regeneration routes established for laboratory electrochemical devices in Section 5.6—solvent washing, pH or ionic-strength modulation, and electrochemical conditioning—must here be rendered mild, compatible with skin contact, and integrated with small-volume sampling architectures. Potential strategies include controlled rinsing in microfluidic channels, reversible electrochemical cleaning, or the use of weakly bound templates that can be displaced under physiological conditions. However, regeneration protocols must be validated carefully, because repeated cycling can alter film morphology, remove loosely bound polymer domains, or progressively reduce binding-site fidelity.

Biocompatibility is equally important for epidermal and body-fluid-contacting devices. Although MIPs are generally more chemically stable than biological receptors, residual template, unreacted monomers, initiators, solvents, or degradation products may affect safety and analytical reliability. Therefore, template extraction, polymer purification, cytocompatibility, and leachable assessment should be considered when MIPs are intended for prolonged contact with skin, mucosa, or biological fluids.

Finally, biodegradable and transient MIP platforms could reduce the environmental footprint of single-use wearable sensors [170]. Because the same degradability-versus-stability trade-off governs every disposable format, these systems are treated in full in Section 10.5, and, like their laboratory counterparts, remain promising design directions rather than established wearable technologies.

Overall, stability, regeneration, and biocompatibility are not secondary engineering details but central determinants of wearable MIP sensor performance. Successful translation will require simultaneous optimization of polymer chemistry, antifouling interface design, mechanical adhesion, sampling strategy, and long-term calibration stability under realistic operating conditions.

### 8.7. Benchmarking Performance and Design Parameters

Table 3 summarises representative wearable and miniaturised MIP platforms reported to date, together with their substrate, target biofluid, and integration route. The values quoted are those reported in the underlying studies and, for the reasons set out in Section 9.4, should be read as indicative rather than as benchmarked figures.

Taken together, these platforms illustrate the direction in which MIP technology is moving—toward continuous, personalised monitoring aligned with digital health—but largely at the proof-of-concept level: few combine a wireless or self-powered read-out with prolonged in vivo or on-body validation, and the wireless/self-powered entries in the last column should be read as integration routes demonstrated in the broader wearable literature rather than as fully validated MIP devices (Section 8.5 and Section 8.6). Their heterogeneity, and the fact that several entries are projected or proof-of-concept, also makes plain why the figures in Table 3 cannot yet be compared directly—the question of reliability and standardisation to which the next section is devoted.

## 9. Reliability, Standardization, and Benchmarking in MIP Biosensing

The rapid expansion of molecularly imprinted polymer (MIP)-based biosensing over the past decade has generated a broad and heterogeneous body of literature spanning polymer chemistry, analytical chemistry, nanomaterials, and device engineering. However, this diversity has also created a central challenge: ensuring reproducibility, comparability, and traceability across studies that employ different imprinting protocols, transducer architectures, target matrices, and signal-processing methods.

Without systematic benchmarking, the translation of MIP sensors from proof-of-concept demonstrations to validated analytical or diagnostic devices remains difficult. Reported performances are often difficult to compare because limits of detection, linear ranges, selectivity factors, regeneration efficiency, and stability are not always measured under equivalent conditions. As MIP-based sensors mature toward practical applications, the field increasingly requires standardized reporting practices, quantitative metrology, and validation procedures analogous to those used in established electrochemical sensors, immunoassays, and point-of-care diagnostic platforms [173,174].

**Figure 19:**
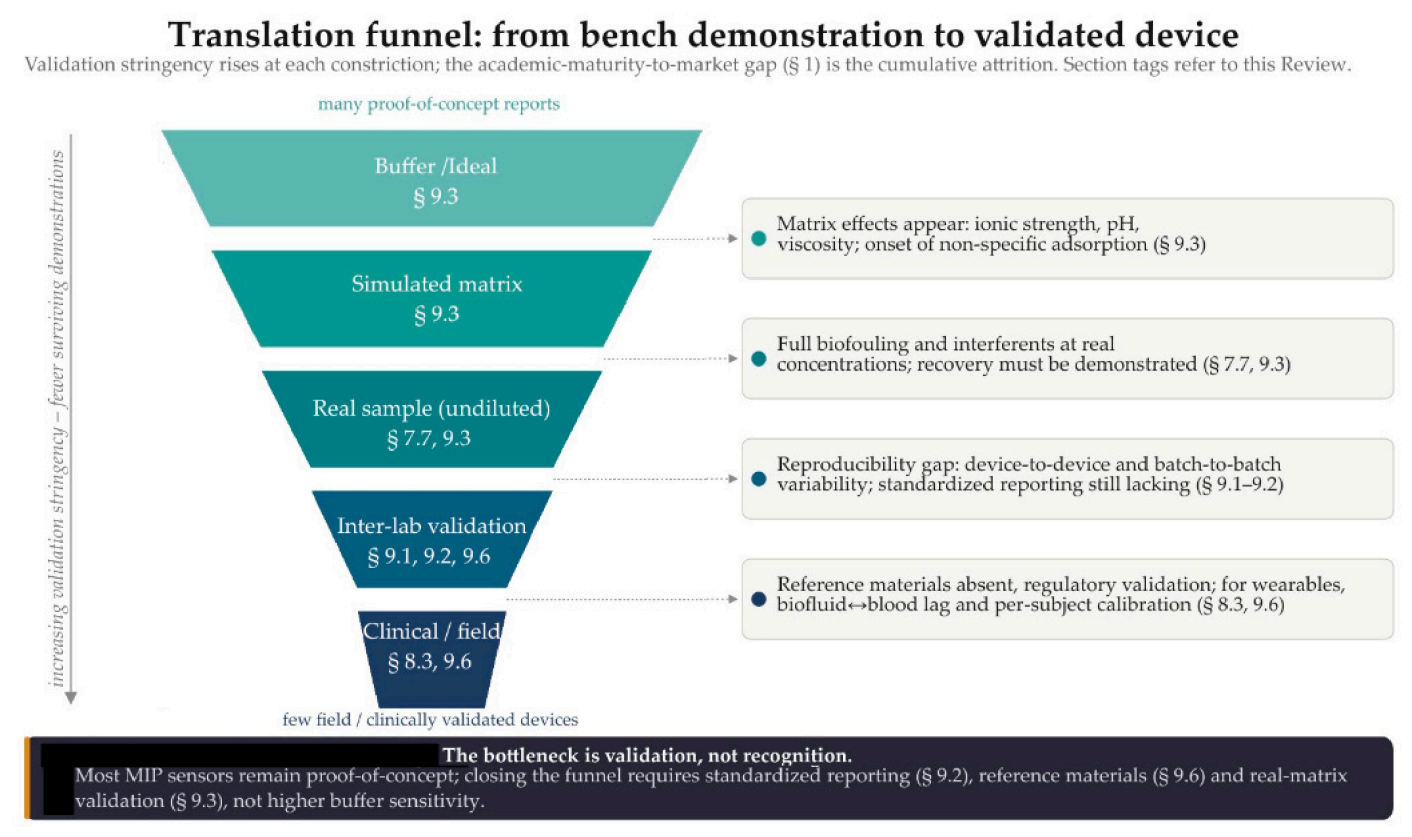
Translation funnel for MIP biosensors. Analytical validation becomes progressively more stringent from buffer to clinical/field use; the attrition driver at each constriction is annotated, with the corresponding Sections. Most reported platforms remain proof-of-concept, and closing the funnel requires standardized reporting (Section 9.2), reference materials (Section 9.6), and real-matrix validation (Section 9.3) rather than higher buffer sensitivity.

Figure 19 frames this translation as a funnel in which the number of surviving demonstrations falls at each step—from buffer to simulated matrix, real sample, inter-laboratory, and field/clinical validation—and identifies the dominant attrition driver at each constriction.

### 9.1. The Reproducibility Gap

Although MIP synthesis can, in principle, be highly reproducible when reaction parameters are strictly controlled, the analytical performance of MIP-based sensors is often sensitive to subtle variations in fabrication and measurement conditions. Small changes in the synthesis and processing parameters detailed below can significantly alter binding-site density, affinity distribution, charge-transfer properties, and mass transport [175,176].

This variability is further amplified by differences in template extraction, residual template content, polymer swelling, surface morphology, and measurement environment. For example, buffer composition, ionic strength, pH, temperature, incubation time, washing protocol, and matrix composition can all influence the apparent binding response. As a result, two nominally similar MIP sensors may display different calibration slopes, background signals, recovery values, or selectivity patterns [97,177].

These sources of irreproducibility act along the entire fabrication-to-measurement chain. At the synthesis stage, variability in monomer–template stoichiometry and in polymerization kinetics, compounded by incomplete or non-standardized template extraction, generates a heterogeneous distribution of binding sites and affinity classes, while nanomaterial-based MIP composites additionally suffer from batch-to-batch differences in composition. At the fabrication stage, uncontrolled film thickness, roughness, porosity, swelling, or microcracking—together with differences in electrode cleaning, activation, and pretreatment—alter the geometry and accessibility of the recognition layer. Finally, at the measurement and data-analysis stage, inconsistent calibration, baseline correction, and signal-normalization procedures, as well as the use of different matrices, interferent concentrations, and recovery protocols, further distort the reported response.

These factors can lead to substantial device-to-device or batch-to-batch deviations in analytical response, sometimes large enough to compromise reliable comparison between studies or practical validation. Such variability is especially problematic for clinical, wearable, environmental, or regulatory applications, where analytical performance must be demonstrated not only in optimized buffer systems but also in complex real samples using reproducible and transparent protocols [116,178].

### 9.2. Toward Standardized Synthesis and Reporting

To address reproducibility challenges, the MIP biosensing community increasingly needs harmonized reporting practices and broadly accepted best-practice criteria. At present, many studies report excellent analytical performance, but the underlying synthesis, processing, and validation conditions are not always described in sufficient detail to enable direct reproduction or meaningful comparison across laboratories [176,179].

A robust reporting framework for MIP-based sensors should include, at minimum, the following descriptors:

1. **Synthesis parameters:** template identity and purity, monomer/crosslinker ratio, solvent or porogen composition, initiator type and concentration, polymerization method, temperature, reaction time, and template removal protocol.
2. **Characterization of the pre-polymerization complex**: where possible, evidence of the stoichiometry and binding state of the monomer–template assembly prior to polymerization, for example via ^1^H-NMR titration and Job-plot analysis, UV-vis titration, or electrospray ionization mass spectrometry (ESI-MS) and MALDI-TOF of the complex, since the template–monomer arrangement fixed at this stage governs the fidelity and homogeneity of the resulting cavities [180,181,182].
3. **Morphological and compositional characterization:** SEM or TEM for morphology, FTIR or Raman spectroscopy for chemical structure, XPS or elemental analysis for surface composition, BET analysis for porosity when relevant, and contact-angle measurements for wettability and interfacial hydrophilicity.
4. **Binding characterization:** IF, binding capacity, affinity parameters such as apparent dissociation constants, adsorption isotherms, binding kinetics, and comparison with non-imprinted controls prepared under identical conditions [97,183].
5. **Device integration details:** substrate material, transducer geometry, electrode or optical interface, MIP film thickness, deposition method, active sensing area, nanomaterial loading, and conditioning procedure before measurement.
6. **Analytical performance metrics:** limit of detection (LOD), limit of quantification (LOQ), sensitivity, linear range, response time, selectivity against structurally related interferents, recovery in real samples, reusability, and device-to-device reproducibility.
7. **Stability testing:** signal retention over repeated binding/regeneration cycles, short- and long-term storage stability, resistance to humidity, temperature, pH, ionic strength variation, and performance under relevant matrix conditions.

**Figure 20:**
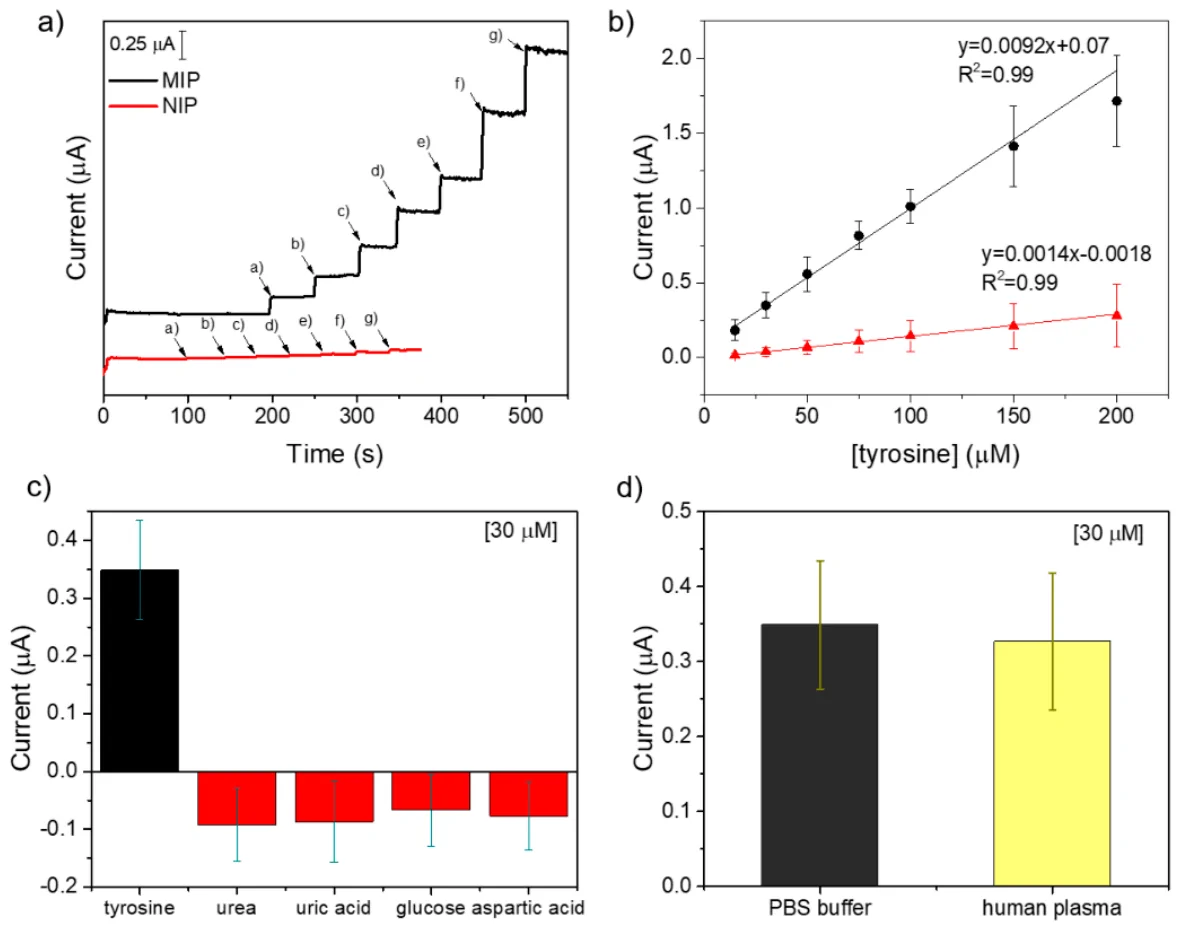
Example of comprehensive analytical characterization recommended for standardized reporting of MIP sensors. (**a**) Amperometric response of the imprinted polymer (MIP) to increasing analyte concentrations (a = 15 μM, b = 30 μM, c = 50 μM, d = 75 μM, e = 100 μM, f = 150 μM, g = 200 μM) compared with the NIP, used to quantify the IF; (**b**) calibration curve of the MIP response; (**c**) selectivity test against structurally related interfering molecules; (**d**) analyte detection in spiked human plasma, illustrating recovery in a real matrix. Reproduced from Gagliani et al., Molecules 2024, 29, 1632 [97], under the terms of the Creative Commons CC BY 4.0 license.

As an illustration of these reporting descriptors, Figure 20 shows a complete material- and device-level characterization of a representative MIP sensor, in which the IF, calibration behaviour, selectivity against interferents, and recovery in a real matrix are all reported alongside the non-imprinted control.

Particular attention should be given to the distinction between material-level and device-level metrics. For example, a high IF measured in batch rebinding experiments does not necessarily translate into high analytical sensitivity in an integrated sensor, where mass transport, film morphology, charge transfer, and fouling may dominate the response [170,177]. Similarly, low detection limits obtained in buffer should not be presented as equivalent to performance in real matrices; the underlying matrix-effect mechanisms and the validation they require are detailed in Section 9.3.

Standardized descriptors would enable more reliable comparison of MIP sensors across studies and facilitate the aggregation of performance data into searchable databases. Such databases could support meta-analysis, machine-learning-assisted MIP design, and rational selection of monomer–template systems [150,184]. Ultimately, transparent reporting is a prerequisite for the credible comparison and validation of MIP biosensors.

### 9.3. Metrological Framework: From Sensing to Quantification

A major step toward standardization is the establishment of a metrological framework that links MIP sensor responses to quantifiable analyte concentrations with defined accuracy, precision, and uncertainty. Many MIP sensors report responses as arbitrary signal changes, such as ΔI, ΔR_ct, Δf, Δλ, or normalized fluorescence intensity. While these quantities are useful for demonstrating recognition, they do not by themselves guarantee analytical traceability or comparability between devices [173].

For MIP biosensors to become reliable quantitative tools, calibration must be performed using well-defined standards, appropriate blank controls, and statistically robust models. Calibration curves should be generated over a relevant concentration range, including the expected physiological, environmental, or regulatory decision thresholds. The limit of blank, limit of detection, and limit of quantification should be calculated using transparent statistical criteria rather than only visual extrapolation or signal-to-noise approximations [185].

Matrix effects are especially important. A sensor calibrated in buffer may respond differently in real biological, food, or environmental matrices because non-specific adsorption, ionic strength, pH, viscosity, and competing molecules can alter both binding and signal transduction. Therefore, validation in real or realistically simulated matrices should include recovery studies, comparison with reference analytical methods when available, and evaluation of potential interferents at relevant concentrations [178,186].

Beyond the calibration and matrix-validation requirements already stated, quantitative comparability rests on three further elements. Calibration should, wherever possible, be anchored to certified or well-characterized reference standards and complemented by non-imprinted controls and matrix-matched calibration; precision should be reported explicitly as replicate measurements with confidence intervals, standard deviations, and relative standard deviations; and the response model should be documented through dynamic range, linearity, saturation behaviour, and fitting parameters. Two dimensions specific to MIP devices deserve particular emphasis: the reversibility of recognition—recovery, hysteresis, desorption efficiency, and regeneration stability—and the several levels of variability, device-to-device, batch-to-batch, and day-to-day, that ultimately determine whether reported figures are transferable between laboratories.

For clinical translation, MIP sensors will eventually need to align with broader diagnostic validation frameworks, including concepts embedded in standards for medical laboratories and assay evaluation, such as accuracy, precision, uncertainty, reference intervals, method comparison and—where a diagnostic decision is intended—diagnostic sensitivity and specificity established on adequately powered cohorts, with ROC/AUC analysis and, ideally, blinded or external validation [116,174]. For wearable formats specifically, this framework must additionally accommodate the biofluid-to-blood relationship and per-subject calibration discussed in Section 8.3. Similarly, environmental applications will require validation against regulatory concentration limits and established reference methods. MIP-specific standards are not yet fully established, but adopting rigorous metrological practices from mature analytical technologies will accelerate acceptance of MIP biosensors in clinical diagnostics, food safety, environmental monitoring, and wearable health assessment [123,187].

### 9.4. Benchmarking Analytical Performance: The Need for Unified Metrics

While thousands of MIP sensors have been reported, meaningful comparison is often hampered by inconsistent performance descriptors. To facilitate benchmarking, the community is increasingly adopting a unified set of figures of merit, as summarized below Table 4.

Establishing such benchmarks would enable comparative performance evaluation, allowing rapid identification of top-performing materials and architectures [188,189]. These values represent typical or aspirational figures reported in the literature, however, rather than formally established, community-wide standards; in particular, sub-nanomolar or femtomolar detection limits and cross-reactivity below 10% are indicative targets whose achievability depends strongly on the analyte, matrix, and transduction platform.

### 9.5. Reliability and Long-Term Stability

For MIP biosensors to be adopted in practical analytical settings, they must demonstrate long-term reliability under conditions that resemble their intended use. This requirement is particularly important for wearable, environmental, food-safety, and point-of-care applications, where sensors may be exposed to temperature variations, humidity, mechanical stress, complex matrices, and prolonged storage before use.

Stability testing should therefore extend beyond short-term repeatability in buffer solutions. Relevant evaluations include thermal cycling, humidity exposure, operation in simulated or real biological fluids, mechanical deformation for flexible devices, chemical resistance to interferents or cleaning solutions, and shelf-life aging under defined storage conditions [130,190]. For wearable or flexible MIP sensors, bending, stretching, compression, and repeated hydration/dehydration cycles should be assessed together with analytical response, rather than treated as separate mechanical tests.

**Figure 21:**
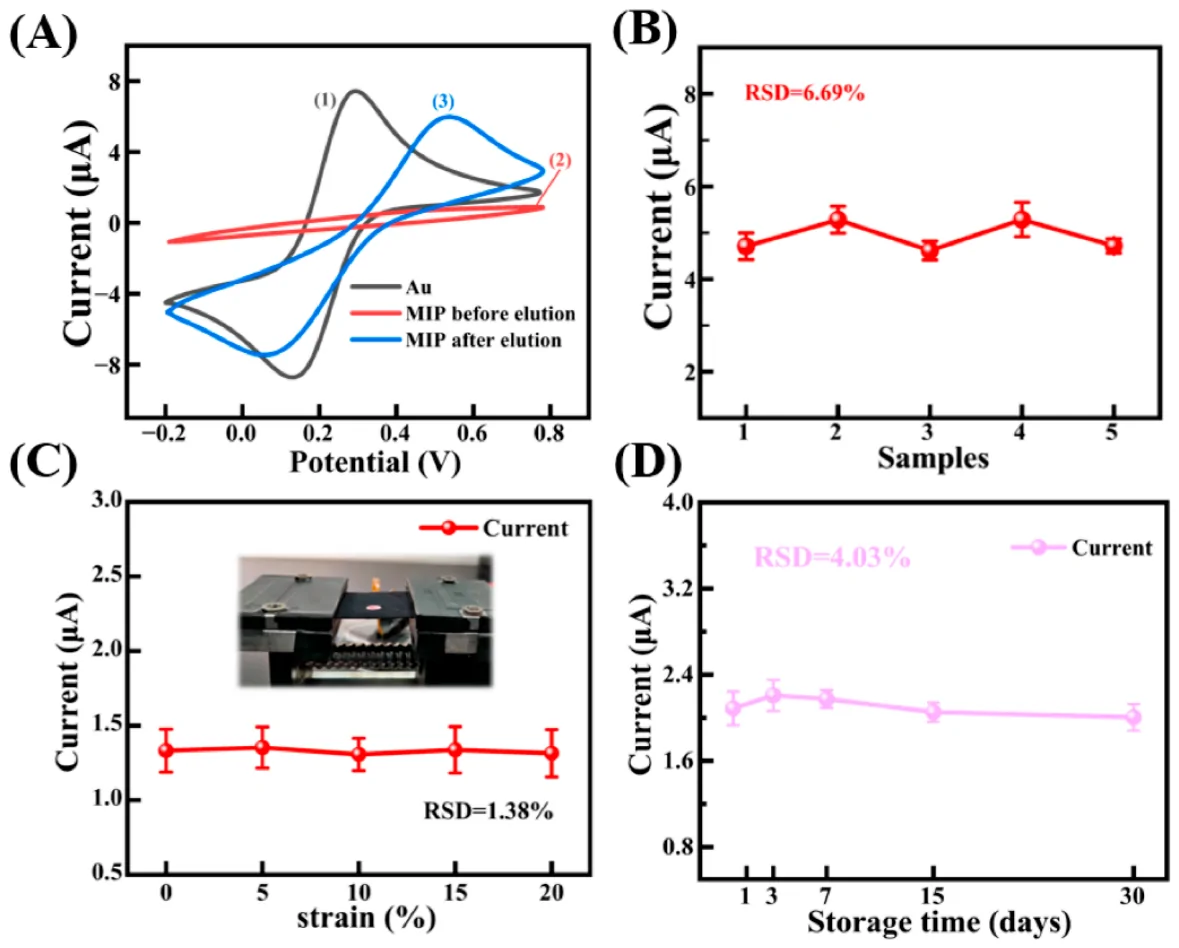
Example of multi-parameter reliability and stability testing for a wearable MIP sensor. (**A**) Cyclic voltammograms recorded at successive stages of electrode preparation; (**B**) fabrication reproducibility across independently prepared electrodes (RSD = 6.69%); (**C**) current response under repeated tensile strain (RSD = 1.38% at fixed analyte concentration); (**D**) long-term storage stability, with 96.1% of the initial signal retained after 30 days. Reproduced from Chen et al., Biosensors 2025, 15, 194 [130], under the terms of the Creative Commons CC BY 4.0 license.

Figure 21 illustrates the type of multi-parameter stability assessment advocated here, combining fabrication reproducibility across independently prepared electrodes, response under mechanical strain, and signal retention over one month of storage for a wearable MIP sensor.

Long-term reliability depends on both the imprinted polymer and the complete device architecture. Excessive swelling, polymer cracking, delamination, fouling, electrode degradation, and incomplete regeneration can all contribute to signal drift. The protective and stabilizing strategies that mitigate these effects—optimized crosslinking density, antifouling interfaces, permeable protective membranes, and compliant encapsulation—are those detailed in Section 5.6 and Section 8.6 [123,174]. However, such coatings must be designed carefully, because they may also slow analyte diffusion, alter response time, or introduce additional mass-transport barriers.

Regeneration stability is another key parameter. A sensor may show high initial sensitivity but progressively lose performance after repeated binding and washing cycles. Therefore, reusability should be reported not only as the number of successful cycles but also as signal retention, baseline recovery, variation in calibration slope, and changes in selectivity after repeated operation [130]. Accelerated aging protocols may provide useful indications of operational lifetime, but they should be interpreted cautiously unless correlated with real-time storage and use-condition studies.

Long-term stability should therefore be treated as an integral analytical performance metric, comparable in importance to sensitivity or detection limit. A MIP biosensor intended for practical use should demonstrate stable response, reproducible calibration, and preserved selectivity over the time scale relevant to the intended application [190].

### 9.6. Reference Materials and Inter-Laboratory Validation

The establishment of reference materials and inter-laboratory validation protocols would represent an important step toward reliable MIP biosensor development. In principle, well-characterized reference MIP materials with defined composition, morphology, binding capacity, and affinity distribution could be used to compare synthesis protocols, template-removal procedures, and analytical performance across laboratories [170,179]. However, such reference systems are not yet broadly established for MIP biosensing.

A practical first step would be the definition of benchmark template–monomer–crosslinker systems for representative target classes, including small molecules, proteins, and complex biological matrices. These benchmark systems should be accompanied by standardized procedures for synthesis, template extraction, rebinding assays, non-imprinted controls, and sensor calibration. Inter-laboratory testing could then assess variability in binding capacity, IF, response slope, limit of detection, recovery, regeneration efficiency, and stability [186].

Reference materials in MIP biosensing would not need to replicate the role of antibodies or certified reference materials directly. Rather, they would serve as reproducible polymeric benchmarks for assessing fabrication quality, binding-site accessibility, and device-level response. Such benchmarks would be especially useful for distinguishing variability arising from the polymer synthesis from variability introduced by the transducer, the matrix, or the measurement protocol [97].

Collaborative inter-laboratory studies would also help identify which metrics are most robust and which are highly protocol-dependent. This is essential for moving from isolated proof-of-concept demonstrations toward validated analytical platforms. Inter-laboratory standardization therefore remains a priority to be built collaboratively, rather than a resource the field can currently draw upon [179].

### 9.7. Data Transparency and Digital Benchmarking

Data transparency is essential for improving reproducibility and accelerating rational MIP design. In line with FAIR data principles, MIP biosensing would benefit from structured digital repositories containing standardized information on template identity, molecular descriptors, monomer and crosslinker composition, polymerization conditions, template-removal protocols, morphology, binding parameters, transduction modality, and analytical performance [184].

Such repositories should also include negative or low-performance results, because these data are highly informative for understanding which monomer–template combinations, solvents, or device architectures fail to produce selective recognition. At present, the literature is biased toward successful demonstrations, which limits the ability to build predictive models and may overestimate the generality of high-performance MIP sensors [173].

The design applications of ML outlined in Section 2.8 are ultimately limited not by the algorithms but by the data: monomer selection, composition optimisation, and performance prediction all presuppose large, curated, and comparably reported datasets. Without standardised descriptors and consistent reporting, such models risk learning publication-specific biases rather than transferable structure–property–performance relationships [150,191].

Digital benchmarking should therefore be considered a long-term enabling infrastructure for the field. Its value would lie not only in ranking sensor performance but also in identifying design rules, failure modes, and reproducibility bottlenecks across MIP chemistries and transduction platforms [191,192].

### 9.8. Proposed Framework for Standardization

The requirements set out in Section 9.2, Section 9.3, Section 9.4, Section 9.5, Section 9.6 and Section 9.7 can be arranged into a four-level framework connecting polymer synthesis, device integration, analytical validation, and data reporting; the following paragraphs assign each descriptor to the level at which it should be controlled, rather than introducing further requirements.

At the material level, standardization should focus on reproducible synthesis, template removal, cavity formation, chemical composition, morphology, porosity, wettability, binding capacity, affinity distribution, and comparison with non-imprinted controls. These descriptors define whether the MIP material itself is well characterized [183,193].

At the device level, standardized protocols should describe transducer geometry, active area, film thickness, deposition method, conditioning procedure, calibration strategy, signal normalization, response time, reusability, and device-to-device variability. This level is essential because a well-performing MIP material does not automatically translate into a reliable integrated sensor [177].

At the system level, validation should be performed under application-relevant conditions, including complex biological fluids, food or environmental matrices, flow operation, mechanical deformation for wearable devices, temperature and humidity variation, and long-term storage. Recovery studies, interference testing, regeneration analysis, and comparison with reference methods should be included whenever possible [178,187].

At the data level, open and interoperable reporting formats should link synthesis parameters, material properties, transduction modality, and analytical figures of merit. This would enable meta-analysis, database construction, and eventual machine-learning-assisted optimization of MIP sensor design [184,191].

Such a hierarchical framework would provide traceability from polymer synthesis to in-field operation. Although MIP-specific certification pathways are not yet fully established, adopting structured reporting, rigorous validation, and transparent data practices would bring MIP biosensing closer to the standards expected for clinically, environmentally, and industrially relevant analytical technologies [123,174].

Standardisation, however, is only one of the constraints that will govern the deployment of MIP biosensors at scale; the second, and increasingly binding, constraint is environmental, and it is examined next.

## 10. Sustainability and Green Design in MIP Biosensing

As MIP-based biosensing moves from laboratory prototypes toward practical analytical devices, sustainability is becoming an increasingly important design criterion. MIPs have traditionally been synthesized using petrochemical monomers, high crosslinker contents, organic solvents, and non-degradable matrices. While these formulations often provide excellent chemical stability and binding-site rigidity, they are not always aligned with the principles of green chemistry, circular materials design, or environmentally responsible device manufacturing.

The next generation of MIP biosensors must therefore balance analytical performance against several constraints that are frequently, but incorrectly, treated as equivalent: environmental impact (resource and energy use, waste, end-of-life fate), process safety (safer synthesis, lower reagent consumption), and biological safety (biocompatibility and low leachable toxicity). These properties are logically independent—a bio-based matrix need not be biodegradable, a biodegradable one need not be biocompatible, and none of them is automatically non-toxic, recyclable, or environmentally benign—so each must be substantiated by its own evidence rather than inferred from another. This is particularly relevant for disposable sensors, wearable devices, food-safety strips, and environmental monitoring platforms, where large-scale deployment could generate significant material waste. Sustainable MIP design should not be viewed as a replacement for performance optimization, but as an additional constraint that must be integrated into polymer chemistry, device fabrication, and lifecycle assessment.

### 10.1. Green Chemistry Principles in MIP Synthesis

The Twelve Principles of Green Chemistry provide a useful conceptual framework for rethinking MIP synthesis [194]. In the context of molecular imprinting, sustainability can be addressed at multiple levels, including monomer selection, solvent choice, polymerization method, template extraction, device fabrication, and end-of-life disposal.

One important direction is the use of renewable or lower-impact feedstocks. Bio-based monomers—itaconic and fumaric acids, levulinic acid derivatives, tannin- and lignin-derived aromatics, and other biomass-derived vinyl compounds, several of which were introduced in Section 4.6—may partially replace fossil-derived monomers in selected formulations. However, the use of bio-based precursors alone does not guarantee sustainability; their purity, reactivity, toxicity, availability, and ability to generate selective binding sites must also be considered.

Solvent selection is another major issue. Conventional MIP synthesis often relies on organic porogens such as chloroform, acetonitrile, toluene, or methanol, which may raise safety and environmental concerns. Greener alternatives include water, ethanol, aqueous mixtures, supercritical fluids, DES, and selected ionic liquids [194,195,196], whose respective merits and limitations are assessed in Section 10.3. DES and water-compatible imprinting media are especially attractive when they enable efficient template–monomer interactions without compromising binding-site fidelity. [196,197].

Energy efficiency can be improved through photopolymerization, visible-light initiation, microwave-assisted synthesis, or room-temperature polymerization [194,195]. These approaches may reduce reaction time and energy input, but they must be optimized to avoid incomplete polymerization, heterogeneous network formation, or residual initiator contamination. Similarly, safer initiators, lower monomer excess, and reduced solvent use can improve the environmental profile of MIP synthesis, provided that analytical performance is preserved.

Design for degradation and recyclability is an emerging but still underdeveloped concept in MIP biosensing. Incorporating degradable crosslinkers, hydrolysable linkages, reversible covalent bonds, or recyclable support materials could enable controlled disassembly or easier end-of-life processing. However, degradation must be compatible with sensor shelf life, binding-site stability, and operational robustness. Thus, sustainable MIP synthesis requires a balanced design strategy rather than a simple replacement of conventional components.

### 10.2. Bio-Based and Biodegradable MIP Matrices

Bio-based and biodegradable polymer matrices represent a promising route toward more sustainable MIP biosensors. Natural polymers offer functional groups that can participate in molecular recognition, including hydroxyl, amine, carboxyl, and amide groups. They also provide hydrophilicity, biocompatibility, and processability in aqueous media, which are particularly useful for biosensing in biological or environmental matrices.

Chitosan is one of the most attractive natural polymers for MIP design because its amine and hydroxyl groups can interact with templates through hydrogen bonding, electrostatic interactions, and coordination with metal ions. Its film-forming ability and compatibility with mild processing conditions make it suitable for electrochemical and optical sensor interfaces [198]. However, chitosan-based MIPs require careful control of pH, degree of deacetylation, swelling, and mechanical stability.

Cellulose and cellulose derivatives, including hydroxyethyl cellulose, carboxymethyl cellulose, cellulose acetate, and nanocellulose, provide renewable scaffolds with good mechanical properties and high hydrophilicity. Their abundant hydroxyl groups enable chemical modification, crosslinking, and integration with conductive or optically active components. These matrices are particularly relevant for paper-based sensors, disposable strips, and environmentally oriented sensing platforms [199,200].

Protein-derived or peptide-compatible matrices such as gelatin, silk fibroin, and related biopolymers may be useful for imprinting fragile biomolecules because they provide soft, hydrated, and biocompatible environments. However, their use in MIP biosensing requires careful stabilization, since excessive swelling, biodegradation, or batch variability can affect binding-site reproducibility.

Synthetic biodegradable polymers such as polylactide (PLA), polycaprolactone (PCL), and related aliphatic polyesters offer additional opportunities for transient or disposable sensor platforms. Their main advantage is degradability and compatibility with established polymer processing methods. However, their direct use as MIP matrices for high-performance biosensing remains less mature than conventional acrylate-, methacrylate-, or electropolymerized systems. The field has yet to establish PLA- and PCL-based MIP biosensors as a mature technology, and they are best regarded for now as an emerging option awaiting systematic validation.

Overall, bio-based and biodegradable MIPs can contribute to safer and more sustainable sensing formats, especially for environmental monitoring, food analysis, and short-term disposable diagnostics. However, their adoption requires systematic evaluation of binding performance, stability, template bleeding, degradation products, leachables, and reproducibility under real operating conditions.

#### Hybrid Bio-Synthetic Matrices

A realistic strategy for sustainable MIP biosensing is the development of hybrid bio-synthetic matrices. In these systems, natural or biodegradable polymers provide a renewable, hydrophilic, or biocompatible scaffold, while synthetic conductive or nanostructured components provide the electronic, optical, or mechanical functionality required for sensing.

Examples pair a biopolymer scaffold with the conductive nanofillers of Section 4.5, so that the synthetic phase supplies charge transfer and signal amplification while reducing the fraction of fully synthetic polymer required. They may also enable aqueous fabrication, lower solvent consumption, and compatibility with flexible or paper-based substrates [199,201].

However, hybridization does not automatically guarantee sustainability: the environmental impact of the conductive polymers, nanomaterials, metal nanoparticles, or MXenes added to the biopolymer scaffold must be weighed against its renewable character—an assessment deferred to the full material–device evaluation of Section 10.4.

Hybrid bio-synthetic MIPs therefore represent a pragmatic compromise between green design and analytical performance. They are especially promising when the biopolymer component improves aqueous compatibility, antifouling behaviour, or biodegradability, while the synthetic phase provides robust transduction. Future progress will depend on designing such composites through lifecycle-aware material selection rather than performance optimization alone.

### 10.3. Green Solvents and Polymerization Media

The replacement or reduction in volatile and hazardous organic solvents is one of the most important objectives in sustainable MIP synthesis. As already noted in Section 10.1, the organic porogens conventionally used to stabilise template–monomer complexes and generate porous networks raise concerns of toxicity, flammability, waste generation, and environmental persistence; greener media must therefore reduce these impacts without eroding imprinting efficiency.

Water and aqueous mixtures are the most attractive media for biosensing-oriented MIPs, especially when the final device is intended to operate in biological fluids. However, direct aqueous imprinting is constrained by the water-competition effects set out in Section 2.5, which is why water-compatible formulations rely on the hydrophobic, ionic, boronate, coordination, or hydrophilic-monomer strategies detailed in Section 3.7 and Section 4.1.3.

DES have emerged as promising alternatives because their polarity, hydrogen-bonding ability, viscosity, and solvating power can be tuned by varying the hydrogen-bond donor and acceptor components. In MIP synthesis, DES may act as porogens, functional media, or stabilizers of template–monomer interactions, or green eluents for template removal [196,197,202]. Nevertheless, DES should be evaluated case by case, since their viscosity, template extraction efficiency, toxicity, and compatibility with sensor fabrication can strongly affect both sustainability and analytical performance.

**Figure 22:**
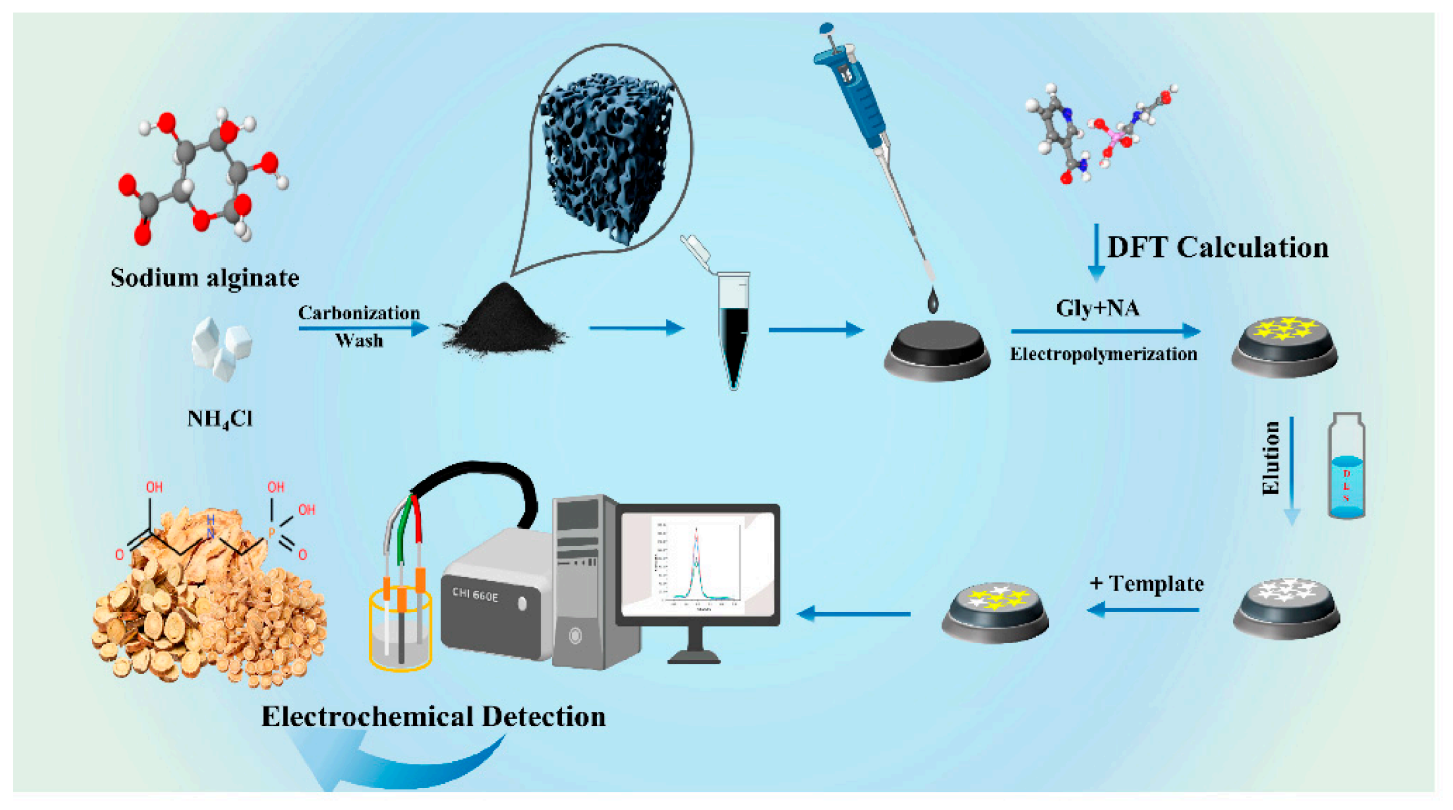
Workflow for the fabrication of a molecularly imprinted electrochemical sensor integrating three complementary green-design elements: a hierarchical porous carbon substrate obtained by co-carbonization of the biomass precursor sodium alginate, a functional monomer (nicotinamide) selected by DFT rather than by empirical screening, and a DES employed as the eluent for template removal in place of conventional organic or strongly acidic/basic media. Reproduced from Wang et al. [202], Polymers 2025, under CC BY 4.0.

A representative implementation of this approach is shown in Figure 22, where a DES is used as the elution medium for template removal within a MIP sensor built entirely on a biomass-derived carbon platform [202].

Ionic liquids (ILs) can also be useful in MIP synthesis, sometimes acting simultaneously as solvent, porogen, functional monomer, or conductivity-enhancing component [97]. However, ILs should not be described as intrinsically green. Their environmental profile depends on cation/anion structure, biodegradability, toxicity, persistence, synthesis route, and recovery. Bio-derived, task-specific, or recyclable ILs may contribute to greener MIP design, but their use must be justified by both analytical benefit and environmental assessment.

Other solvent-reduction strategies include emulsion imprinting, miniemulsion polymerization, surface-confined polymerization, photopolymerization in thin films, and, in selected cases, supercritical CO_2_ processing. These approaches can reduce solvent consumption, improve control over particle size or film morphology, and facilitate integration into sensor platforms [97,195]. However, their practical impact depends on scalability, energy demand, template removal, and compatibility with real device fabrication.

### 10.4. Life-Cycle Assessment and Eco-Design

A life-cycle perspective is essential for evaluating the real sustainability of MIP-based sensors. Although green monomers or benign solvents may reduce the environmental burden of synthesis, the overall footprint of a device also depends on substrate choice, transducer fabrication, nanomaterial production, energy consumption, packaging, use phase, and end-of-life disposal.

**Figure 23:**
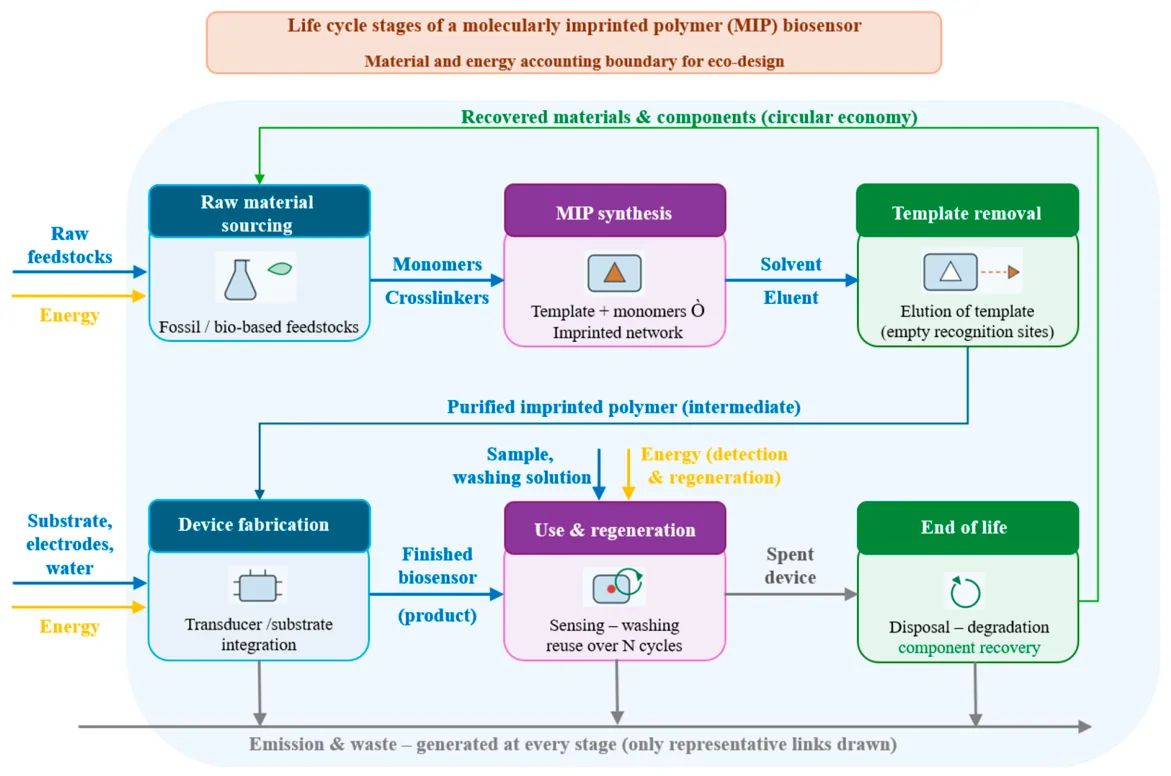
Life-cycle stages of a molecularly imprinted polymer biosensor, used here as the accounting boundary for eco-design: raw-material sourcing → MIP synthesis → template removal → device fabrication → use and regeneration → end of life (disposal, degradation, or component recovery). Arrows denote the material and energy flows to which a stated functional unit (per sensor, per analysis, per usable cycle, or per quantity of analyte) is applied. The diagram is schematic and does not represent quantitative LCA data.

Life-cycle assessment (LCA) can, in principle, quantify material and energy flows across the main stages of a MIP sensor lifecycle: raw-material sourcing, polymer synthesis, template removal, device fabrication, operation, regeneration, and disposal or recycling (Figure 23). Because these burdens are meaningful only once normalised, every environmental claim should be referred to an explicit functional unit—per sensor produced, per analysis performed, per usable regeneration cycle, or per quantity of analyte determined—rather than to polymer mass alone, since the same device can appear efficient or wasteful depending on the basis chosen. For disposable sensors and wearable devices this normalisation is particularly important, because large-scale use may generate significant polymeric and electronic waste.

At present, quantitative LCA studies specifically focused on MIP biosensors remain limited. Therefore, numerical claims regarding the percentage contribution of MIP synthesis, solvent handling, or monomer production to total energy use should be avoided unless supported by a dedicated LCA. More defensible sustainability indicators include solvent volume, solvent hazard, reaction temperature and time, monomer renewability, device mass, number of usable cycles, recoverability of valuable components, and waste generated per analytical measurement; established greenness-scoring frameworks for analytical methods provide a practical starting point [203].

Eco-design strategies for MIP sensors should prioritize the reduction in material use, simplified device architecture, aqueous or low-solvent fabrication, modular components, recyclable substrates, and longer operational lifetime when reuse is feasible. In disposable formats, the use of paper, cellulose derivatives, biodegradable supports, or minimal-electronics architectures may reduce waste generation. In higher-value devices, regeneration and component recovery may provide greater sustainability benefits than single-use biodegradability.

Combining green polymer chemistry with sustainable device engineering is therefore essential. A MIP formulation should not be considered sustainable solely because one component is bio-based or biodegradable; instead, the complete material–device system must be evaluated in terms of performance, safety, durability, recyclability, and end-of-life fate.

### 10.5. Transient and Degradable MIP Sensors

Transient and degradable MIP sensors represent an attractive future direction for sustainable biosensing, particularly for disposable diagnostics, temporary environmental monitoring, and short-term wearable devices. In this concept, the sensing material or device is designed to degrade, dissolve, or disassemble after completing its analytical function, thereby reducing environmental accumulation and electronic waste.

The candidate matrices are those already surveyed in Section 10.2—modified polysaccharides, gelatin, silk-derived materials, polylactide, and polycaprolactone—whose relevance here lies specifically in their hydrolytic or enzymatic degradability. These materials could support the development of sensors with improved end-of-life profiles, especially when combined with biodegradable substrates and minimal electronic components. However, degradability introduces a central design trade-off: the polymer must remain stable during storage and operation, but degradable after use under defined conditions.

The integration of MIPs with transient electronics is still at an early stage. Transient conductive elements based on dissolvable metals or degradable substrates are well established in the broader field of bioresorbable electronics [204,205], but their direct combination with molecularly imprinted recognition layers remains less mature. Therefore, transient MIP sensors belong, at present, to the exploratory frontier of the field rather than to its established repertoire.

**Figure 24:**
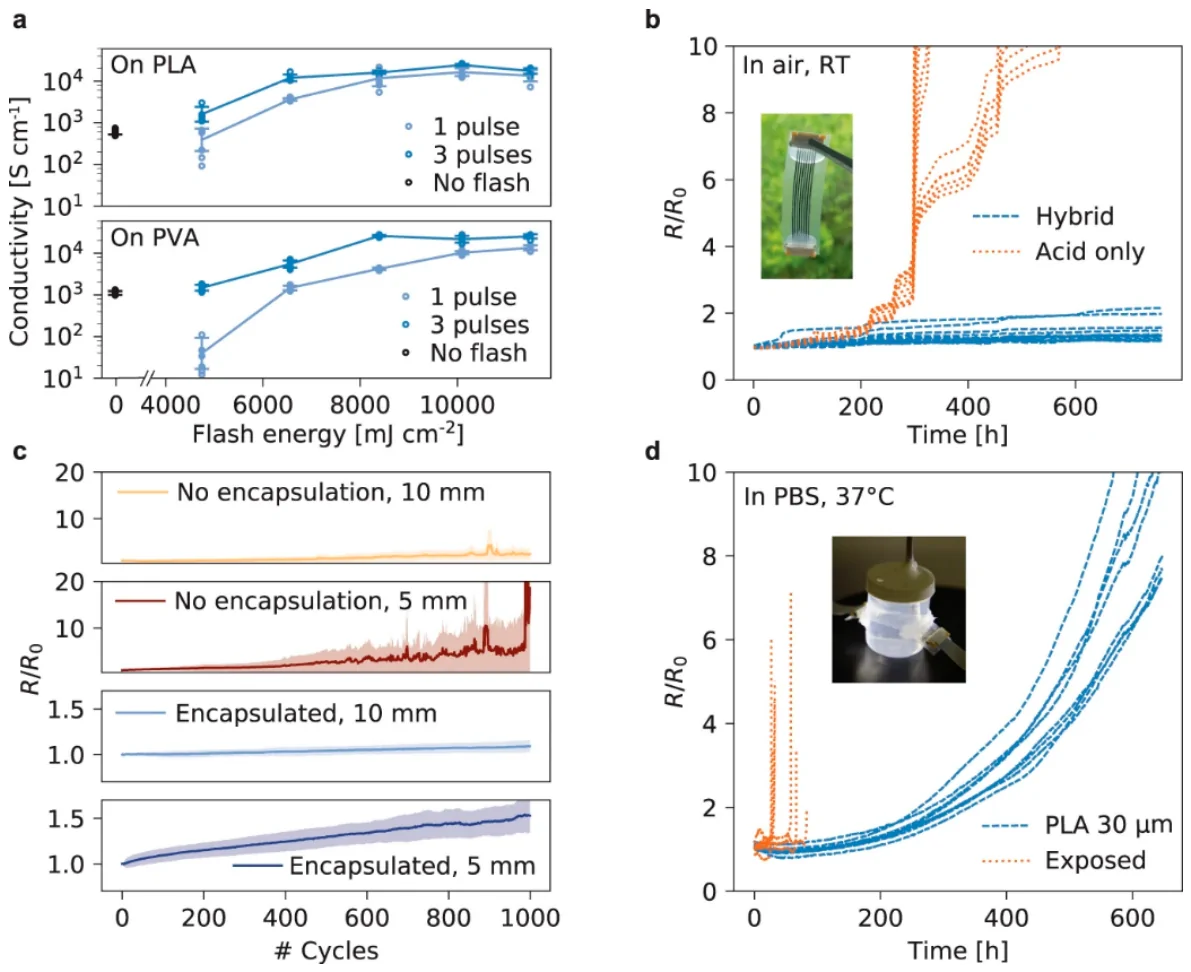
Stability–degradability trade-off in transient conductors, illustrated for printed zinc traces on bioresorbable substrates. (**a**) Electrical conductivity of sintered Zn traces on poly(vinyl acetate) and polylactide as a function of photonic pulse energy and pulse number. (**b**) Resistance drift of unencapsulated Zn tracks stored under ambient conditions over 31 days, comparing sintering protocols. (**c**) Resistance change in Zn lines on polylactide under repeated bending at 5 mm and 10 mm radii, with and without polylactide encapsulation. (**d**) Resistance evolution of encapsulated and unencapsulated Zn tracks immersed in phosphate-buffered saline at 37 °C, showing that the encapsulation layer sets the operational lifetime before degradation proceeds. Reproduced from Fumeaux and Briand [205], npj Flexible Electronics 2023, under CC BY 4.0.

Key challenges include maintaining binding-site fidelity in degradable matrices, avoiding template bleeding, controlling degradation products, ensuring biocompatibility, and preserving calibration stability over the intended lifetime. Future transient MIP platforms will need to reconcile analytical selectivity with predictable degradation, safe disposal, and reproducible device performance. The nature of this trade-off is illustrated in Figure 24 for printed zinc conductors on bioresorbable substrates, where encapsulation governs the transition from a stable operational window to controlled degradation in physiological medium [205]; equivalent lifetime data for imprinted recognition layers on such substrates are not yet available.

### 10.6. Recyclability and Regeneration of MIP Materials

One of the main advantages of MIPs over biological receptors is their chemical robustness, which can allow repeated binding and regeneration cycles. From a sustainability standpoint, however, reuse is a genuine saving only if it is real: long-term reusability must be demonstrated rather than assumed, following the cycle-resolved reporting set out in Section 9.5, and additionally verifying the absence of template bleeding across cycles [197,202].

As already stressed for wearable formats in Section 8.6, regeneration must be compatible with the device: laboratory washing protocols rarely translate to wearable, microfluidic, or field-deployable sensors, where solvent exposure, user safety, and small-volume operation impose practical constraints. Mild aqueous regeneration, reversible binding chemistries, and integrated microfluidic rinsing are therefore more realistic for practical devices than aggressive solvent extraction [202].

Recycling of MIP-based devices remains comparatively underexplored. In principle, conductive fillers, noble-metal nanoparticles, carbon nanomaterials, or reusable substrates could be recovered from some sensor architectures. However, polymer–nanomaterial composites are often difficult to separate cleanly, and recycling processes may consume energy or solvents that offset their environmental benefit. Thermoplastic or dynamically crosslinked MIP systems may eventually enable reprocessing or material recovery, but this remains a developing area.

Closed-loop MIP production and recycling should therefore be considered a long-term sustainability goal. In the near term, the most practical strategies are likely to be reducing material consumption, increasing sensor lifetime when reuse is safe, selecting lower-impact substrates, improving regeneration protocols, and designing devices with fewer inseparable material classes.

### 10.7. Sustainable Performance Benchmarking

To quantify environmental progress, sustainability metrics (Table 5) should complement analytical performance benchmarks (Section 9).

Key indicators include:

Adopting such eco-performance indicators, in line with established greenness-scoring frameworks [203] and with the principles of green molecular imprinting [194], ensures that future MIP designs optimize both analytical and environmental dimensions. Consistent with the caution expressed in Section 9.4 and Section 10.4, these indicators orient eco-design rather than defining pass/fail limits, and are most useful when the underlying quantities are transparently measured and reported for each device.

Sustainability, reproducibility, and analytical performance are thus three constraints on a single design problem; how the field might satisfy them simultaneously is the subject of the concluding section.

## 11. Outlook and Future Perspectives

Since the introduction of molecular imprinting for chromatographic separation in the 1970s (Introduction), MIPs have evolved from a concept in synthetic recognition chemistry into a versatile class of materials for selective binding, separation, and sensing. Their transition from bulk sorbents to nanostructured, surface-confined, and multifunctional interfaces has paralleled advances in polymer science, nanotechnology, biointerfaces, and device engineering. In biosensing, MIPs are increasingly considered robust synthetic alternatives or complements to biological receptors, offering tunable selectivity, chemical stability, reusability, and compatibility with miniaturized transducers.

Nevertheless, the next stage of MIP biosensing will depend on whether these materials can move beyond proof-of-concept demonstrations toward reproducible, standardized, and application-validated devices. Several converging research directions are expected to define this transition.

### 11.1. Data-Driven and AI-Assisted Development

The computational tools introduced in Section 2.8 are likely to shift from a supporting to a structuring role. It is useful, however, to distinguish four functionally distinct roles that are often grouped under “AI-assisted MIP development”, since they differ markedly in maturity and in data requirements. The first is the selection of functional monomers, crosslinker ratios, and synthesis conditions, in which ML correlates molecular descriptors with imprinting quality [13]. The second is signal processing, in which algorithms correct baseline drift, improve signal-to-noise ratio, and extract analyte-specific features from electrochemical or optical readouts [15]. The third is multi-analyte classification, in which pattern-recognition models are used to deconvolute the overlapping responses of hierarchical and multi-template systems (Section 7.5). The fourth is benchmarking and stability prediction, in which models trained on curated performance repositories (Section 9.7) could forecast cross-reactivity, drift, and shelf life. Of these, signal processing is at present the most mature, whereas predictive design and stability forecasting remain largely aspirational and dataset-limited. MD, DFT, and docking already reduce the empirical burden of monomer and porogen selection, while machine-learning models trained on published IFs can rank candidate formulations before any synthesis is attempted. Two conditions, however, must be met before these methods become genuinely predictive. The first concerns the data. The data problem set out in Section 9.7—small, heterogeneous, positively biased datasets—makes standardised, negative-result-inclusive reporting a prerequisite for predictive modelling rather than an afterthought. Even where data are adequate, credible predictive claims further require validation on external, independently generated datasets rather than random train–test splits, explicit prevention of data leakage between related template families, reporting of predictive uncertainty rather than point estimates, and a clear specification of the model’s applicability domain. The second concerns scope: current descriptors represent small-molecule templates far better than proteins, virions, or cells, so the target classes for which imprinting is hardest (Section 7.4) remain those for which computational guidance is weakest. A realistic expectation for the coming decade is therefore closed-loop optimisation for small-molecule MIPs, as well as model-assisted rather than model-driven design for macromolecular targets.

### 11.2. Bioinspired and Hybrid Recognition Systems

The boundary between synthetic and biological recognition is becoming increasingly fluid. Hybrid systems that combine MIPs with aptamers, peptides, enzymes, antibodies, or biomimetic motifs may offer complementary advantages: biological components provide high-affinity recognition, while the polymer matrix contributes mechanical stability, chemical robustness, and protection against degradation.

Such biohybrid architectures are particularly promising for difficult targets such as proteins, nucleic acids, viruses, and cell-surface markers. They may also support more selective interfaces for electrochemical, optical, and transistor-based biosensing. Nevertheless, their practical value will depend on whether the hybrid design truly improves stability, selectivity, or manufacturability relative to either component alone. Future work should therefore focus on systematic comparisons between pure MIP, pure bioreceptor, and hybrid MIP–bioreceptor systems.

### 11.3. The Vision Ahead

Looking forward, the evolution of MIP biosensing can be viewed as a transition from static synthetic receptors toward engineered functional interfaces. Future MIP platforms are expected to combine selective recognition with nanostructured transducers, antifouling biointerfaces, flexible substrates, data-driven design, and sustainable fabrication routes.

Within the next decade, the most significant advances are likely to arise on the materials side—computationally guided selection of monomers, crosslinkers, and imprinting media; surface-confined and epitope-based imprinting for complex biological targets; improved antifouling, regeneration, and long-term stability in real samples; and greener, potentially degradable materials for disposable devices. On the device side, progress is expected in the integration of MIPs with flexible, wearable, and microfluidic formats; in multimodal platforms combining electrochemical, optical, and mass-sensitive readouts; and in standardized benchmarking supported by open performance datasets.

This trajectory is the one announced in the title of this Review: MIPs are ceasing to be artificial receptors and are becoming integrated biointerfaces—programmable polymeric layers that connect molecular recognition with signal transduction, device integration, and sustainable materials design. Their future impact will depend not only on achieving high sensitivity and selectivity, but also on demonstrating reproducibility, manufacturability, environmental responsibility, and reliable performance in real-world conditions.

## Figures and Tables

**Table 1:** Indicative comparison of biological, biomimetic, and synthetic recognition elements. Values are reported ranges from the 2023–2025 literature and depend strongly on formulation, target, transducer, and experimental conditions; they are intended as orientation, not as universal or benchmarked figures. K_d_ denotes the thermodynamic affinity of the recognition pair, whereas the LOD is a system-level property that also depends on the transducer, noise, amplification, and statistical treatment (Section 5.7, Section 9.3 and Section 9.4).

Property	Antibodies	Enzymes	Aptamers	Bulk MIPs	Surface-Imprinted MIPs/NanoMIPs
Recognition principle	Conformational recognition with induced-fit contributions; sensitive to higher-order-structure (HOS) perturbations [32]	Catalytic recognition (binding coupled to substrate turnover); active-site specific [32]	Conformational selection: adaptive folding on target binding; sequence- and structure-dependent [33]	Predominantly pre-organized cavities with a heterogeneous site population; largely, but not exclusively, static	Pre-organized, surface-confined cavities; more homogeneous and accessible sites [34]
Recognition affinity (K_d_), thermodynamic	Typically pM–nM; best therapeutic mAbs reported down to fM [35]	Substrate Km typically µM–mM; inhibitor Ki down to nM (affinity is not the primary design goal) [32]	Typically pM–nM depending on selection [36]	Typically µM–nM (reported range) [34]	Typically nM–pM; best nanoMIPs approach antibody-like Kd [34,37]
Analytical sensitivity/LOD (system-level)	Immunoassays typically pM–fM depending on transduction/amplification [35]	Enzymatic sensors analyte-dependent, often nM–µM [32]	Aptasensors typically pM–fM depending on platform [38]	Often nM–µM [35]	Down to pM–fM in optimized platforms; strongly matrix- and transducer-dependent (Section 5.7 and Section 9.4) [34,37]
Thermal stability	Typically denature/aggregate above ~60–80 °C; cold-chain storage usually required [32]	Activity usually confined to a narrow window; many lose activity above ~40–60 °C (enzyme-dependent) [32]	Reversible thermal denaturation/refolding; tolerate repeated thermal cycling [39]	Generally stable over a wide range; upper limit set by polymer decomposition, often well above 150 °C depending on backbone [37,40]	Polymer-limited stability as for bulk; thin films and hydrogel formats may be less robust depending on formulation [37]
Chemical stability	pH- and protease-sensitive; degradation under organic solvents/extremes, extent depending on exposure time [32]	pH-, solvent-, and inhibitor-sensitive; activity loss under non-physiological conditions [32]	Nuclease-sensitive unless chemically modified; otherwise chemically robust [41]	Generally resistant across a broad pH range (e.g., ~3–9 reported) and to many organic solvents, depending on formulation [37]	Comparable robustness; hydrogel/zwitterionic formats trade some chemical resistance for aqueous compatibility [37]
Regeneration/reusability	Limited; harsh elution degrades binding; frequently single-use [32]	Limited; often consumed or inactivated; regeneration difficult [32]	Readily regenerable by denaturation–refolding; many cycles [42]	Regenerable by washing, but site heterogeneity may cause template bleeding [40]	Typically good (e.g., ~90% signal retention over multiple cycles reported); protocol-dependent (Section 5.6, Section 8.6 and Section 9.5) [40]
Batch reproducibility	Intrinsic batch-to-batch variability of biological production [32,35]	Batch variability in activity and purity [32]	High, via defined-sequence chemical synthesis [43]	Variable; grinding/sieving gives heterogeneous site density (Section 3.1) [40]	Improved but still protocol-sensitive; an open reproducibility issue (Section 9.1) [34]
Cost and scalability	High; complex biotechnological production and purification [32,35]	Moderate–high; production/purification dependent [32]	Moderate; scalable chemical synthesis after SELEX [39]	Low-cost, simple synthesis, but limited device integration [40]	Low–moderate; scalable and compatible with low-cost electrodes and printing [34]

**Table 3:** Representative miniaturised and wearable MIP platforms, with substrate, target biofluid, analytical figures of merit, and integration route.

Platform	Substrate	Biofluid	Target Analyte	LOD	Response Time	Integration	Ref./Status
Flexible dual-channel MIP electrode	PET	Sweat	Lactate, cortisol	lactate ~1 mM; cortisol ~1 nM	n.r	on-body, wired read-out	[171] experimental (single study)
eMIP nanoparticle electrode	Screen-printed electrode	Artificial ISF (sweat-analogue)	Glucose	~3 × 10^−8^ M (26 nM)	<30 s	wired (proof-of-application)	[172]—proof-of-concept
MIP + thread-microfluidic floss (graphene electrode, PPy/PB)	Thread/graphene	Saliva	Cortisol	~1 × 10^−13^ M (range ~3 × 10^−13^–3 × 10^−8^ M)	~11–12 min	capillary (thread) microfluidics + wireless readout	[163]—experimental (validated vs. ELISA on human saliva)
TENG–MIP hybrid (3-APBA imprinted)	PVDF/graphene electrode; PVDF–Cu triboelectric counter-layer	Artificial sweat (on-skin feasibility demo)	Lactate (self-powered)	not reported (working range 0–20 mM; sensitivity 5.18 μA cm^−2^ mM^−1^)	n.r. (2 Hz contact actuation)	Self-powered (triboelectric; drives up to 10 LEDs)	[168]—experimental (single study)

**Table 4:** Proposed reporting checklist of figures of merit for MIP biosensors. The table specifies which quantities should be reported, and under which conditions, rather than pass/fail thresholds; the single orientation value retained (IF > 3 for non-covalent systems) is author-proposed and target-dependent, not a community-wide standard, and is discussed in full in Section 2.4 [26]).

Parameter	Definition/Relevance	What to Report and Under Which Conditions
Detection capability (LOB/LOD/LOQ)	Lowest reliably distinguishable/quantifiable concentration	Value from a stated statistical criterion (not visual S/N alone), reported in buffer and in the intended matrix; state replicate number and confidence level [185]
Response time	Time to reach 90% of steady-state signal	Report with the 90% definition and the stirring/flow and temperature conditions; sub-10 s is a typical target for real-time use, not a requirement
IF	Q_MIP_/Q_NIP_ under identical conditions—measures the imprinting effect, not selectivity	Report with binding conditions and the matched NIP; IF > 3 is an author-proposed orientation for non-covalent systems (Section 2.4) [26]
Selectivity/cross-reactivity	Discrimination of the target from structurally related interferents (distinct from IF)	Report the target:interferent response ratio (or cross-reactivity %) at stated, realistic interferent concentrations; do not substitute IF for this
Reusability/regeneration	Performance retention across binding–regeneration cycles	Report number of cycles together with signal retention, baseline recovery, calibration-slope drift, and selectivity after cycling (Section 9.5)
Operational/storage stability	Retention of sensitivity under stress and storage	Report retained response with the mechanical/chemical stress, temperature/humidity, and duration specified
Environmental stability	Signal drift across the operating window	Report drift with the pH, temperature and ionic-strength range over which it was measured

**Table 5:** Sustainability indicators to complement the reporting checklist of Table 4. As in Table 4, entries specify quantities to be measured and reported under stated conditions rather than pass/fail thresholds; none are community-wide standards (Section 9.4 and Section 10.4).

Sustainability Metric	Definition	What to Report
Renewable content	Fraction of bio-based mass/carbon in the polymer	The %, with the basis (mass vs. bio-based carbon) and accounting method
Solvent greenness	Solvent selection score	The score with the framework used (e.g., CHEM21/GSK), for every solvent in synthesis and template removal [203]
Energy demand	Energy per functional unit	Energy per device or per analytical measurement (not per mg of polymer alone), with system boundaries stated (Section 10.4)
Recyclability/recoverability	Fraction of valuable components recovered	The recovered fraction, which components, and the recovery process and its own footprint
Degradation (mass loss)	Mass loss under defined conditions	Mass loss with medium, temperature and duration—note that mass loss is a proxy and does not by itself demonstrate biodegradation or mineralization; report mineralization (e.g., CO_2_ evolution/standardized test) where that claim is made

## Data Availability

No new data were created or analyzed in this study. Data sharing is not applicable to this article.

## Abbreviations

The following abbreviations are used in this manuscript:

AAm	acrylamide
AI	artificial intelligence
DES	Deep eutectic solvents
DFT	density functional theory
DPV	differential pulse voltammetry
DVB	divinylbenzene
EGDMA	ethylene glycol dimethacrylate
IF	imprinting factor
LOD	limits of detection
MAA	methacrylic acid
MD	molecular dynamics
MIP(s)	molecularly imprinted polymer(s)
ML	machine learning
NIP	non-imprinted polymer
SAW	surface acoustic wave
SPE	solid phase extraction
SPR	surface plasmon resonance
TRIM	trimethylolpropane trimethacrylate
4-VP	4-vinylpyridine
